# Polygenic basis and biomedical consequences of telomere length variation

**DOI:** 10.1038/s41588-021-00944-6

**Published:** 2021-10-05

**Authors:** Veryan Codd, Qingning Wang, Elias Allara, Crispin Musicha, Stephen Kaptoge, Svetlana Stoma, Tao Jiang, Stephen E. Hamby, Peter S. Braund, Vasiliki Bountziouka, Charley A. Budgeon, Matthew Denniff, Chloe Swinfield, Manolo Papakonstantinou, Shilpi Sheth, Dominika E. Nanus, Sophie C. Warner, Minxian Wang, Amit V. Khera, James Eales, Willem H. Ouwehand, John R. Thompson, Emanuele Di Angelantonio, Angela M. Wood, Adam S. Butterworth, John N. Danesh, Christopher P. Nelson, Nilesh J. Samani

**Affiliations:** 1 Department of Cardiovascular Sciences, University of Leicester, Leicester, UK; 2 NIHR Leicester Biomedical Research Centre, Glenfield Hospital, Leicester, UK; 3 British Heart Foundation Cardiovascular Epidemiology Unit, Department of Public Health and Primary Care, University of Cambridge, Cambridge, UK; 4 National Institute for Health Research Blood and Transplant Research Unit in Donor Health and Genomics, University of Cambridge, Cambridge, UK; 5 British Heart Foundation Centre of Research Excellence, University of Cambridge, Cambridge, UK; 6 School of Population and Global Health, University of Western Australia, Perth, Western Australia Australia; 7 Program in Medical and Population Genetics, Broad Institute of MIT and Harvard, Cambridge, MA USA; 8 Center for Genomic Medicine, Massachusetts General Hospital, Boston, MA USA; 9 Department of Medicine, Harvard Medical School, Boston, MA USA; 10 Cardiology Division, Department of Medicine, Massachusetts General Hospital, Boston, MA USA; 11 Division of Cardiovascular Sciences, University of Manchester, Manchester, UK; 12 Department of Haematology, University of Cambridge, Cambridge, UK; 13 NHS Blood and Transplant, Cambridge, UK; 14 University College London Hospitals NHS Foundation Trust, London, UK; 15 Department of Health Sciences, University of Leicester, Leicester, UK; 16 Health Data Research UK Cambridge, Wellcome Sanger Institute, EMBL-European Bioinformatics Institute and University of Cambridge, Cambridge, UK; 17 The Alan Turing Institute, London, UK; 18 Department of Human Genetics, Wellcome Sanger Institute, Hinxton, UK

**Keywords:** Genome-wide association studies, Genetics research

## Abstract

Telomeres, the end fragments of chromosomes, play key roles in cellular proliferation and senescence. Here we characterize the genetic architecture of naturally occurring variation in leukocyte telomere length (LTL) and identify causal links between LTL and biomedical phenotypes in 472,174 well-characterized UK Biobank participants. We identified 197 independent sentinel variants associated with LTL at 138 genomic loci (108 new). Genetically determined differences in LTL were associated with multiple biological traits, ranging from height to bone marrow function, as well as several diseases spanning neoplastic, vascular and inflammatory pathologies. Finally, we estimated that, at the age of 40 years, people with an LTL >1 s.d. shorter than the population mean had a 2.5-year-lower life expectancy compared with the group with ≥1 s.d. longer LDL. Overall, we furnish new insights into the genetic regulation of LTL, reveal wide-ranging influences of LTL on physiological traits, diseases and longevity, and provide a powerful resource available to the global research community.

## Abstract (web summary)

Genome-wide association and Mendelian randomization analyses in the UK Biobank identify genetic variants associated with leukocyte telomere length and highlight putative causal links between telomere length and biomedical phenotypes.

## Main

Telomeres are nucleoprotein complexes at chromosome ends that shorten with each cell division and play key roles in maintaining chromosomal integrity^1^. Telomere length (TL) is heritable, but there is incomplete understanding of its genetic determination^2–4^. Extreme shortening of telomeres, due to rare mutations in telomere regulatory genes, causes premature aging syndromes^5^. By contrast, more subtle inter-individual variation in TL has been associated with the risk of certain cancers, coronary artery disease and other common age-associated adult conditions^6–8^. Although there has been much interest in a shorter TL as a biomarker of older biological age^9^, it is now apparent that the relationship between TL and disease risk is complex, as both shorter TL and longer TL have been associated with higher risks of different age-associated diseases^4,10–12^. Population biobanks afford opportunities to provide insight into the genetic architecture of TL and its links with biomedical phenotypes. Progress has been limited, however, because most biobanks have not been able to combine large-scale TL measurement, detailed genomic characterization, extensive biomedical phenotyping and exceptional statistical power.

Here we interrogate a powerful population resource of peripheral leukocyte TL (LTL) measurements, a practical measure of TL that correlates well with TL across different tissues^13^ within individuals, that we created in 472,174 well-characterized participants in the UK Biobank (UKB)^14,15^. We increase knowledge of the genetic architecture of LTL several-fold, including identification of multiple new rare and lower-frequency variants associated with LTL. Using the principle of Mendelian randomization (MR), we find evidence to support causal roles for LTL with multiple physiological traits and diverse diseases. We also estimate that people with shorter LTL have a lower life expectancy.

## Results

### Genetic determinants of TL

**Fig. 1:**
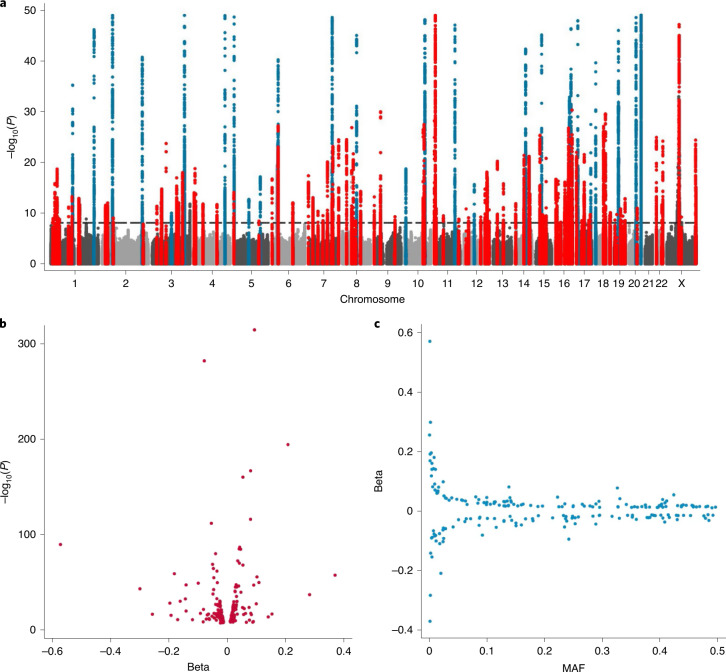
Conditionally independent genome-wide significant hits. **a**, Manhattan plot curtailed at *P* < 1 × 10^−50^. We highlight the regions containing our 197 sentinel variants that are genome-wide significant (*P* < 8.31 × 10^−9^; horizontal dashed reference line) in the exact joint conditional model (Supplementary Table 1). We defined the region as known (blue) if a previous variant within 1 Mb of our sentinel has been reported at either genome-wide significance or at an FDR threshold of <5%. Regions were considered new (red) if a variant within 1 Mb of our sentinel that reached genome-wide significance was not previously identified. Non-significant variants are shown in either light or dark gray on alternate chomosomes. **b**, The estimated effect sizes (beta) against the *P* value from the GWAS analysis. **c**, Estimated effect sizes for the minor allele (beta) against the MAF from all participants in the GWAS.

**Extended Data Fig. 1:**
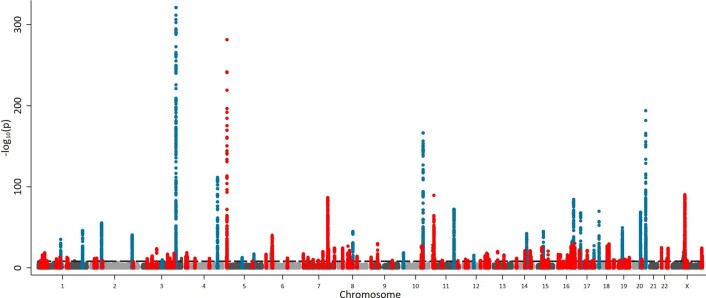
Manhattan plot unrestricted by *P* value. We highlight our 197 sentinel variant regions that are genome-wide significant (*P* < 8.31 × 10^−9^, horizontal dashed reference line) in the exact joint conditional model (Supplementary Table 1). We define the region as known (blue) if a previous variant within 1 Mb of our sentinel has previously been reported at genome-wide significance. We consider our other regions novel (red) as the first evidence of a variant within 1 Mb of our sentinel that reaches genome-wide significance.

We used an established quantitative PCR assay to obtain LTL measurements in 472,174 UKB participants, undertook multiple quality checks to control and adjust for technical factors, and confirmed associations with known LTL-associated phenotypes such as age, sex and ethnicity, as detailed elsewhere^15^. We also made paired LTL measurements from DNA taken at two time points (mean interval: 5.5 years) in 1,351 participants to enable the calculation of, and correction for, regression dilution (Methods)^15^. Using standard genome-wide association analyses and exact joint conditional modeling in 464,716 participants with data available on 19.4 million imputed variants (minor allele frequency (MAF) ≥ 0.1%; Methods and Supplementary Fig. 1), we identified 197 independent associations for LTL (Supplementary Table 1) exceeding a genome-wide significance threshold of *P* < 8.31 × 10^−9^ (Methods). This threshold was set to account for the inclusion of low-frequency variants in the analysis^16^. The sentinel variants were located within 138 genomic loci (>500 kb between sentinels), of which 108 were new (>1 Mb from a previously reported sentinel) and 30 were previously reported at genome-wide significance or a false-discovery rate (FDR) of <5% (Fig. 1a, Supplementary Table 1, Extended Data Fig. 1 and Supplementary Data)^3,4,17^. Collectively, the 197 variants explained 4.54% of the variance in LTL. In total, 714 independent variants—the majority of which are new—were associated with LTL at an FDR threshold of <1% (Supplementary Table 2), increasing the amount of variance explained to 5.64%. The estimated heritability for LTL explained by all variants genome-wide was 8.1% (s.d. = 0.26).

Twenty of the genome-wide significant sentinel variants identified here for the first time (Supplementary Table 1) were lower-frequency variants (MAF < 1%), including new association signals at several known loci (including *TERT1*, *TERF1* and *RTEL1*) and new loci (such as *EXOSC10*, *SMC4* and *SRSF6*). The estimated effects of the sentinel variants were generally modest—that is, <0.2 s.d. per allele (Fig. 1b,c). Most of the loci with the strongest evidence for association (*P* < 1 × 10^−50^) had been previously identified, but two are new (Extended Data Fig. 1 and Supplementary Table 1): one is on the X chromosome, which was not analyzed in previous genome-wide association studies (GWAS), and the other, rs334 in *HBB*, is a variant known to cause sickle cell disease, which is predominantly seen in individuals with African ancestry. As *HBB* was used as a control gene in our LTL assay, the fidelity of its apparent association with LTL was investigated in further analyses, which strongly suggested that this is an artifactual association (Supplementary Note and Supplementary Fig. 2). This locus was, therefore, removed from further analyses; we advise caution in the use of this control gene in future studies of LTL, especially those involving participants of African ancestry. Except for rs334, none of the other associations were driven by inclusion of individuals with non-European ancestry (Supplementary Table 1). We also investigated the extent to which the technical effect of rs334 explained the observed difference in LTL between participants of Black and White ethnicity (as defined by the UKB), which we have reported elsewhere^15^. Although, as expected, removal of carriers of the rs334 minor allele attenuated the difference in LTL between participants of Black and White ethnicity, the participants of Black ethnicity still had significantly longer LTL than the participants of White ethnicity (Supplementary Note).

**Fig. 2:**
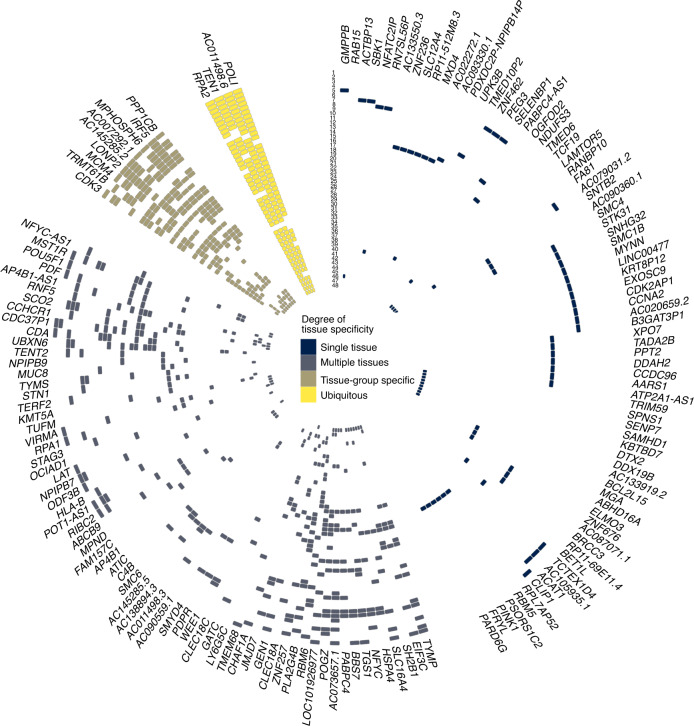
Identification of eQTL signals at genome-wide significant loci. Circular representation of colocalized eQTLs across 48 tissues in GTEx. Strong colocalization is shown as a colored tile, with the color determined by the degree of tissue specificity of colocalization: ubiquitous, ≥33 tissues; tissue-group specific, 17–32 tissues; multiple tissues, 2–16 tissues; and single tissue, one tissue. Tissues are represented numerically with full details in Supplementary Table 5. Genes are labeled using HGNC gene symbols. Tissues are ordered by GTEx tissue groupings, and genes are ordered by hierarchical clustering of the data, which groups genes with a similar colocalization pattern.

**Fig. 3:**
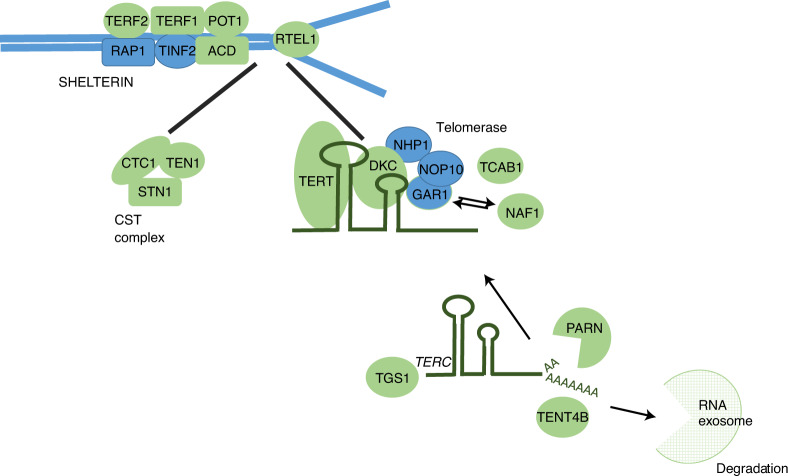
Genes with known regulatory roles in telomere maintenance located within GWAS loci. Key components of telomere regulatory complexes found within genome-wide significant loci. Proteins encoded within GWAS loci are depicted in green, and those not found within GWAS loci are depicted in blue. We found the majority of components of core telomere binding complexes alongside many proteins involved in the formation and activity of telomerase. Note that not all components of the RNA exosome are shown.

Combining information on gene function, variant annotations and colocalizing expression quantitative trait loci (eQTLs; Methods, Supplementary Note and Supplementary Tables 3–6), we were able to prioritize likely causal genes at 114 (83%) of the loci we discovered. Many biological candidates were supported by functional predictions and gene expression evidence, including strong eQTL support for *TEN1*, *STN1* and *RPA2* (Fig. 2 and Supplementary Tables 4–6). Genes with known roles in telomere regulation were found in 44 loci, including genes encoding components of the SHELTERIN (*ACD* (*TPP1*), *TERF1*, *TERF2* and *POT1*) and CST (*SNT1* (*OBFC1*), *TEN1* and *CTC1*) complexes, which act to cap the end of the telomere, suppressing inappropriate activation of the DNA-damage response and regulating telomerase processivity (Fig. 3)^18^. Components of the alternative lengthening of telomeres pathway (*ATRX*, *PML* and *SLX4*) were also among the new loci as well as genes encoding factors that post-translationally modify key telomere proteins, including *UPS7*, which encodes a protein that deubiquitinates both POT1 and ACD^19,20^. Genes within both known (*TERC*, *TERT* and *NAF1*) and new (*DKC1*, *TEP1*, *SMG6*, *SHQ1*, *NOLC1* and *RUVBL1*) loci encode core components of proteins that regulate the assembly and activity of telomerase (Supplementary Note)^21–24^. Before telomerase assembly, *TERC* undergoes complex processing^25^. Genes involved in *TERC* stability, intracellular trafficking and processing were found in known (*SMUG1*) and new (*PARN*, *TENT4B* (*PAPD5*), *TGS1* and *WRAP53*) loci, including those associated with the RNA exosome (*EXOSC6*, *EXOSC9*, *EXOSC10*, *DIS3* and *ZCCHC8*; Fig. 3 and Supplementary Note)^25–28^.

Other genes of interest in new loci are involved in DNA replication, recombination and repair, components of which have established roles in telomere maintenance^29^. Two new loci harbor components of the Replication protein A complex (*RPA1* and *RPA2*), which is recruited to telomeres during DNA replication^30^. The complex is later removed in a process involving hnRNPA1 (within the *SMUG1* locus) and replaced by POT1 (ref. ^31^). DNA double-strand-break repair genes with known roles in telomere regulation were also observed (*SLX4*, *MCM4* and *SAMHD1*)^32,33^. Two other genes highlighted as likely to be causal are *POLI* and *POLN*. Neither is known to have a direct role in telomere maintenance; however, other DNA polymerases that are involved in translesion repair function in the alternative lengthening of telomeres pathway^34^, suggesting plausible roles for these polymerases in controlling TL.

To provide more evidence for the candidacy of our prioritized genes, we investigated whether rare (<0.1% MAF) protein-altering variants in these genes were associated with LTL using gene-based tests (Supplementary Note). The aggregated scores for eight genes (*RTEL1*, *TERF1*, *TERT*, *ATM*, *PARN*, *SAMHD1*, *POT1* and *CTC1*) were significantly associated with LTL after Bonferroni correction (Supplementary Table 7). The directions of association with LTL for the individual variants included in this analysis are consistent with the known biological functions of these genes in telomere regulation (Supplementary Table 8 and Supplementary Note). For example, rare protein-truncating/altering variants throughout *RTEL1* were mostly associated with a shorter LTL, consistent with data suggesting that the full-length RTEL1 protein is required to facilitate telomere elongation by telomerase^35^.

To identify potentially new pathways responsible for TL regulation, we tested for over-representation of prioritized genes in known biological processes (Methods). As expected, the most significantly associated pathways identified were related to the regulation of telomere maintenance. Other enriched pathways, represented by multiple Gene Ontology biological processes, included box H/ACA snoRNP assembly and snoRNA 3′-end processing, highlighting key components of *TERC* regulation within the associated loci. Extending our previous identification of the relevance of nucleotide metabolism to LTL^3^, the current analysis more specifically prioritized pyrimidine metabolism through multiple associated Gene Ontology processes (Supplementary Table 9).

An additional motivation for undertaking the GWAS was to create genetic instruments to enable causal inference analysis of LTL with biomedical phenotypes. To minimize the inclusion of correlated variants or those showing extensive pleiotropy in these analyses, we filtered the 197 sentinel variants further (Methods), yielding 130 conditionally independent, uncorrelated and ‘non-pleiotropic’ genome-wide significant instruments used in the MR analyses described below (Supplementary Table 1).

### Influences on biomedical traits

**Fig. 4:**
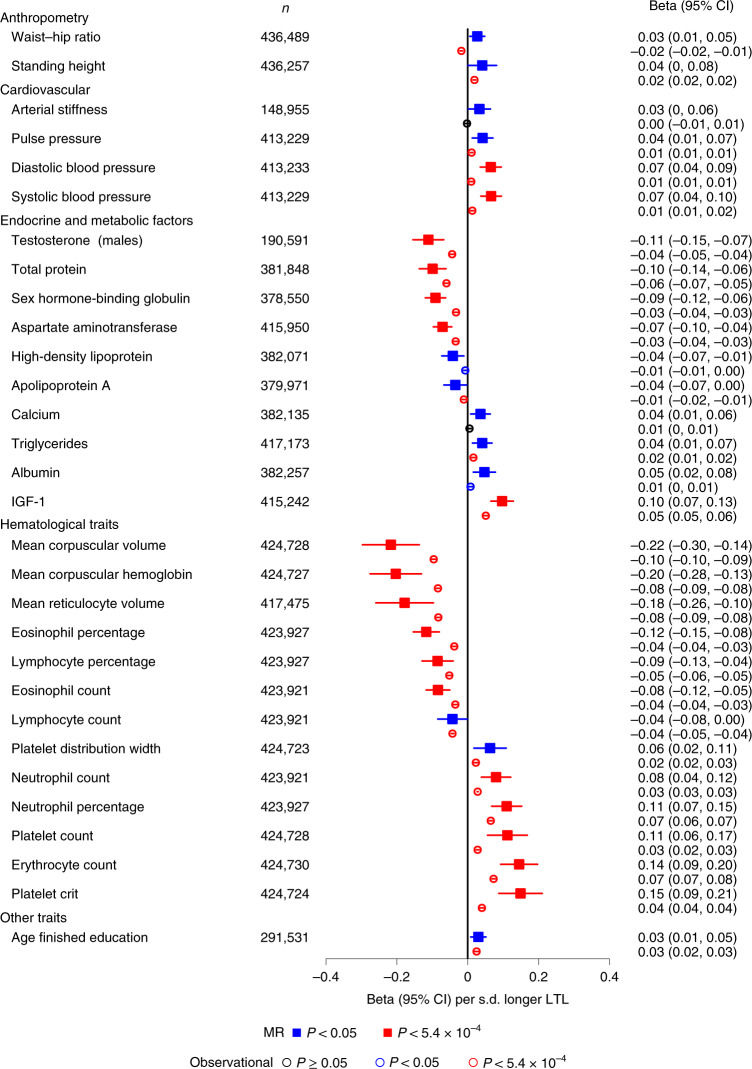
Biomedical traits associated with genetically determined LTL. Biomedical trait MR associations from the inverse-variance-weighted analysis (Supplementary Table 10) are shown with a solid square and expressed in beta per s.d. longer genetically determined LTL. Observational associations are shown with an empty circle and expressed in beta per s.d. longer usual LTL from linear regression models.

**Extended Data Fig. 2:**
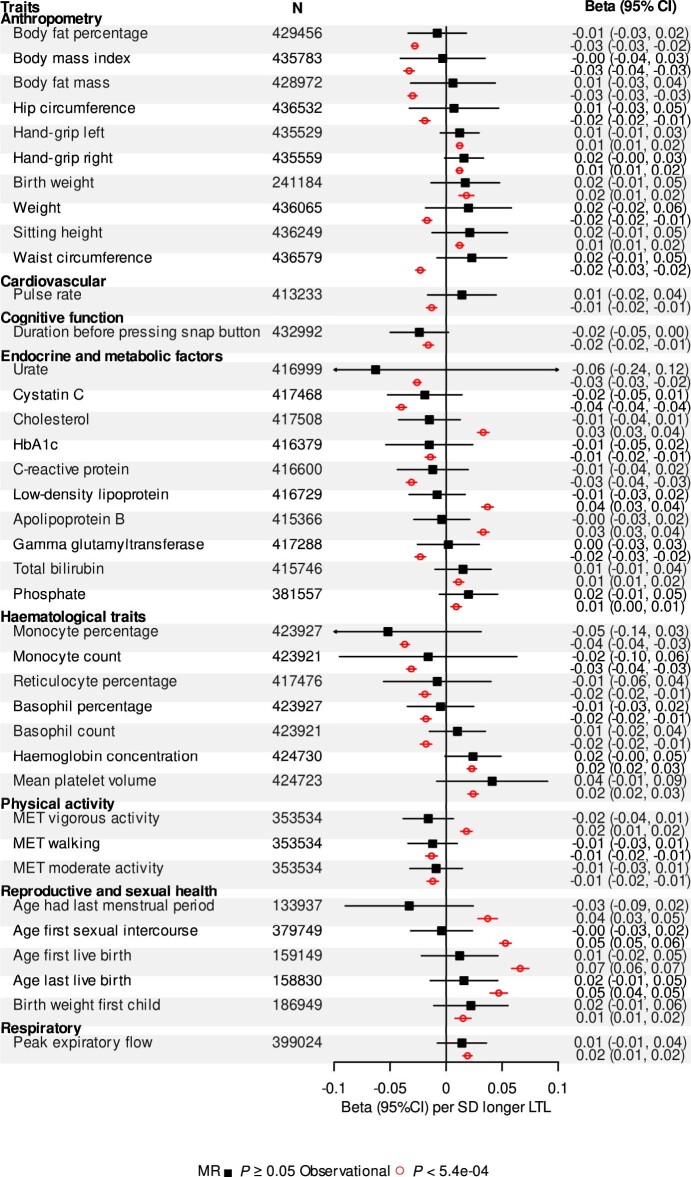
Biomedical traits associated with usual LTL only. Mendelian randomization (MR) associations are shown with a solid square and expressed as beta per standard deviation (s.d.) longer genetically determined leukocyte telomere length (LTL) for the inverse-variance weighted (IVW) analysis. Observational associations are shown with an empty circle and expressed in beta per s.d. longer usual LTL from linear regression models. CI, confidence interval. Full data for each trait can be found in Supplementary Table 10.

Partly guided by previous reports (Supplementary Table 10), we prioritized 93 biomedical traits available in the UKB, comparing MR results with observational results based on LTL levels corrected for the observed regression-dilution ratio of approximately 0.68 (abbreviated as ‘usual LTL’; Supplementary Note). We focused mainly on continuous traits related to body shape and size, cardiorespiratory function, reproductive health, physical fitness, bone marrow function, cognition, bone health, and liver and endocrine function (Supplementary Table 10). After Bonferroni correction, 18 of the traits were significantly associated in the same direction with both genetically determined LTL and usual LTL (Fig. 4 and Supplementary Table 10). Genetically determined LTL was more strongly related to most traits than usual LTL, probably reflecting lifelong influences (Supplementary Table 10). However, for all traits, LTL explained only a small proportion of the variance (<0.5%). For an additional 12 traits, we found nominally significant associations (*P* < 0.05) with genetically determined LTL, with most of these traits showing significant and concordant associations with usual LTL (Fig. 4 and Supplementary Table 10). A further 38 traits showed Bonferroni-significant observational associations but no associations with genetically determined LTL (Extended Data Fig. 2 and Supplementary Table 10). A lack of concordance for these traits could reflect either residual bias in observational analyses or limited statistical power in the MR analyses.

Overall, our findings demonstrate that variation in the LTL affects a wide range of biological and physiological traits spanning multiple body systems. We confirmed associations of genetically determined longer LTL with higher blood pressure^36^ and identified new associations with circulating biomarkers of metabolic and endocrine function, including higher insulin-like growth factor 1 (IGF-1) and lower sex hormone-binding globulin (Fig. 4 and Supplementary Table 10). IGF-1 is a growth hormone associated with pubertal growth in height. Adjusting for IGF-1 levels attenuated the association between height and longer usual LTL (beta = 0.011 (0.010, 0.013); *P* = 1.91 × 10^−27^), suggesting that IGF-1 may partly mediate the relationship between LTL and height. Notably, we observed associations between genetically determined LTL and multiple hematological traits (Fig. 4). Associations of longer LTL with higher counts of neutrophils, platelets and erythrocytes but lower counts of lymphocytes and eosinophils (Fig. 4 and Extended Data Fig. 2) suggest an effect of LTL variation on lineage fate at the lympho-erythromyeloid progenitor level^37^. The contrasting associations of LTL with erythrocyte count versus erythrocyte size and hemoglobin content may reflect a primary effect on the maintenance of red blood cell mass^38^. However, for platelets, longer LTL was associated not only with a higher count but also with larger volume, resulting in an increased platelet mass (Fig. 4 and Extended Data Fig. 2), consistent with recent observations that megakaryocytes—platelet precursor cells that reside in the bone marrow—originate directly from megakaryocyte-primed hematopoietic stem cells^39^ and not from a precursor cell clonally related to erythroid precursors.

### Influences on disease outcomes

**Fig. 5:**
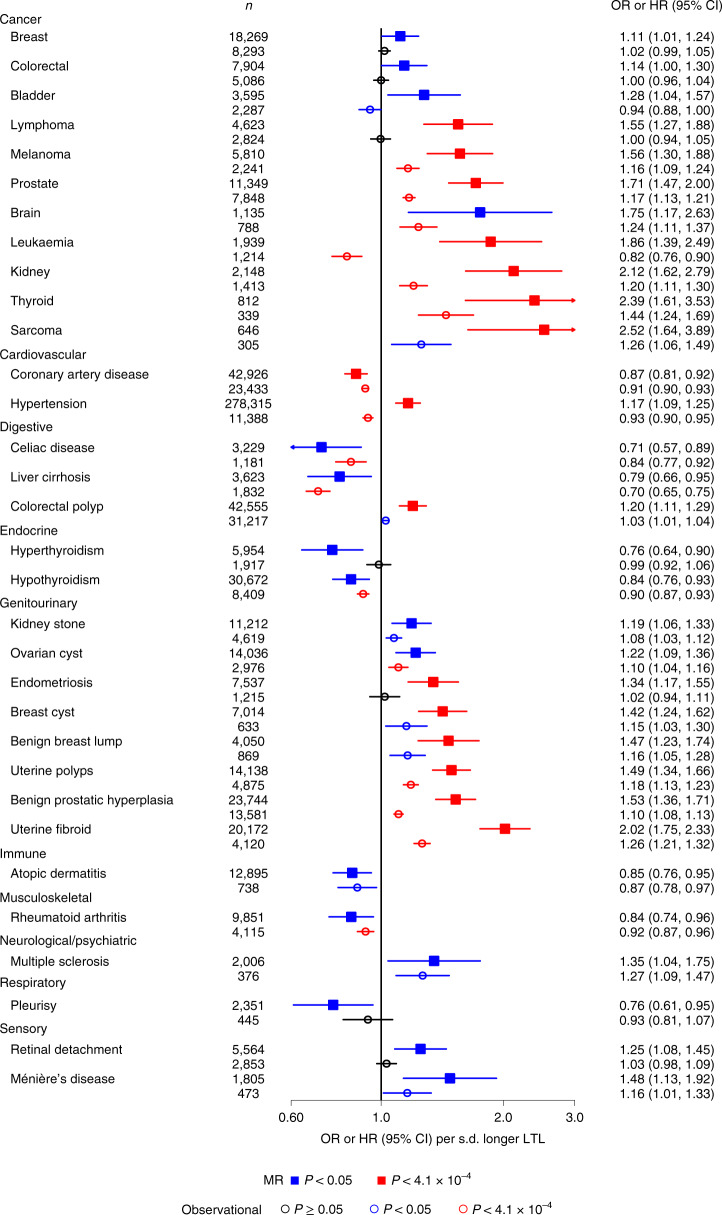
Diseases associated with genetically determined LTL. Disease MR associations from the inverse-variance-weighted analysis (Supplementary Table 12) are shown with a solid square and expressed as odds ratio (OR) per s.d. longer genetically determined LTL. Observational associations are shown with an empty circle and expressed as the hazard ratio (HR) per s.d. longer usual LTL from Cox proportional hazards models. *n* refers to the number of cases for each condition.

To identify causal links between LTL and disease outcomes using MR analyses, we prioritized 123 diseases defined using information available in the UKB (Supplementary Table 11 and Supplementary Note). We compared the results from the MR analyses with observational Cox regression analyses of incident cases. After Bonferroni correction, 16 of the diseases were significantly associated with genetically determined LTL (Fig. 5 and Supplementary Table 12). We confirmed associations of longer genetically determined LTL with lower risk of coronary artery disease as well as with a higher risk of several organ-specific cancers, including prostate, melanoma, thyroid and kidney, and genitourinary tumors (uterine polyps and fibroids)^3,11^. We found new associations of longer genetically determined LTL with a higher risk of sarcoma (a malignant tumor of connective or hematopoietic tissues) and endometriosis (the growth of endometrial tissue outside of the uterus). Results were consistent across sensitivity analyses, suggesting robustness to horizontal pleiotropy (Supplementary Figs. 3 and 4).

Of the 16 diseases that were significantly associated with genetically determined LTL, 12 were also Bonferroni-corrected or nominally (*P* < 0.05) significantly associated with usual LTL in the same direction (Fig. 5). For most conditions causally linked to LTL, we identified approximately log-linear dose–response relationships of usual LTL with incident outcomes (Supplementary Figs. 5 and 6). As for biomedical traits, we found that genetically determined LTL was more strongly related to diseases than usual LTL (Fig. 5). For two conditions (leukemia and hypertension), we observed significant results in opposing directions for the MR and observational analyses (Fig. 5). For leukemia, we observed a U-shaped association with usual LTL (Supplementary Fig. 5), which may represent different stages of the disease process within individuals before diagnosis. Hematopoietic stem cells with a longer TL are more likely to accrue somatic mutations that potentially lead to leukemic transformation^40^, whereas subsequent high proliferation rates during clonal expansion and the resulting telomere attrition are consistent with the shorter TL in tumors noted in other observational studies^41^. For hypertension, it was probably due to residual bias in the observational analysis (Supplementary Fig. 7, Supplementary Table 13 and Supplementary Note). We did not find evidence that blood pressure or plasma lipid levels (high-density lipoprotein cholesterol, low-density lipoprotein cholesterol and triglycerides) explained the association between shorter genetically determined LTL and a higher risk of coronary artery disease.

**Extended Data Fig. 3:**
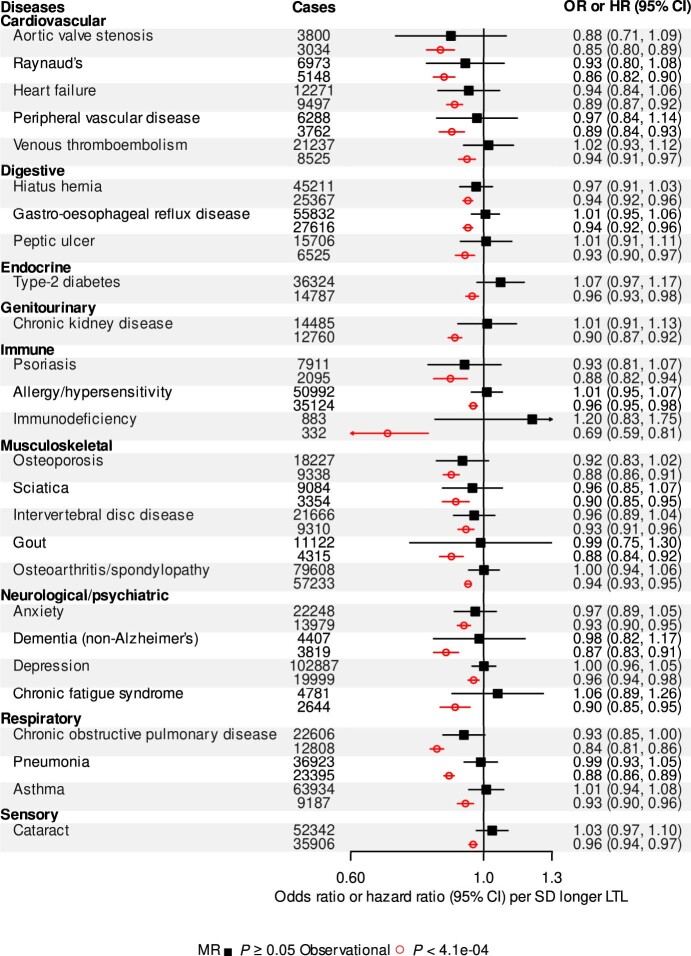
Diseases associated with usual LTL only. Mendelian randomization (MR) associations are shown with a solid square and expressed in odds ratio (OR) per standard deviation (s.d.) longer genetically determined leukocyte telomere length (LTL). Observational associations are shown with an empty circle and expressed in hazard ratio (HR) per s.d. longer usual LTL from Cox proportional hazards models. CI, confidence interval. Full data for each disease can be found in Supplementary Table 12.

For an additional 16 diseases, we found nominally significant (*P* < 0.05) associations with genetically determined LTL (Fig. 5). Of these, ten also had Bonferroni or nominally significant and concordant associations with usual LTL (Fig. 5), suggesting that future more powerful MR studies may strengthen the evidence for causality. These included new associations with colorectal cancer, liver cirrhosis, kidney stones and atopic dermatitis. For 26 diseases, we found Bonferroni-significant associations with usual LTL but non-significant associations with genetically determined LTL (Extended Data Fig. 3 and Supplementary Table 12). These findings could reflect either residual bias in the observational analysis or limited power in the MR analyses (Supplementary Note). Finally, for 65 diseases, we found no association in either the MR or observational analyses (Supplementary Table 12).

### Influences on life expectancy

**Fig. 6:**
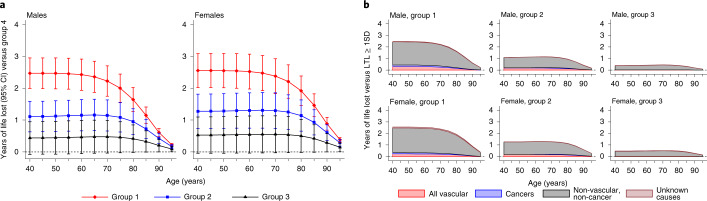
Years of life lost using UK 2015 mortality rates. **a**,**b**, The number of years of life lost were estimated by applying HRs for cause-specific mortality calculated from UKB data (specific to age-at-risk and stratified by sex) to population mortality rates for the United Kingdom during 2015 (by sex and 5-year age groups). Data are presented for four standardized LTL groups: group 1, >1 s.d. below the mean; group 2, ≤1 s.d. below the mean; group 3, <1 s.d. above or equal to the mean; and group 4, ≥1 s.d. above the mean) from 40 to 95 years of age. Group 4 was used as the reference group. Data are shown for males and females separately. This was performed for all-cause (**a**) and disease-specific (**b**) mortality. The UKB data included 458,309 participants and 28,345 deaths (comprising 5,984 vascular deaths, 14,916 cancer deaths, 7,244 non-vascular, non-cancer deaths and 201 deaths of unknown causes).

Given the causal links between LTL and multiple conditions—both in risk-increasing and risk-reducing directions—a relevant unresolved question is whether LTL has a net impact on life expectancy^42–44^. Using previously described public health modeling methods that draw on cause-specific mortality rates from the general population (Methods), we estimated that men with telomeres that were >1 s.d. shorter than the population mean at the age of 40 years had a lower life expectancy compared with those with telomeres that were ≥1 s.d. longer by 2.47 years (95% confidence interval (CI), 1.99–2.96; Fig. 6a); the corresponding estimates for women were very similar. These estimated differences were sustained to the age of 65 years and gradually declined thereafter. Excess cardiovascular deaths accounted for 13% and 9%, and cancer deaths 5% and 4%, of the survival differences in men and women, respectively, with most of the remainder due to other causes (Fig. 6b). Broadly similar results were observed in sensitivity analyses that involved different modeling assumptions (Supplementary Fig. 8).

## Discussion

This study elucidates the polygenic basis and biomedical consequences of LTL variation. In the most powerful genomic study so far, we implicate many new candidate genes, highlight the complex regulation of LTL and identify roles for *TERC* processing and pyrimidine metabolism. Using wide-angle analyses, we provide insight into the causal relevance of LTL to biological traits and diseases across multiple body systems, comparing genetic and observational associations in the same set of participants. There is much interest in shorter TL as a target for pharmacological and other interventions^45,46^. Two findings from our analyses provide insight into this issue. First, for coronary artery disease and most other conditions causally linked with a shorter LTL, we found continuous linear associations—that is, no threshold above which LTL stops being associated with risk—indicating that any benefits could accrue across the range of TL. On the other hand, the observation that a longer LTL is causally associated with risk of several cancers—possibly because longer telomeres allow more cell divisions and clonal expansion after first-hit cancer mutations, thereby increasing the likelihood of second-hit mutations that drive oncogenic transformation^47^—highlights the complexity of TL as a therapeutic target. In addition, our finding of a U-shaped relationship between usual LTL and leukemia potentially explains the dual character of the association observed between TL and cancers. The same mechanism may also exist for solid tumors, namely a longer TL predisposes individuals to an increased risk of cancer (as also supported by the MR analysis), but as tumor cells proliferate, cells within the tumor demonstrate a shorter TL^41^.

Our results suggest that, despite the directionally opposing associations of LTL with different diseases, a shorter LTL at the age of 40 years is on average associated with a decrease in life expectancy of approximately 2.5 years. For comparison, the estimated reductions in life expectancy from long-term cigarette smoking and having diabetes in midlife are about 10 and 6 years, respectively^48,49^. Overall, our study provides a major resource for understanding the relevance of LTL to many complex diseases and traits.

## Methods

### LTL measurements

The measurement of LTL in the UKB participants and the extensive quality checks and adjustment for technical factors are detailed elsewhere^15^. For the analyses presented in this paper, we included all participants with the LTL measured from a UKB baseline sample, where there was no mismatch in self-reported and genetic sex (*n* = 472,174; data-freeze, December 2020). The LTL values were log-transformed and *Z*-standardized for all analyses.

### GWAS

We used imputed genotypes available in the UKB^2^ for the GWAS. To ensure quality, we restricted the analysis to variants with a MAF of ≥0.1% (where imputation accuracy is greatest) and an INFO score of ≥0.3. We tested 19.4 million variants using the BOLT-LMM package, adjusting for age, sex, array and the first ten principal components (PCs). The analysis was run separately for chromosome 23, where males were coded as 0/2.

### Conditional association analyses

To identify independently associated variants within loci, we adopted a two-stage approach to conditional analyses. We first used the summary statistics for variants meeting a threshold of *P* < 1 × 10^−6^ from the GWAS to perform a joint conditional analysis using GCTA (version 1.25.2; see Supplementary Note). We set a genome-wide significance threshold at *P* < 8.31 × 10^−9^, which has been suggested as an appropriate threshold for GWAS studies incorporating lower-frequency and rare variants (MAF > 0.1%)^16^. All variants with *P* < 8.31 × 10^−9^ were then taken forward to stage two. In the second stage, we performed exact joint modeling using BOLT-LMM, where we adjusted for all other variants from stage one, age, sex, genotype array and the first ten PCs in the model. All variants emerging from this analysis with *P* < 8.31 × 10^−9^ were considered to be conditionally independent at the genome-wide significance level.

### Variance explained by the genetic variants

To estimate the variance explained by all conditionally independent genome-wide significant variants, we extracted them from the imputed genetic data, scored by allele dosage. We only included participants that had both autosome and X-chromosome data. To account for familial correlation, we randomly excluded one participant from each related pair, where a pair was related if the kinship coefficient (*K*) was >0.088, estimated using genetic relatedness^2^. A linear regression adjusted for age, sex, array and the first ten genetic PCs was run to estimate the model variance explained (*R*^2^). A second model including all genetic variants was then run to estimate the full model *R*^2^ with the difference in the model *R*^2^ used to determine the variance explained by the genetic variants.

To determine variants that passed an FDR^50^, we estimated the *P* value equivalent to a *q*-value of <0.01 as *FDR_P* < 3.9 × 10^−5^. All variants from the GWAS with *P* < 1 × 10^−4^ were tested using GCTA (Supplementary Note) to identify conditionally independent variants that passed our *FDR_P* threshold. These were then clumped using PLINK to include only independent variants not in linkage disequilibrium (*R*^2^ < 0.01). The remaining variants were then extracted and modeled as above to estimate the variance explained by the FDR set.

### SNP-based heritability

The SNP-based heritability was estimated from the GWAS summary statistics using the BLD-LDAK model implemented in the SumHer package using the precomputed tagging file for individuals from the UKB^51^.

### Identification of potential causal variants

To identify putative causal variants allowing for multiple putative causal variants within a locus, we performed a shotgun stochastic search using FINEMAP v1.4 (refs. ^52,53^). For each locus, we calculated the posterior probability of the causal configurations and report the most probable set. First, we defined a region to contain all variants within a 1 Mb window centered on each sentinel SNP. We identified the top causal variant for each region and identified all regions harbored within multiple sentinel GWAS loci. Initially, we specified there to be only one causal variant. We then grouped the regions in multi-lead-SNP GWAS loci by locus (containing *k* lead SNPs within the 1 Mb region). We then allowed for a maximum of *i* causal variants (*i* *=* *k* *+* *3*). If the maximum posterior probability (PP_icvar_) for having *i* causal variants in the region was ≤95%, we selected the causal configuration and then generated credible sets. If PP_icvar_ > 95%, we further allowed for a maximum of *j* causal variants (*j* *=* *i* *+* *3*) and selected the causal configuration that had the largest PP_icvar_ closest to 95%. However, if PP_icvar_ was very low, the single causal configuration was selected (Supplementary Table 14).

### Identification of potential causal genes

To identify potential causal genes within the associated loci, we identified genes with known roles in telomere regulation (candidate genes) and used information from variant annotation in the eQTL colocalization analyses. Functional annotation for all variants identified within the 95% credible sets produced from fine-mapping was collected using VEP^54^ (Supplementary Note).

To investigate whether the variants included within the 95% credible sets for each locus identified using FINEMAP shared a common causal variant with eQTL signals, we conducted colocalization analyses using COLOC^55^. Transcriptomic data were obtained from GTEx.v7 for genes with a *q*-value of <0.5 for all 48 tissues^56^. The COLOC method uses an approximate Bayes factor with both GWAS and eQTL summary statistics and regional linkage disequilibrium structure to estimate the posterior probabilities for five scenarios (PP0, PP1, PP2, PP3 and PP4). A high PP4 indicates evidence of a shared single causal variant. For each of the GWAS signals, we defined a 1 Mb region centered on the sentinel variant to test for colocalization using the COLOC R package (https://cran.r-project.org/web/packages/coloc/vignettes/vignette.html). We defined strong evidence of colocalization as PP3 + PP4 ≥ 0.99 and PP4/PP3 ≥ 5, and suggestive evidence as PP3 + PP4 ≥ 0.90 and PP4/PP3 ≥ 3, as previously described^4,57^.

Genes were prioritized on strength of evidence in the following order: biological candidate > high-impact annotation > moderate-impact annotation > strong evidence of colocalization > suggestive evidence of colocalization. Where expression of multiple genes was associated with our causal variants, we prioritized candidacy based on the number of tissues with evidence. To run downstream pathway analysis and gene-based tests where it was not possible to prioritize a gene at a locus, we substituted the nearest gene to the most significantly associated causal variant. Conversely, where it was not possible to prioritize a single gene from several with evidence, multiple genes were taken forward.

### Pathway analysis

We tested our list of prioritized or nearest genes for statistical over-representation (Fisher’s exact test) in PANTHER^58^. Pathways within the Gene Ontology biological process complete annotation set were considered to be significantly over-represented at an FDR *q*-value of <0.05.

### Gene-based tests

We removed noncoding RNAs, pseudogenes and poorly annotated new transcripts from the prioritized genes identified in the GWAS loci. We then extracted rare and ultra-rare variants (MAF < 0.1%) within the exon boundaries of these genes from the UKB exome sequencing data^59^. Protein-altering variants were scored as predicted high-confidence loss-of-function and ultra-rare missense variants based on annotation obtained from VEP using the VEP LOFTEE plugin (Supplementary Note)^54,60^. For each participant, the gene-specific score was obtained by aggregating the variant scores, capped at one (Supplementary Note). We tested the association between the gene-specific scores and LTL using linear regression implemented in R v.4.0.1, adjusting for age and sex. To support this, we ran single-variant analyses of the rare variants using PLINK v1.9, also adjusting for age and sex.

### Genetic instruments for MR analysis

Starting with the 193 sentinel variants located on the autosomes, we removed correlated variants from loci with more than one conditionally independent variant by removing those with *r*^2^ > 0.01 using PLINK clumping with linkage disequilibrium based on the same randomly selected UKB sample as for the conditional association analysis. We removed the *HBB* locus due to potential technical artifacts (Supplementary Note). To remove potentially pleiotropic loci, we investigated the remaining 147 variants for association with multiple traits and phenotypes using previously curated data^61^. For each variant, we derived the number of associations within different biological domains and defined evidence of pleiotropy as associations within at least three different domains. This led to the selection of 130 conditionally independent, uncorrelated and non-pleiotropic genome-wide significant instruments that we used for all MR analyses (Supplementary Table 1).

### Mendelian randomization

With our genetic instruments for LTL, we performed single-sample univariable MR using two-sample methods that have been shown to be robust in large-scale biobanks^62^. We used (1) the inverse-variance-weighted method for LTL based on all 130 independent and uncorrelated variants associated with LTL^63^, (2) MR-Egger regression to estimate unmeasured pleiotropy^64^, (3) weighted median estimator to assess the robustness to extreme SNP–outcome associations^65^ and (4) a contamination-mixture method to explore potential presence of multiple pathways^66^. To account for between-variant heterogeneity, we used multiplicative random-effects models in all analyses and quantified heterogeneity using the *I*-squared statistic from MendelianRandomization package v. 0.5.0 (https://CRAN.R-project.org/package=MendelianRandomization).

### Analysis of biomedical traits

To assess the influence of LTL on biomedical traits, we were partly guided by previous reports (Supplementary Table 12) in our prioritization of 93 biomedical traits, focusing only on continuous and binary outcomes. Continuous traits were first winsorized at the 0.5% and 99.5% percentile values to account for potentially influential outliers. After checking the distribution of the winsorized traits using histograms, natural logarithm transformations were applied to non-normally distributed traits where appropriate. All continuous biomedical traits were then scaled to the *Z*-standardized normal distribution. To account for familial correlation, we randomly excluded one from each related pair, where a pair was related if *K* > 0.088.

We used MR to investigate causal associations of LTL with biomedical traits. To estimate the genetic associations for each of our 130 genetic instruments with each biomedical outcome, we performed logistic regression for binary traits and linear regression for continuous traits, adjusting for age, sex, array and the first five genetic PCs using SNPTEST^67^. We then used MR to investigate causal associations of LTL with biomedical traits and ran MR sensitivity analyses (Supplementary Figs. 9 and 10).

Observational analyses were conducted to investigate the association between LTL and biomedical traits. The *Z*-standardized LTL was used as the predictor of interest to provide effect-size estimates for an increase in LTL of 1 s.d. Continuous traits were assessed using linear regression models, whereas logistic regression models were used for binary traits. All regression models were adjusted for age, sex, ethnic group (defined by the UKB as Asian, Black, Chinese, Mixed, Other and White) and white blood cell (WBC) count, as proposed elsewhere^15^. To correct for measurement error and within-person variability in LTL over time, observational associations of LTL with traits and diseases were corrected for the observed regression-dilution ratio of 0.68, as detailed elsewhere^15^. Observational associations relate to usual LTL, unless otherwise specified. The magnitude of association was estimated using a partial *R*^2^, calculated as the difference between the full model *R*^2^ and the model *R*^2^ leaving LTL out.

To assess nonlinear associations between LTL and the traits, a quadratic term (the squared value of the LTL) was included in separate models in addition to LTL, age, sex, ethnicity and WBC. We further assessed the nonlinear associations of LTL with various traits by fitting fractional polynomial models (Supplementary Figs. 11 and 12) adjusted for age, WBC count, sex and ethnic group. The best fitting fractional polynomial model, selected using *P* < 0.05 as evidence for selecting more complex nonlinear functions, was used to plot the continuous shape of association relative to the reference value of zero^68^. In further supplementary analyses, we calculated adjusted HRs by deciles of LTL and plotted them against the mean standardized LTL within deciles.

### Analysis of disease outcomes

We identified 123 diseases (Supplementary Table 8) using a slightly modified version of the strategy reported previously^4^ (Supplementary Note). The selection of diseases aimed to balance the needs for clinical relevance (for example, avoiding overlapping outcomes—that is, coronary artery disease and myocardial infarction), detail (to cover diseases with different physiopathology) and statistical power. We conducted power calculations due to the large differences in disease prevalence using the ‘powerLogisticCon’ function from the R package powerMediation^69^. These power calculations (Supplementary Fig. 13) showed that all outcomes had at least 60% power to detect an OR of 1.1 at the 5% level of significance. Around 75% of our disease outcomes, based on prevalence, had >99% power to detect an OR of 1.1, with 60% of our outcomes having >99% power to detect an OR of 1.05. To account for familial correlation, we randomly excluded one participant from each related pair, where a pair was related if *K* > 0.088.

Using a combination of prevalent and incident diseases (Supplementary Note), we estimated the genetic associations with each disease outcome using logistic regression. We then performed an MR using these estimates as for the biomedical traits described earlier. For the observational associations, time-to-event analyses were conducted between *Z*-standardized LTL and incident disease using Cox proportional hazards models, stratified by sex and ethnicity and adjusted for age and WBC count. For this analysis, participants with prevalent disease at baseline were excluded. To test the proportional hazards assumption, we fit an interaction term between LTL and time. For any deviations from proportional hazards (time interaction *P* < 0.05), we estimated the HRs at baseline and at 10 years via linear combination. We performed these analyses using the survival (https://CRAN.R-project.org/package=survival) and greg (https://CRAN.R-project.org/package=Greg) packages in R.

To investigate reasons for any discrepancies between the MR and observational results, we performed MR analyses using only incident disease outcomes and observational analyses using logistic regression with incident and prevalent data. The shapes of associations were assessed using fractional polynomials^70^ with Cox regression models adjusted for age and WBC and stratified by sex and ethnic group. The best fitting model was selected in the same way as for the biomedical trait analysis.

### LTL and longevity

Details of the methods used to estimate differences in life expectancy have been previously described^71^, with further specific information regarding the modeling for LTL provided in the Supplementary Note. Briefly, estimates of cumulative survival from the age of 40 years were calculated among four groups of *Z*-standardized measured LTL (group 1, >1 s.d. below the mean; group 2, ≤1 s.d. below the mean; group 3, <1 s.d. above or equal to the mean; and group 4, ≥1 s.d. above the mean, the reference group) by applying HRs for cause-specific mortality calculated from the UKB study (specific to age-at-risk and stratified by sex) to population mortality rates for the United Kingdom and European Union in 2015 (by sex and 5-year age groups). Calculations were performed giving specific consideration to interpreting estimated differences in life expectancy between groups 1 (that is, shorter telomeres) and 4 (that is, longer telomeres) from the age of 40 years. Analyses involved Stata version 14.0 (StataCorp) with two-sided *P* values and used a significance level of *P* < 0.05.

### Ethics

The UKB has ethical approval from the North West Centre for Research Ethics Committee (application 11/NW/0382), which covers the United Kingdom. The UKB obtained informed consent from all of the study participants. Full details can be found at https://www.ukbiobank.ac.uk/learn-more-about-uk-biobank/about-us/ethics. The generation and use of the data presented in this paper was approved by the UKB access committee under UKB application number 6077.

### Reporting Summary

Further information on research design is available in the Nature Research Reporting Summary linked to this article.

## Online content

Any methods, additional references, Nature Research reporting summaries, source data, extended data, supplementary information, acknowledgements, peer review information; details of author contributions and competing interests; and statements of data and code availability are available at 10.1038/s41588-021-00944-6.

## Supplementary information

Supplementary Information Supplementary Note and Figs. 1–20.

Reporting Summary

Supplementary Data 1 Regional association plots for GWAS sentinels. Plots are shown for each independent GWAS sentinel with a 1 Mb window.

Supplementary Tables Supplementary Tables 1–14.

Peer Review Information

## Data Availability

Source data are accessible via application to the UKB. Summary statistics of the GWAS are available at https://figshare.com/s/caa99dc0f76d62990195.
