# Supplementary material for: Polygenic basis and biomedical consequences of telomere length variation
Source: Nat Genet. 2021 Oct 5;53(10):1425–33. doi: 10.1038/s41588-021-00944-6 (PMC8492471; doi:10.1038/s41588-021-00944-6)
Supplement: Supplementary file 2 — Reporting Summary [file 41588_2021_944_MOESM2_ESM.pdf]

nature research

Corresponding author(s): Veryan Codd and Nilesh Smani

Last updated by author(s): Jul 26, 2021

Reporting Summary

Nature Research wishes to improve the reproducibility of the work that we publish. This form provides structure for consistency and transparency in reporting. For further information on Nature Research policies, see our Editorial Policies and the Editorial Policy Checklist.

Statistics

For all statistical analyses, confirm that the following items are present in the figure legend, table legend, main text, or Methods section.

n/a	Confirmed	
☐	☒	The exact sample size (*n*) for each experimental group/condition, given as a discrete number and unit of measurement
☐	☒	A statement on whether measurements were taken from distinct samples or whether the same sample was measured repeatedly
☐	☒	The statistical test(s) used AND whether they are one- or two-sided<br>*Only common tests should be described solely by name; describe more complex techniques in the Methods section.*
☐	☒	A description of all covariates tested
☐	☒	A description of any assumptions or corrections, such as tests of normality and adjustment for multiple comparisons
☐	☒	A full description of the statistical parameters including central tendency (e.g. means) or other basic estimates (e.g. regression coefficient) AND variation (e.g. standard deviation) or associated estimates of uncertainty (e.g. confidence intervals)
☐	☒	For null hypothesis testing, the test statistic (e.g. *F*, *t*, *r*) with confidence intervals, effect sizes, degrees of freedom and *P* value noted<br>*Give P values as exact values whenever suitable.*
☒	☐	For Bayesian analysis, information on the choice of priors and Markov chain Monte Carlo settings
☐	☒	For hierarchical and complex designs, identification of the appropriate level for tests and full reporting of outcomes
☒	☐	Estimates of effect sizes (e.g. Cohen's *d*, Pearson's *r*), indicating how they were calculated

Our web collection on statistics for biologists contains articles on many of the points above.

Software and code

Policy information about availability of computer code

Data collection	no software used
Data analysis	BOLT-LMM (c2.3.4), GCTA (v1.25.2), PLINK (v1.9), SumHer, FINEMAP (v1.4), VEP, COLOC, PANTHER (v16.0), SNPTEST and both R (v4.0.1) and Stata (v14.0 and v16.0) were used.

For manuscripts utilizing custom algorithms or software that are central to the research but not yet described in published literature, software must be made available to editors and reviewers. We strongly encourage code deposition in a community repository (e.g. GitHub). See the Nature Research guidelines for submitting code & software for further information.

Data

Policy information about availability of data

All manuscripts must include a data availability statement. This statement should provide the following information, where applicable:

- Accession codes, unique identifiers, or web links for publicly available datasets
- A list of figures that have associated raw data
- A description of any restrictions on data availability

Source data is accessible via application to UK Biobank. Summary statistics of the GWAS are available at https://figshare.com/s/caa99dc0f76d62990195

Field-specific reporting

Please select the one below that is the best fit for your research. If you are not sure, read the appropriate sections before making your selection.

☒ Life sciences ☐ Behavioural & social sciences ☐ Ecological, evolutionary & environmental sciences

For a reference copy of the document with all sections, see nature.com/documents/nr-reporting-summary-flat.pdf

Life sciences study design

All studies must disclose on these points even when the disclosure is negative.

Sample size	We used all participants within UK Biobank for whom we had a LTL measurement from a baseline sample, i.e. we could link phenotypic information to the same timepoint as the LTL measurement.
Data exclusions	We excluded non-baseline samples as the phenotypic data was not assessed at the same time point. We also removed individuals where self-reported sex and genetic sex did not match as this highlights potential sample handling issues and potential mismatches in sample identification. The sex mismatch data is provided by UKB.
Replication	Data are presented for the entire UK Biobank cohort for which we have measured leukocyte telomere length. We have not performed replication of the findings. To provide reassurance into the quality of the data we have reproduced previously reported findings from the literature as a measure of consistency between our data and previous, much smaller, studies.
Randomization	No randomization was performed. Genetic data were adjusted for age, sex, array and 10 genetic principle components. Disease and trait analyses were fit using regression models adjusted for age, sex, ethnicity and white cell count.
Blinding	Not applicable; for the analyses presented here there was no experimental design. Data are non-identifiable and the analyses were performed under hypothesis free approaches and principles. This includes the GWAS, disease screen and biological trait screen.

Reporting for specific materials, systems and methods

We require information from authors about some types of materials, experimental systems and methods used in many studies. Here, indicate whether each material, system or method listed is relevant to your study. If you are not sure if a list item applies to your research, read the appropriate section before selecting a response.

Materials & experimental systems

n/a	Involved in the study	
☒	☐	Antibodies
☒	☐	Eukaryotic cell lines
☒	☐	Palaeontology and archaeology
☒	☐	Animals and other organisms
☐	☒	Human research participants
☒	☐	Clinical data
☒	☐	Dual use research of concern

Methods

n/a	Involved in the study	
☒	☐	ChIP-seq
☒	☐	Flow cytometry
☒	☐	MRI-based neuroimaging

Human research participants

Policy information about studies involving human research participants

Population characteristics	This study has been performed using UK Biobank. All relevant information can be found at https://www.ukbiobank.ac.uk/
Recruitment	UK Biobank (UKB) is a large population cohort established between 2006 and 2010 of participants aged 40-69 years at recruitment (Sudlow, C. et al., PloS Med. 2015). Despite a relatively low response rate to invitations to participate (~6%) and some evidence of selection bias towards healthy, female, older, less socially deprived volunteers (Fry et al., Am J Epidemiol, 2017) it has been shown that associations in UK Biobank tend to be generalizable (Batty GD et al. BMJ, 2020). As we have measured LTL and utilized this data for the entire UK Biobank cohort we feel that no further bias has been introduced. In other work (doi:https://doi.org/10.1101/2021.03.18.21253457) we have shown that the relationships between age and sex with LTL are entirely within line of previous LTL analyses in other populations and have reproduced results from previous, smaller GWAS studies. We therefore feel that the results presented are not influenced by any selection bias in UK Biobank.
Ethics oversight	The UK Biobank has ethical approval from the North West Centre for Research Ethics Committee (Application 11/NW/0382), which covers the UK. UK Biobank obtained informed consent from all participants. Full details can be found at https://www.ukbiobank.ac.uk/learn-more-about-uk-biobank/about-us/ethics. The generation and use of the data presented in this paper was approved by the UK Biobank access committee under UK Biobank application number 6077.

Note that full information on the approval of the study protocol must also be provided in the manuscript.
