# Supplementary figures and images for: Polygenic basis and biomedical consequences of telomere length variation
Source: Nat Genet. 2021 Oct 5;53(10):1425–33. doi: 10.1038/s41588-021-00944-6 (PMC8492471; doi:10.1038/s41588-021-00944-6)

# Regional association plots for GWAS sentinels.

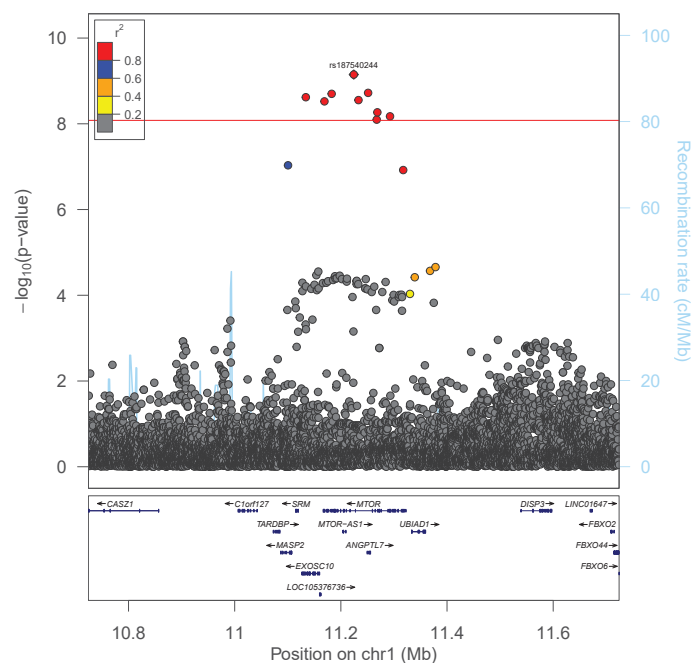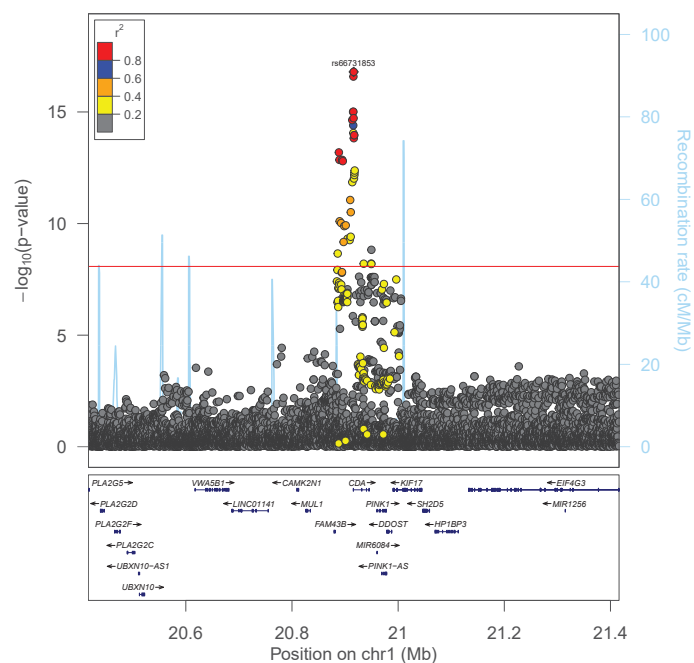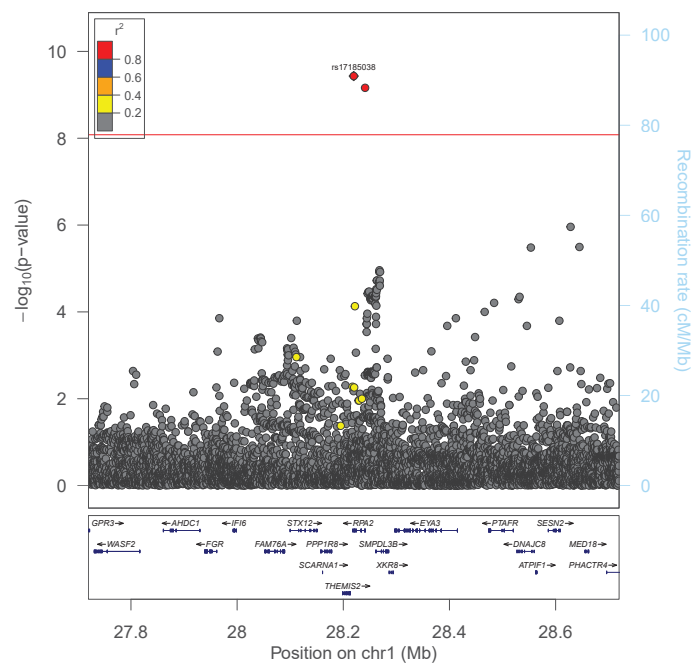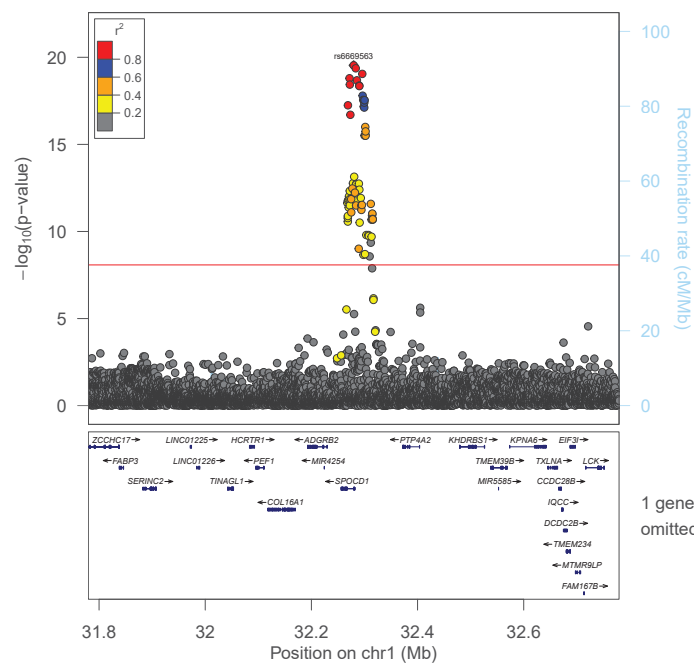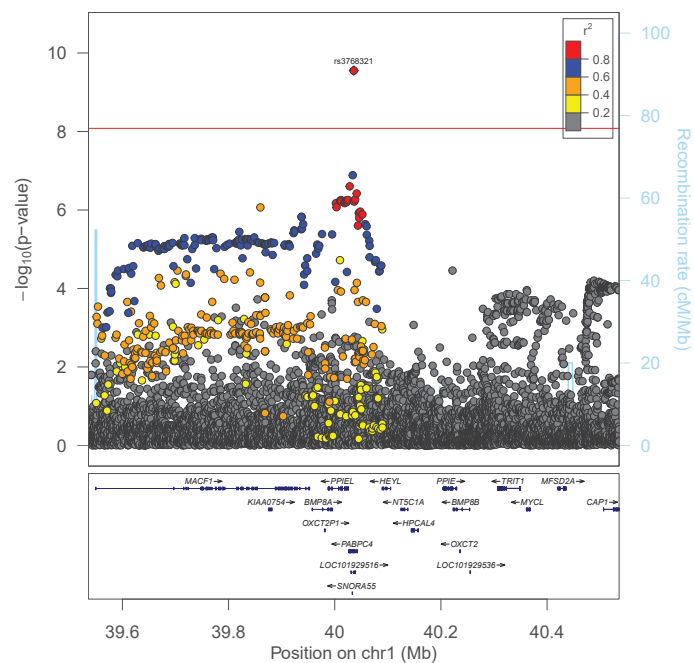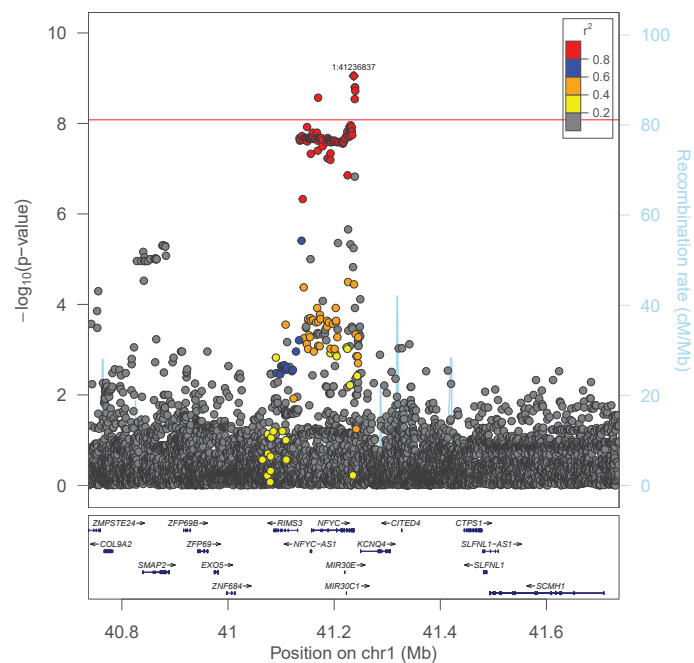

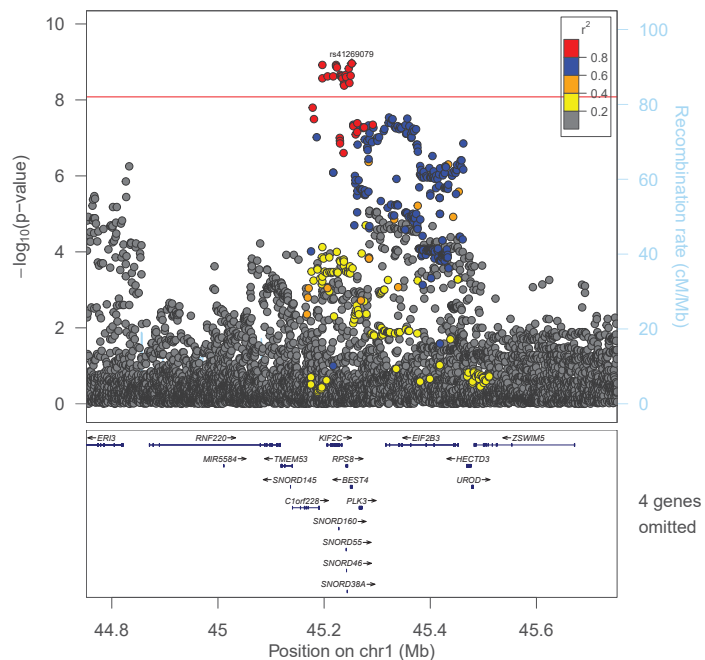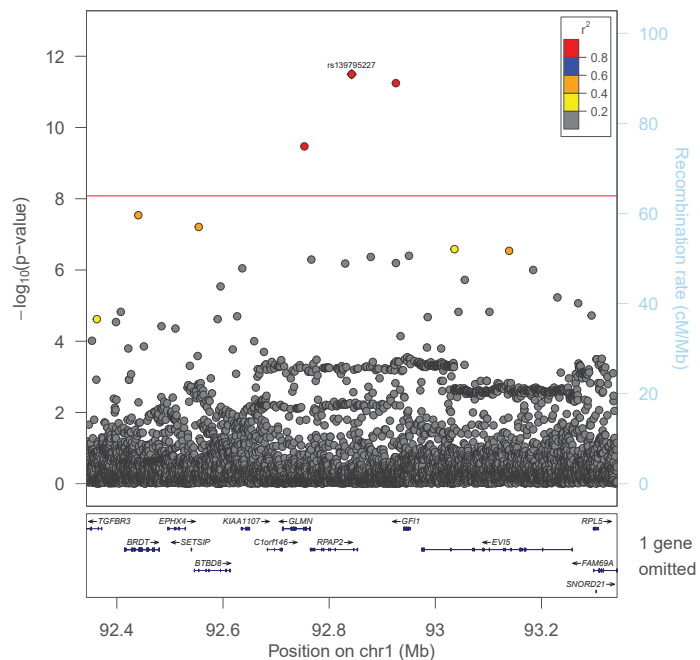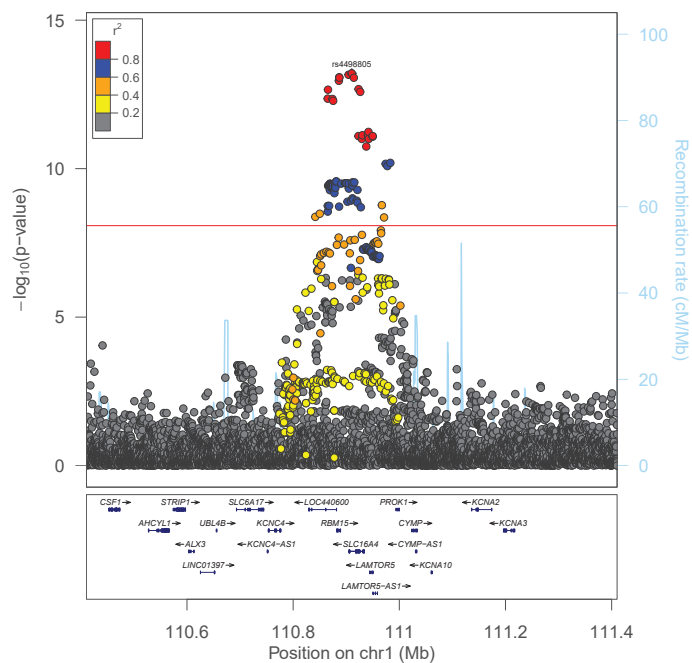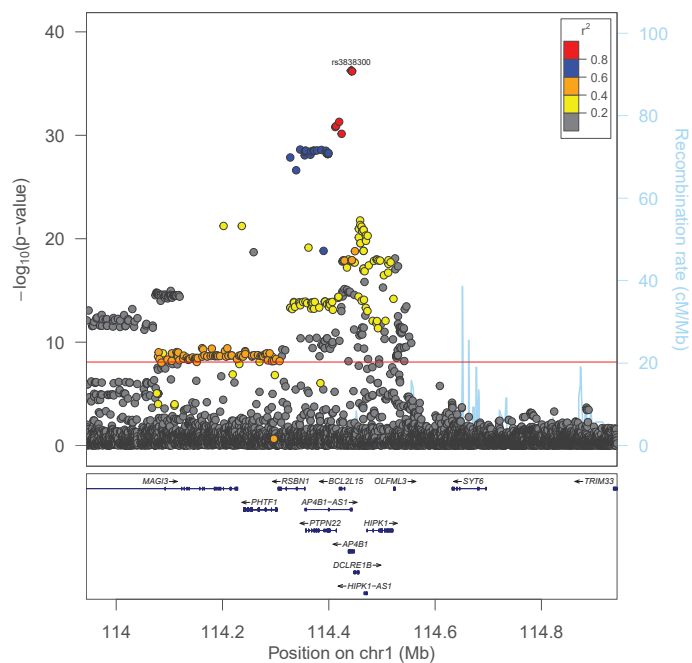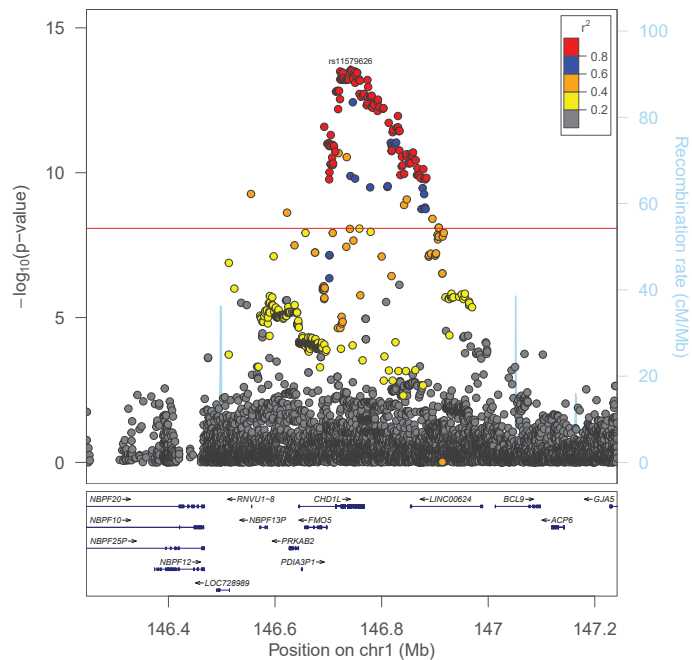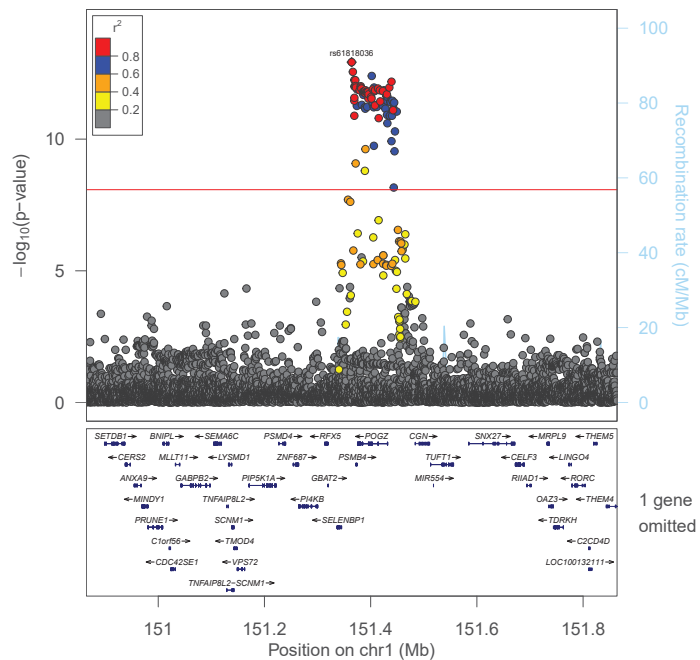

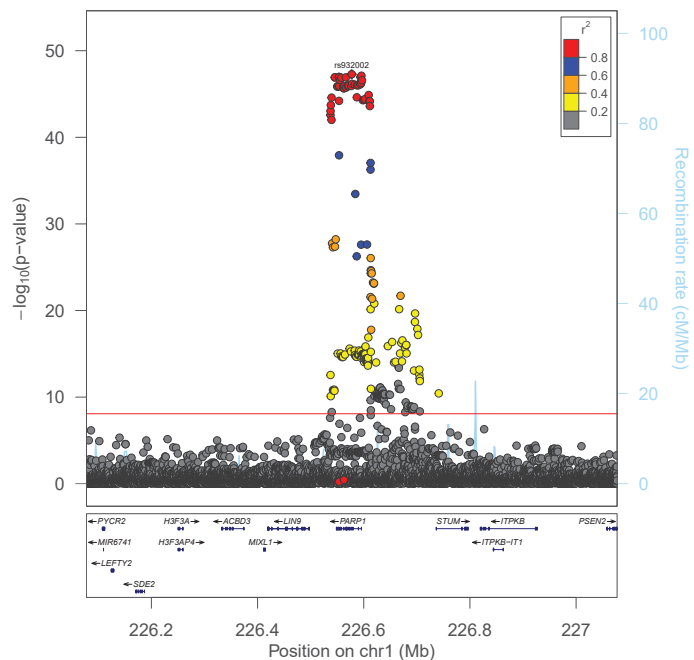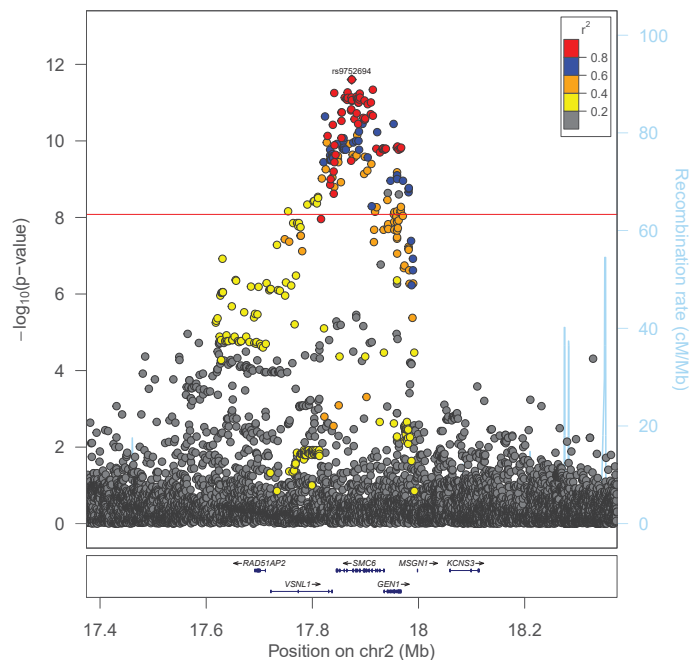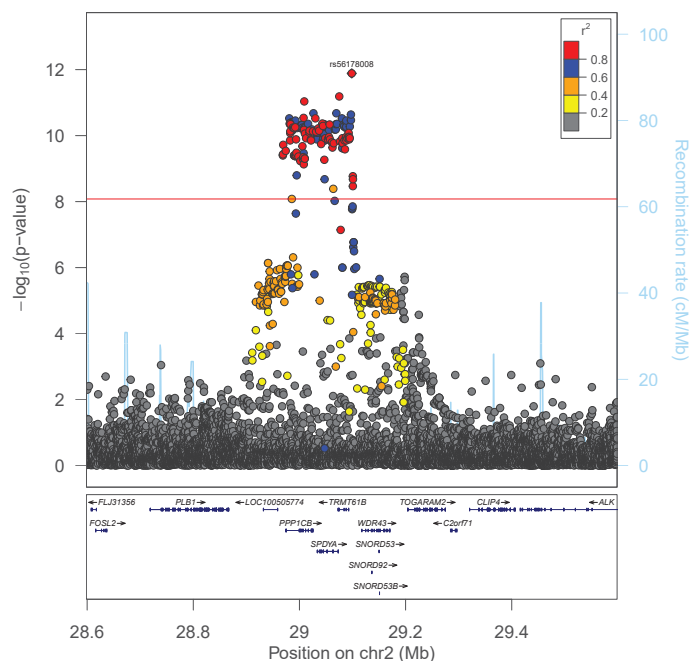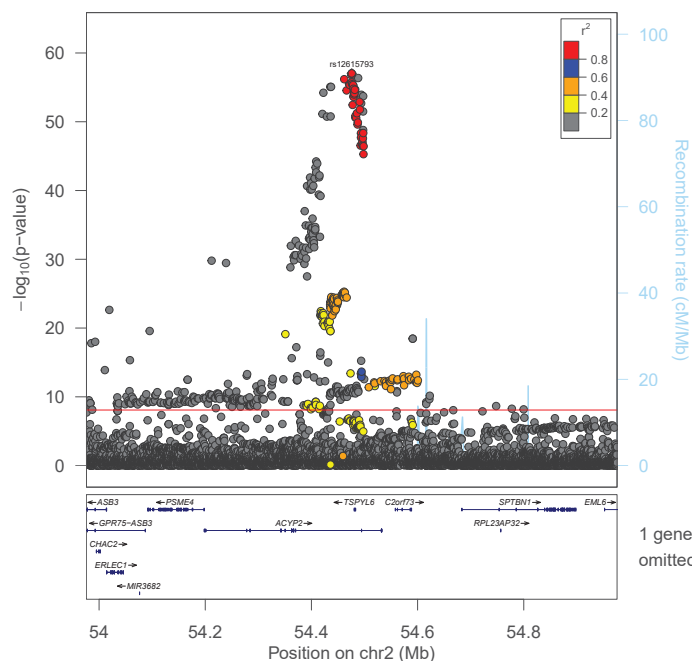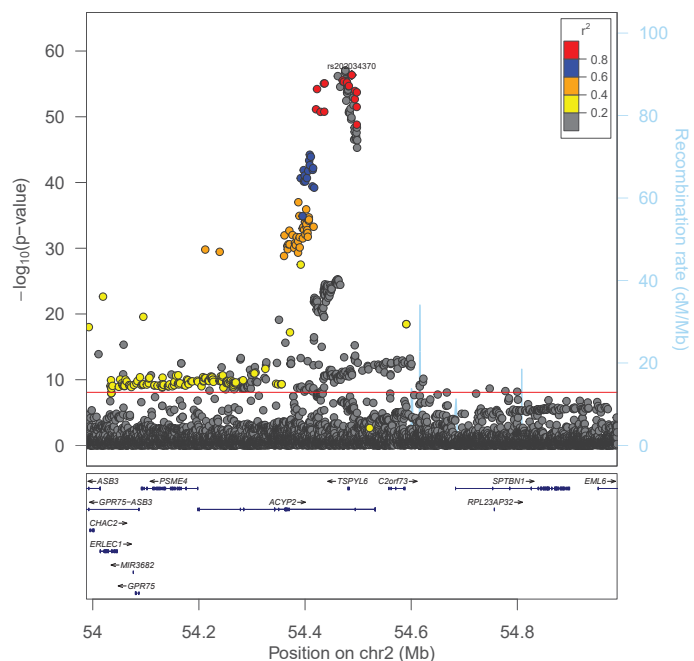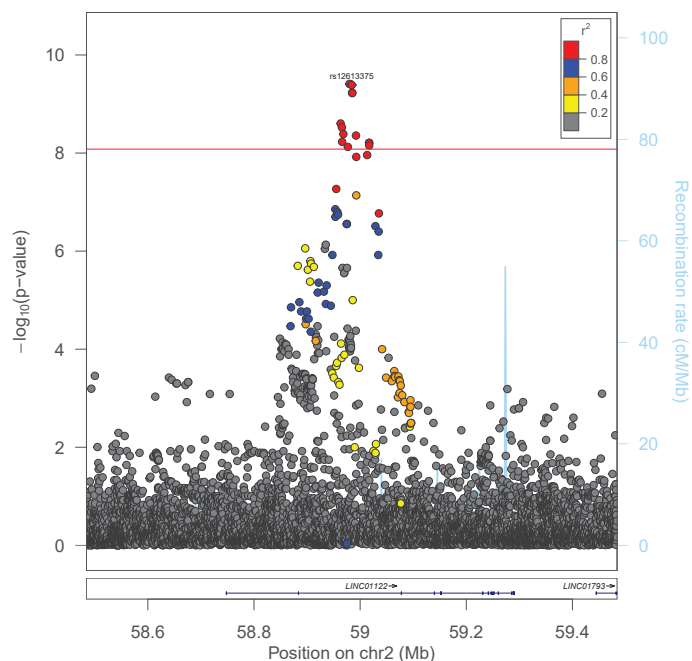

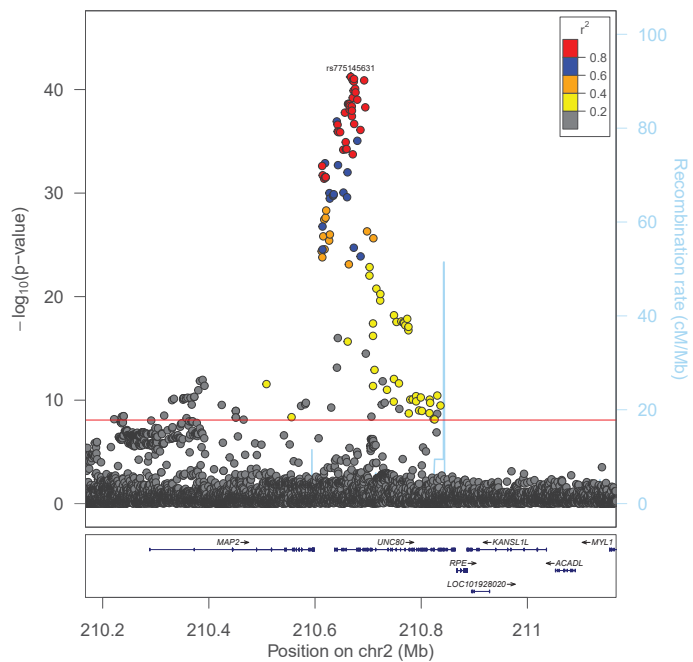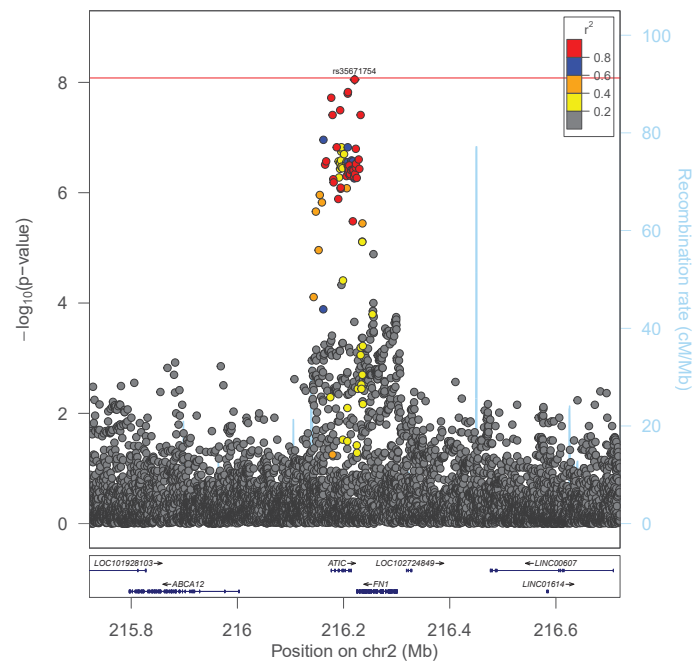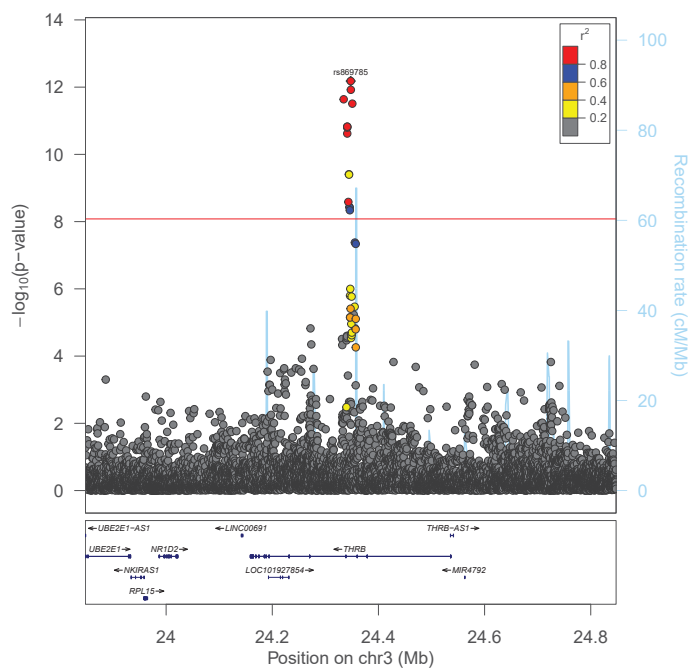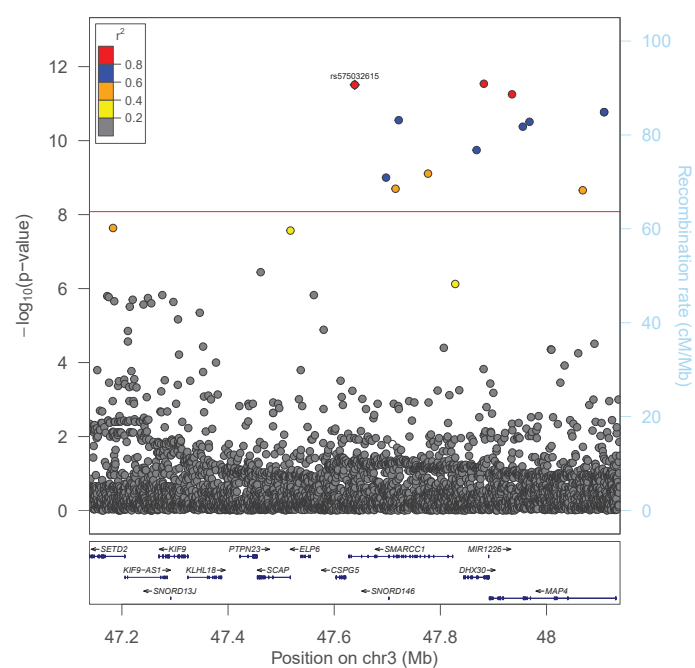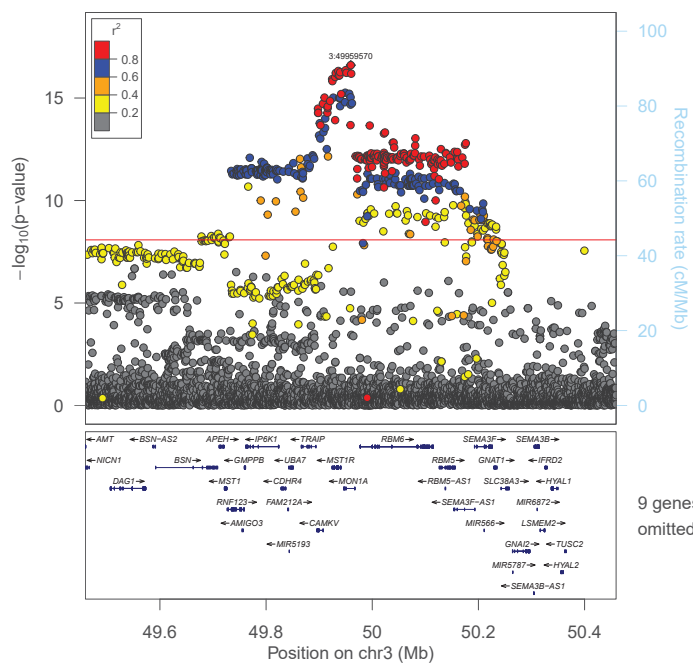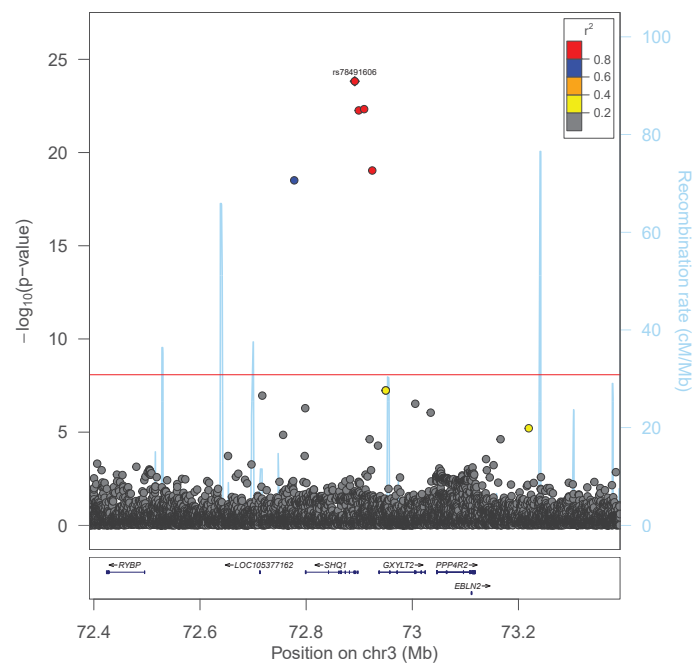

9 genes omitted

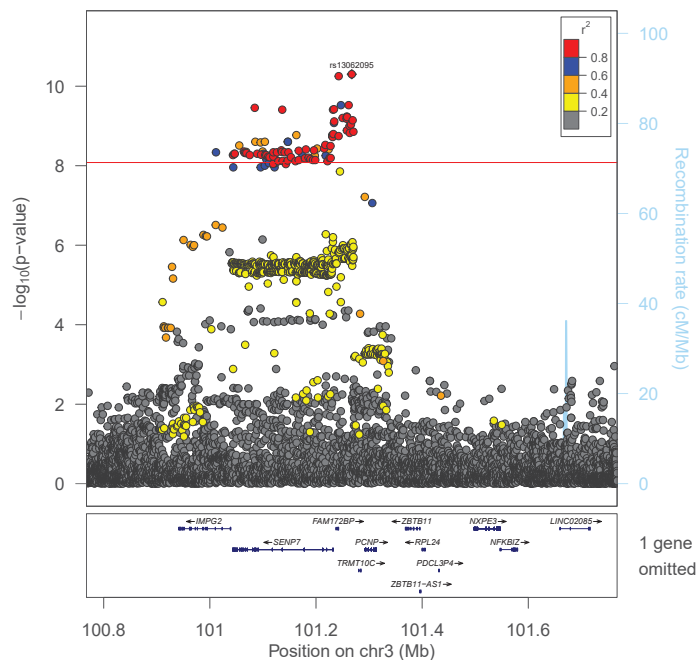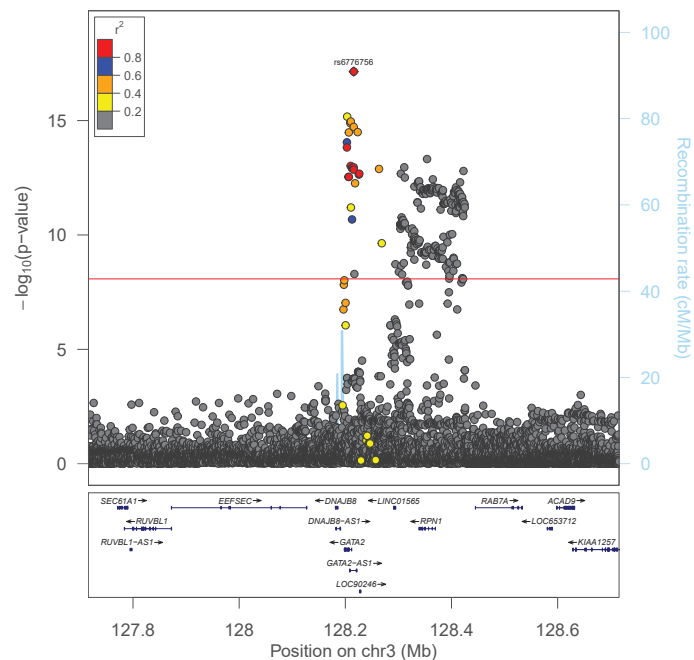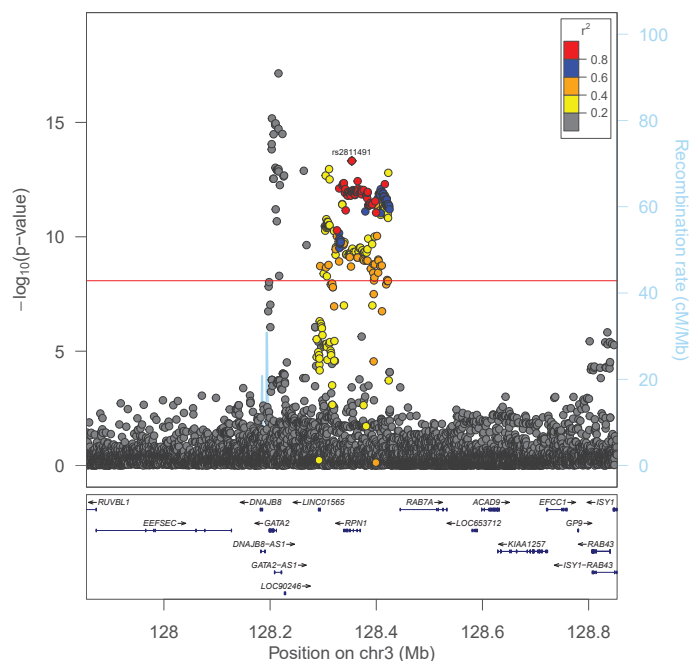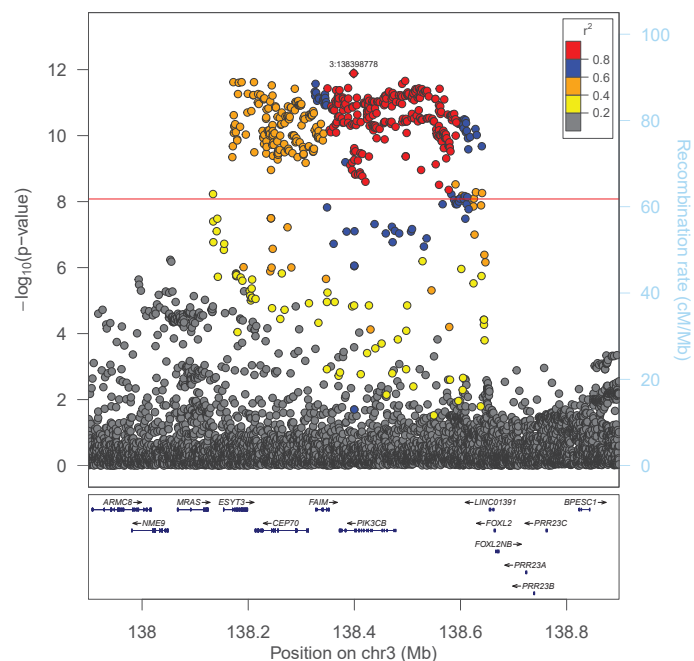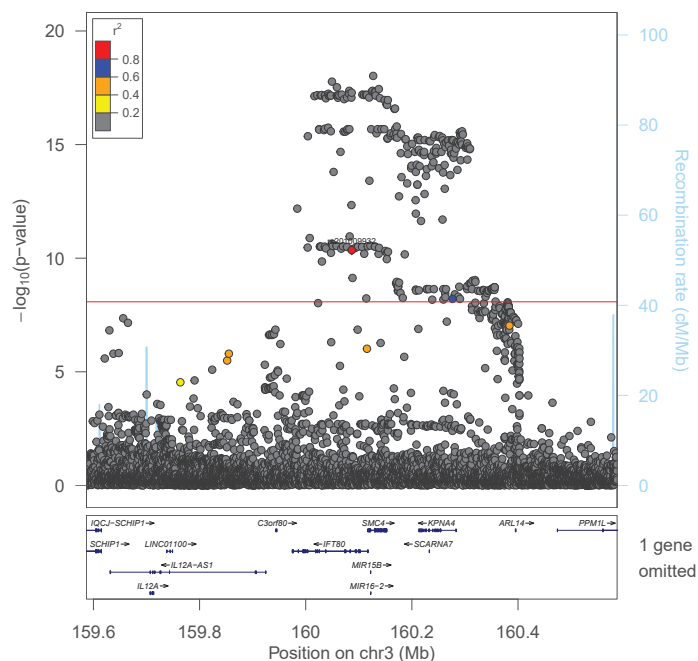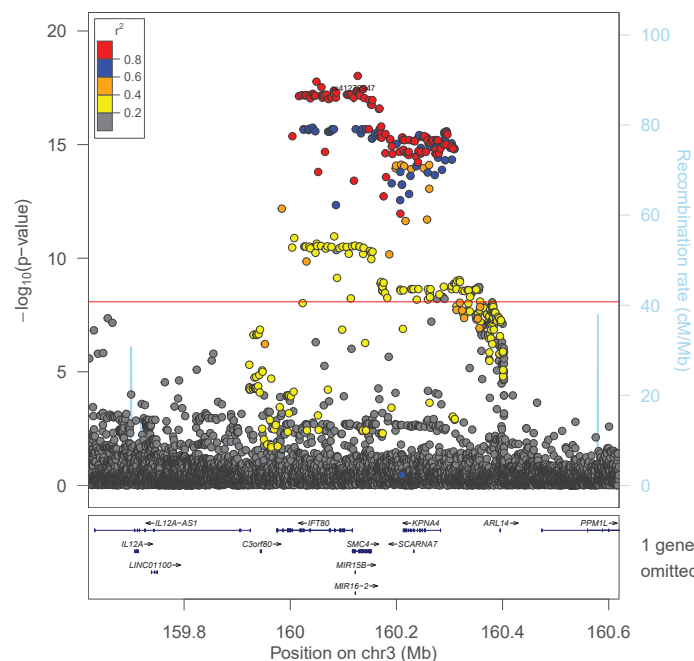

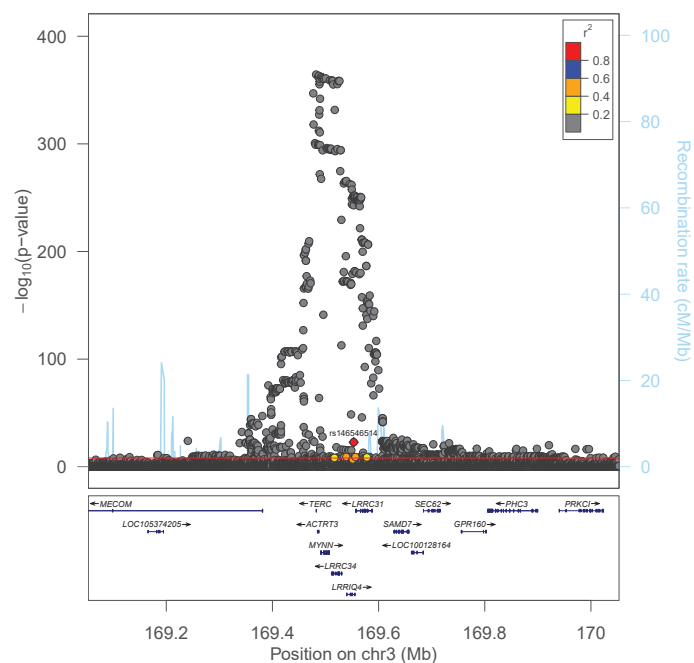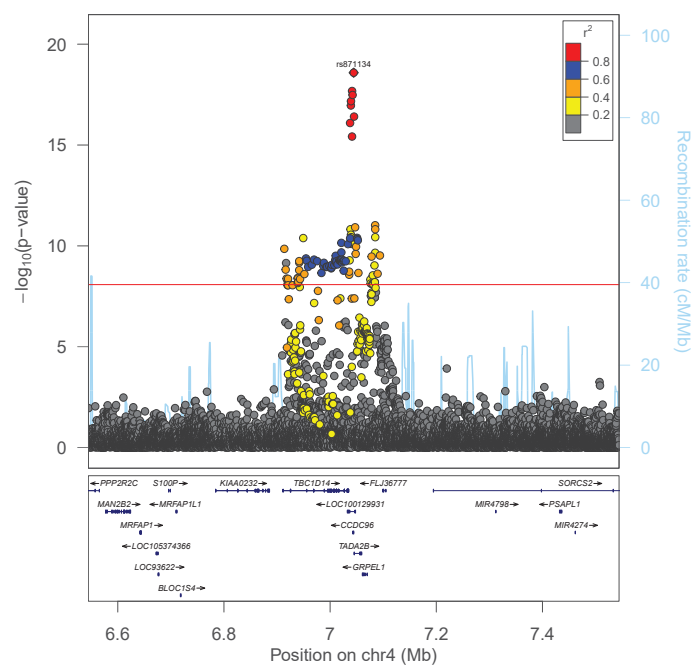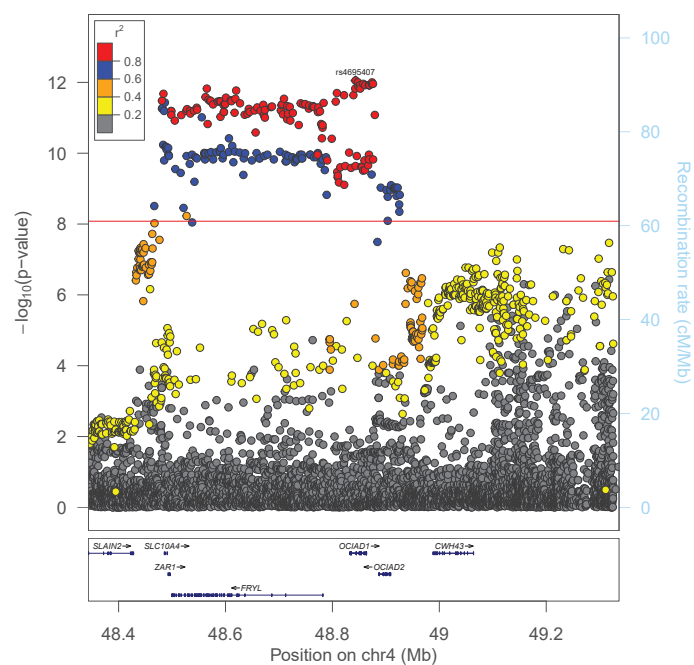

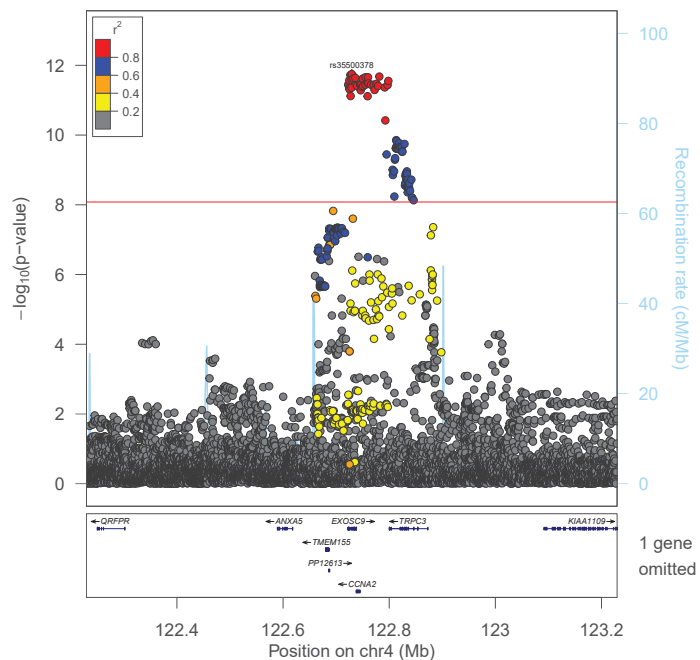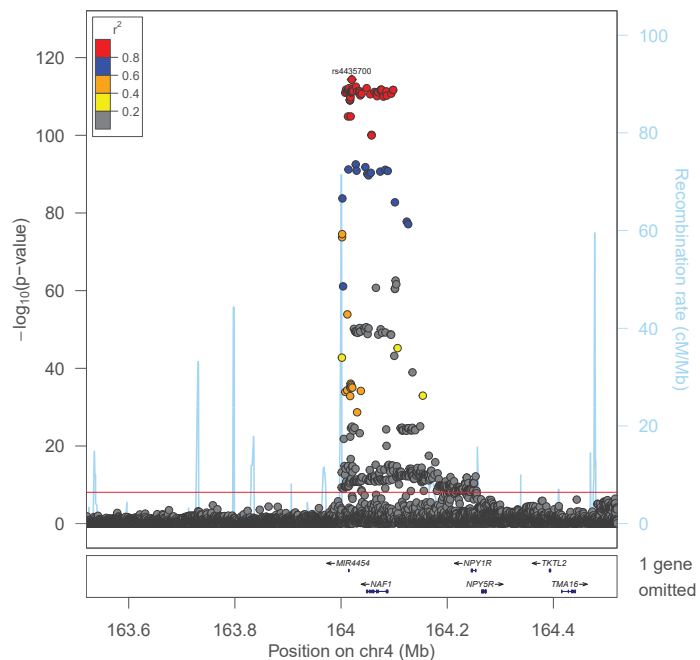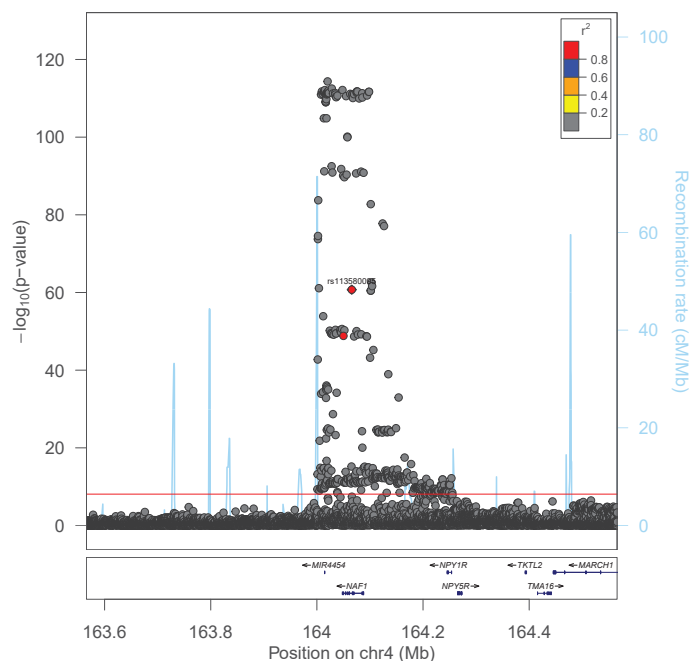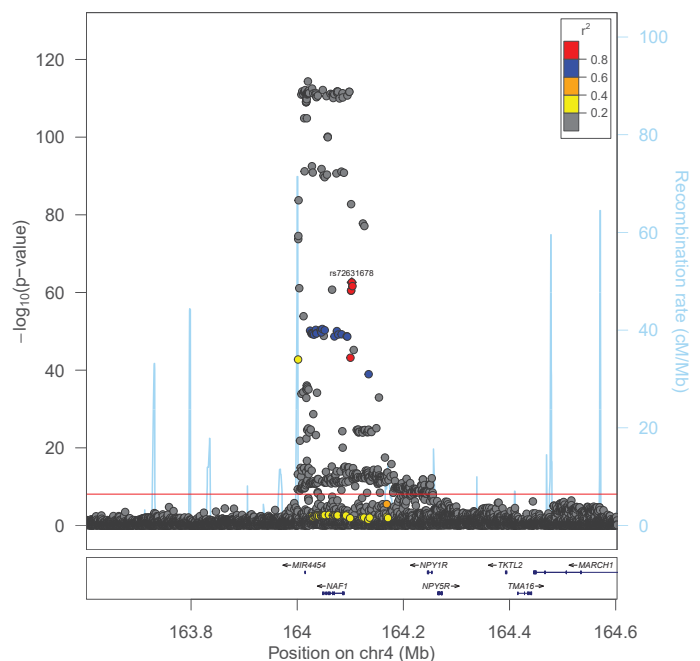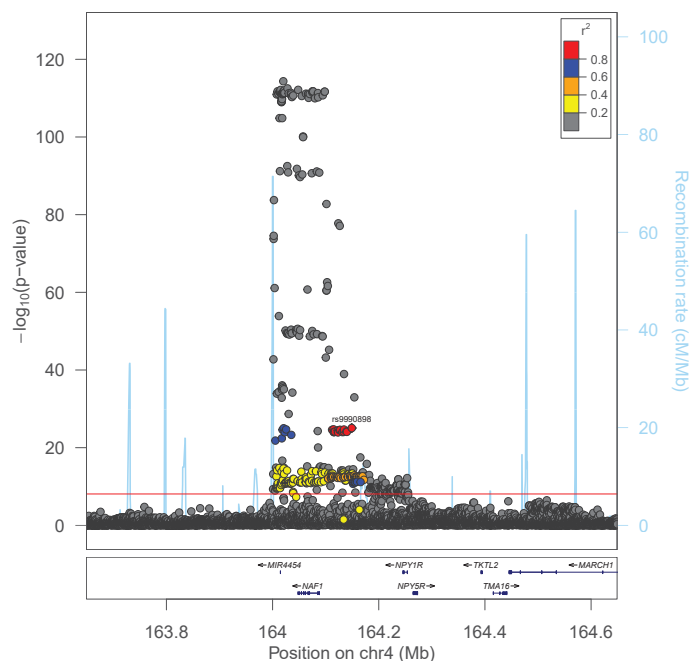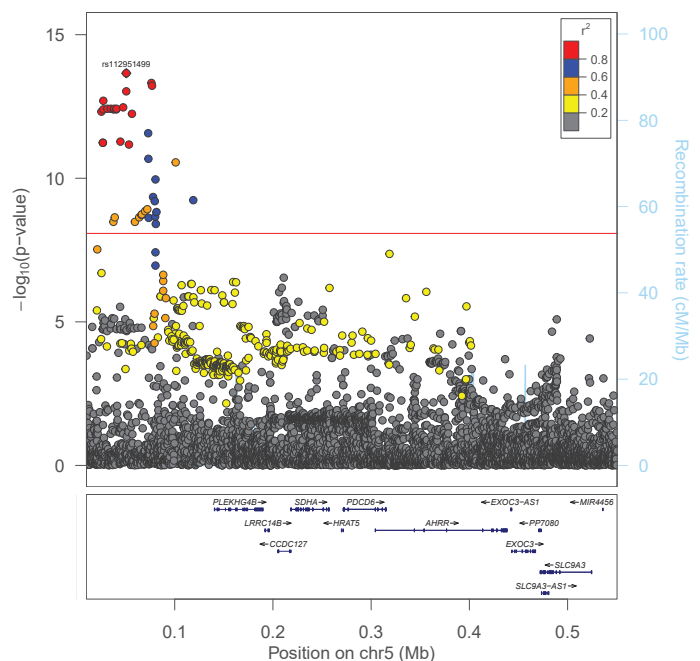

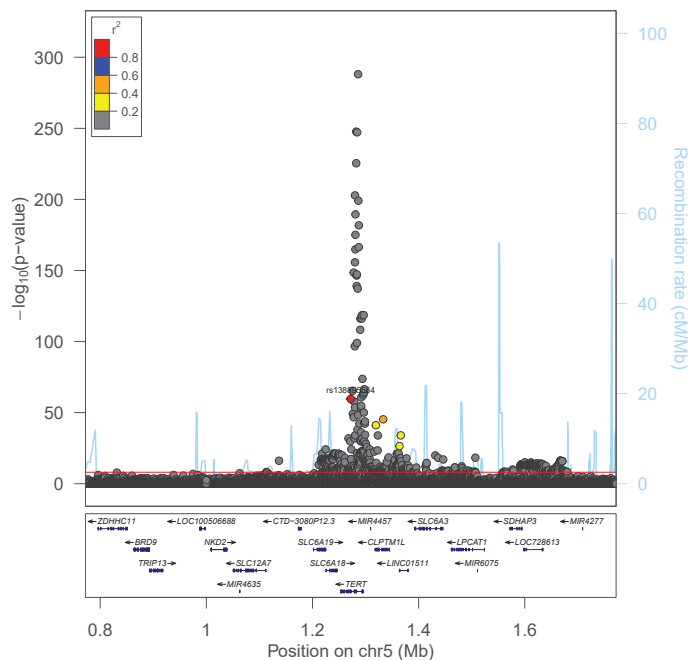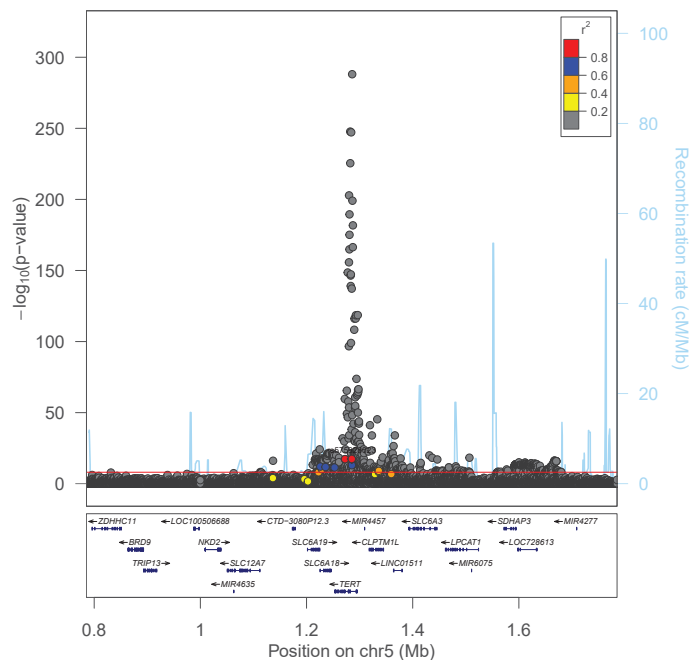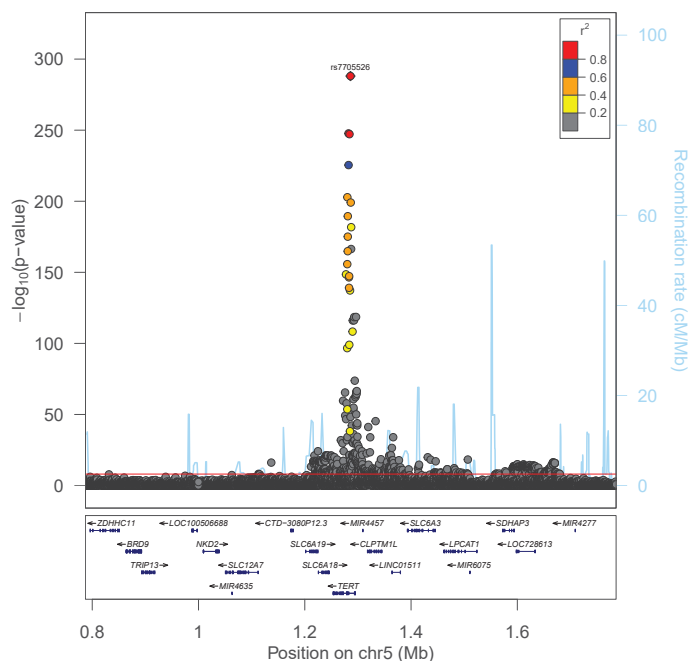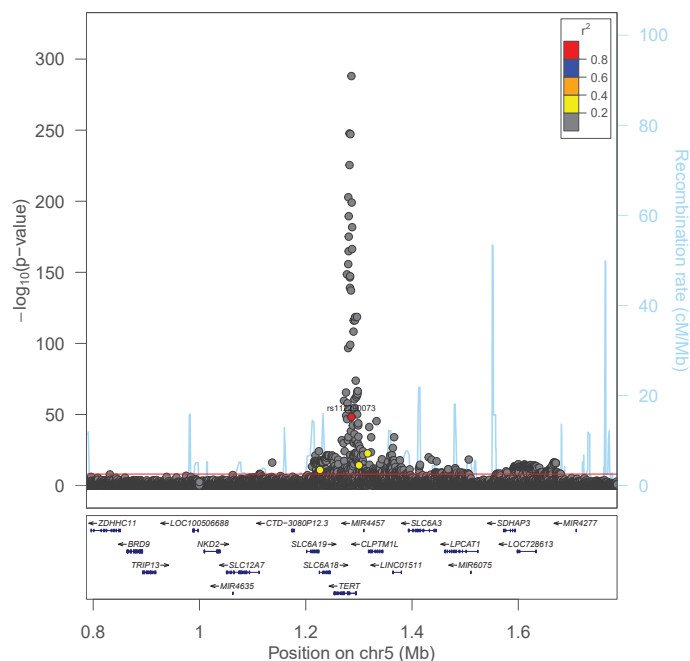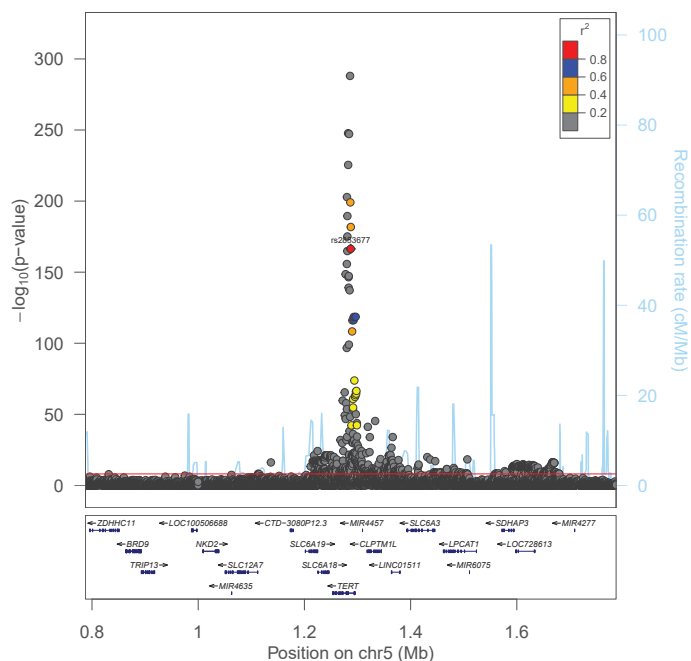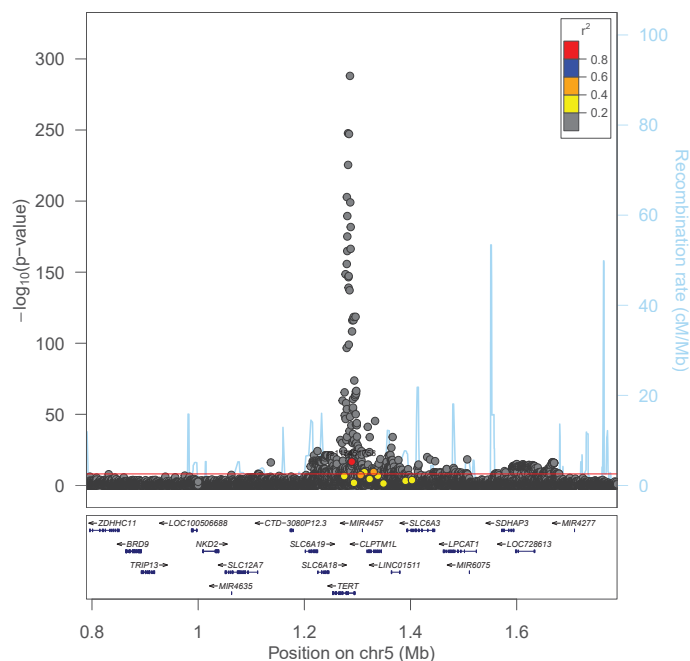

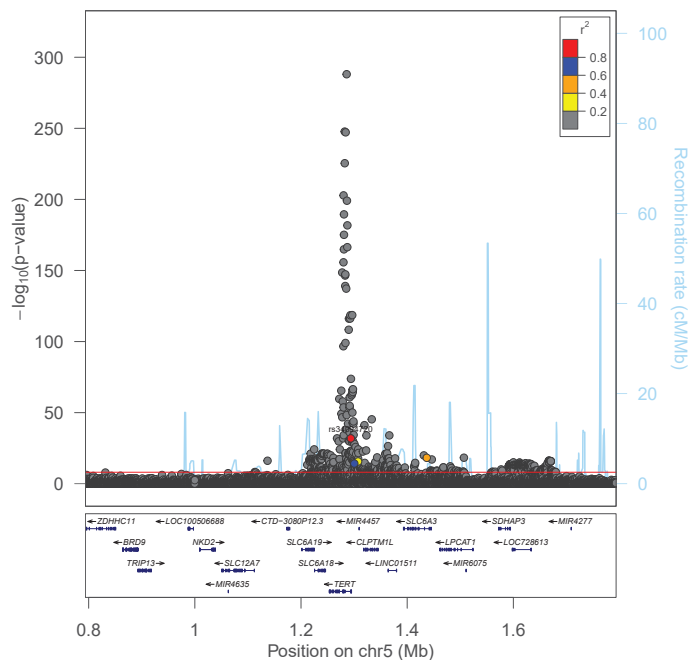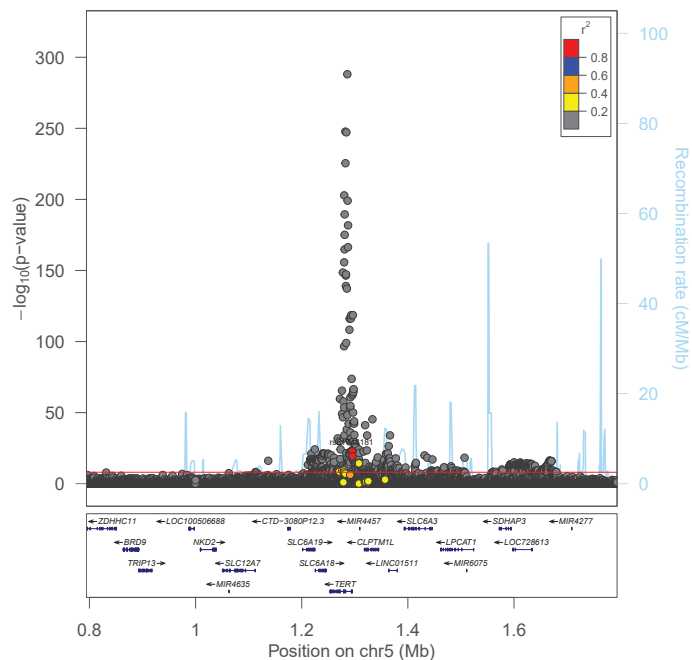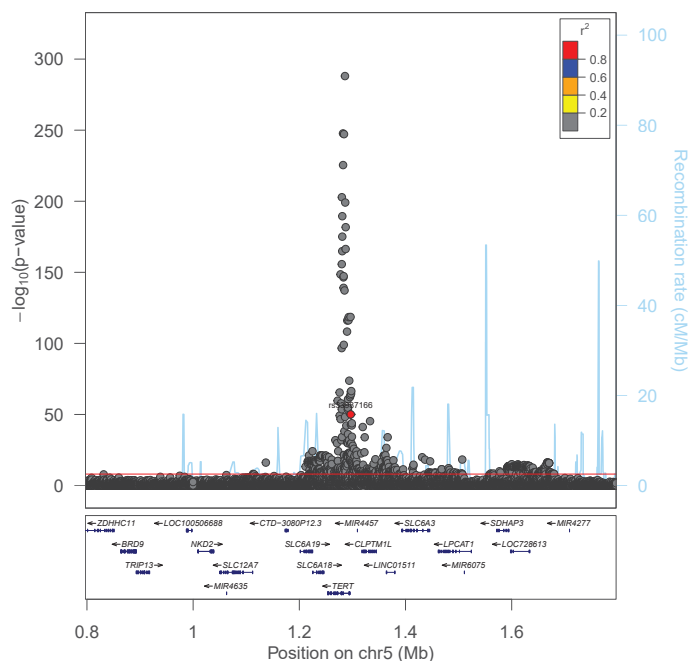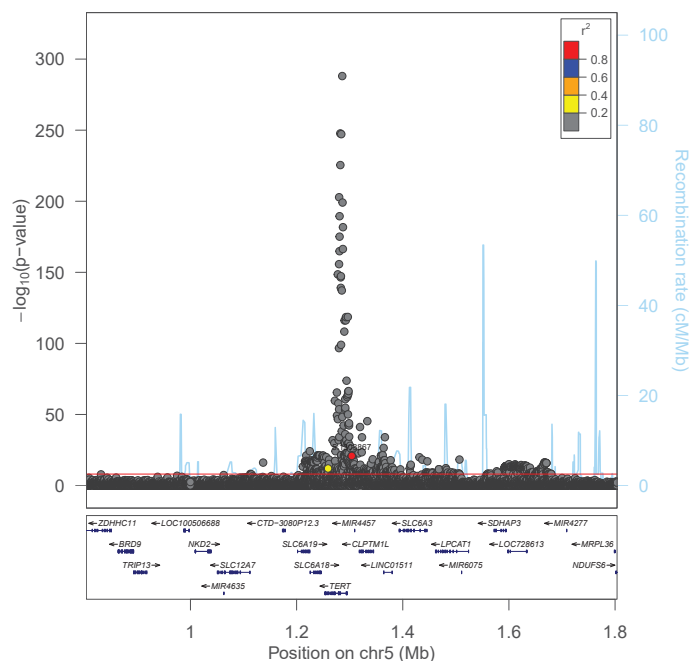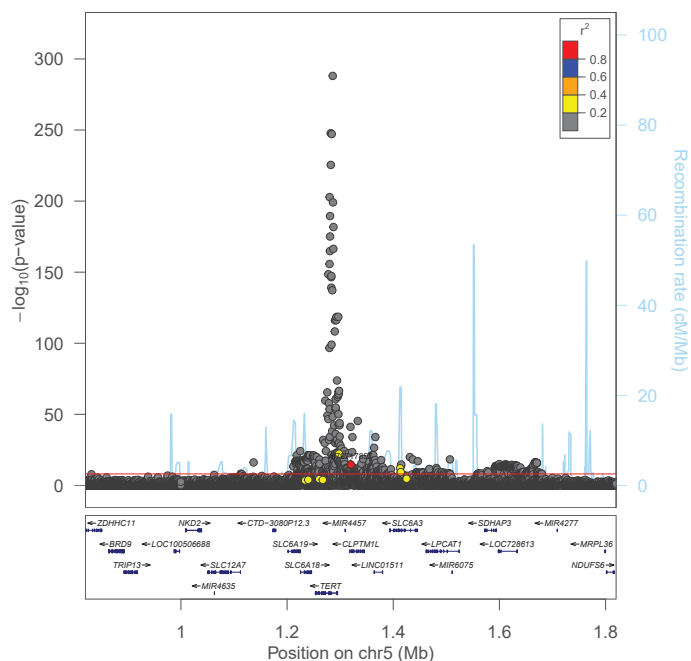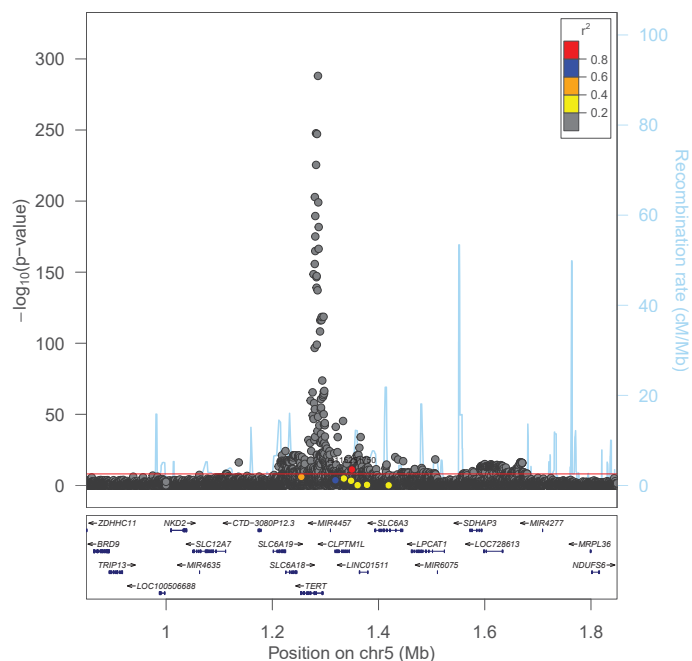

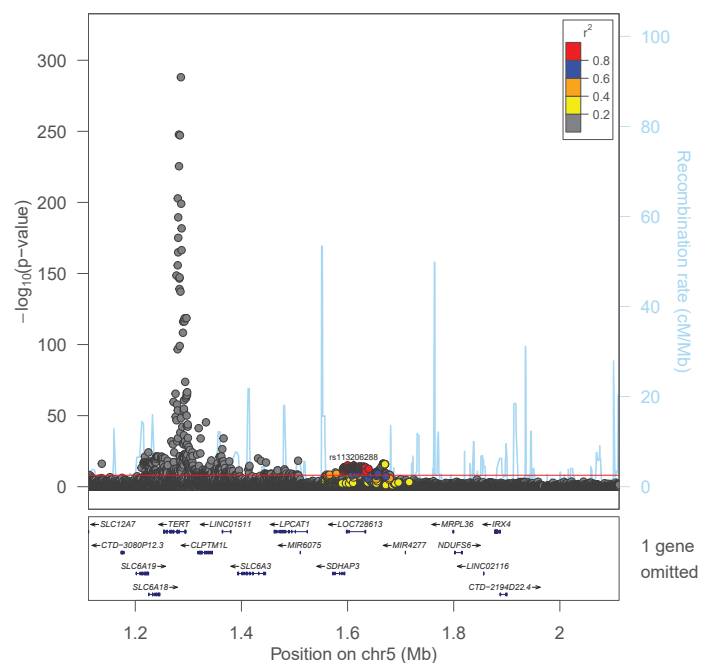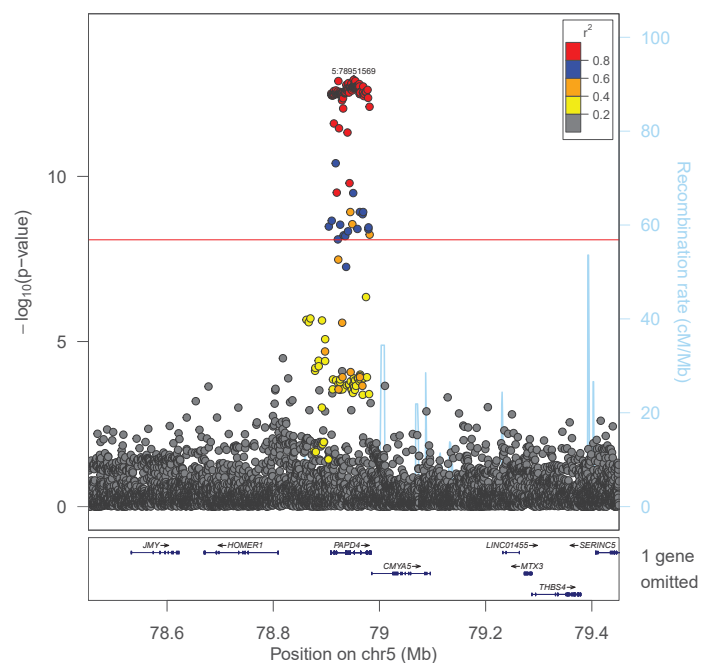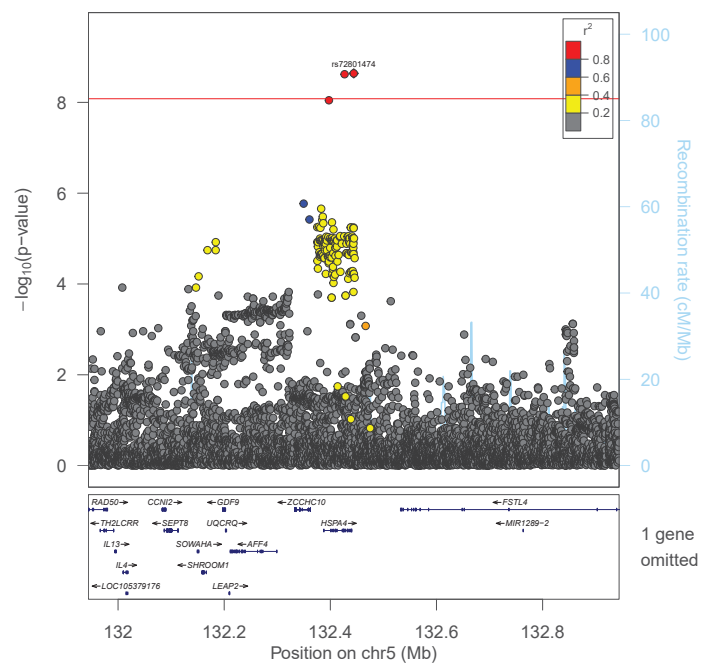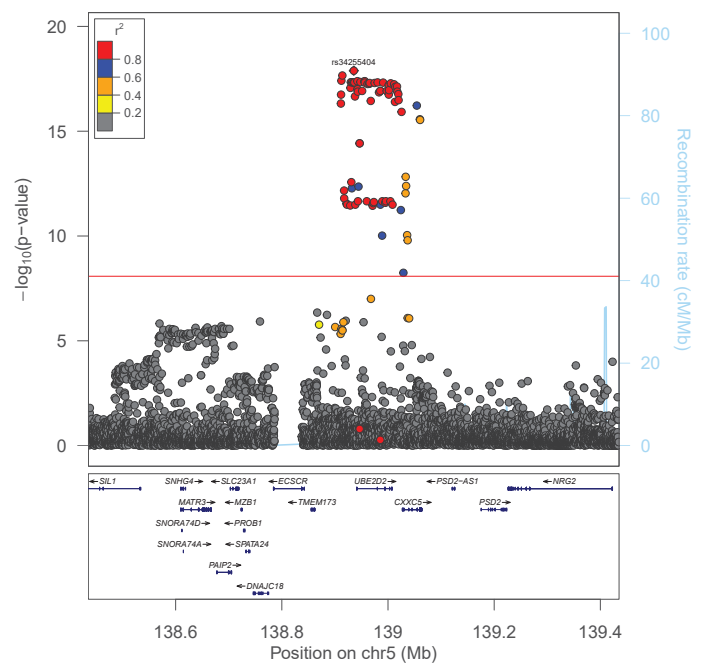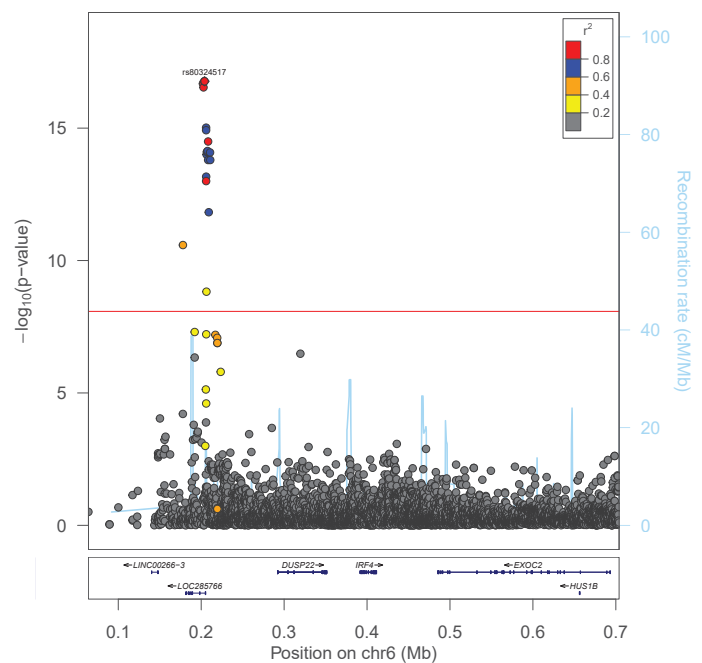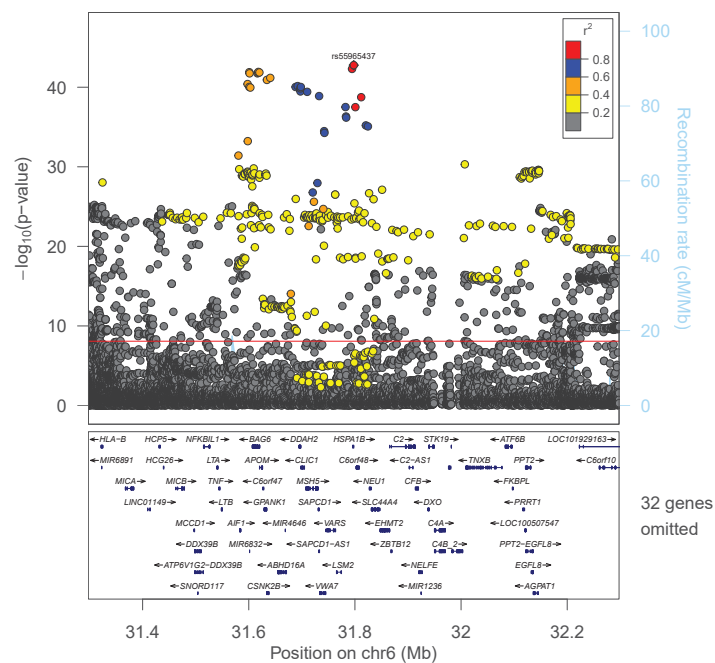

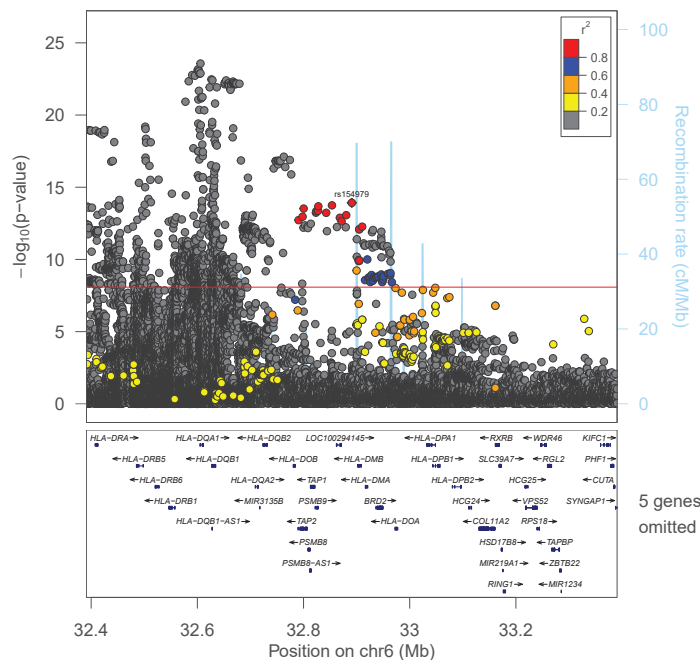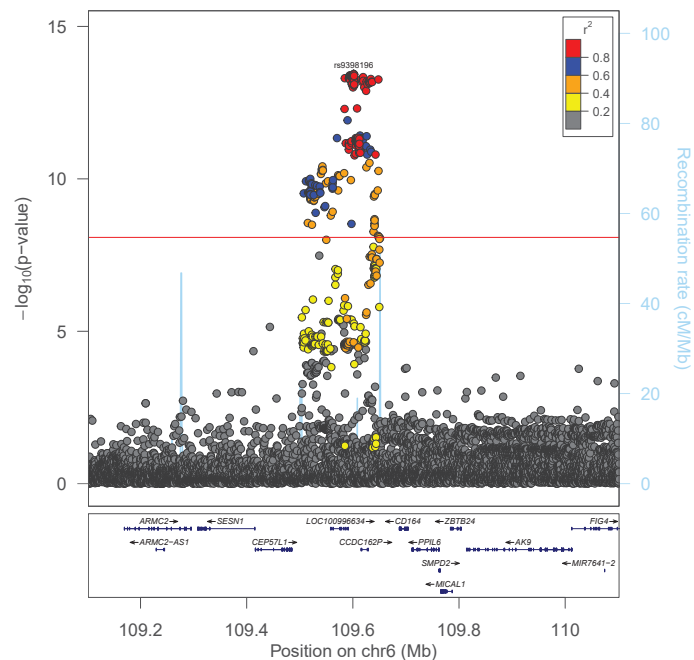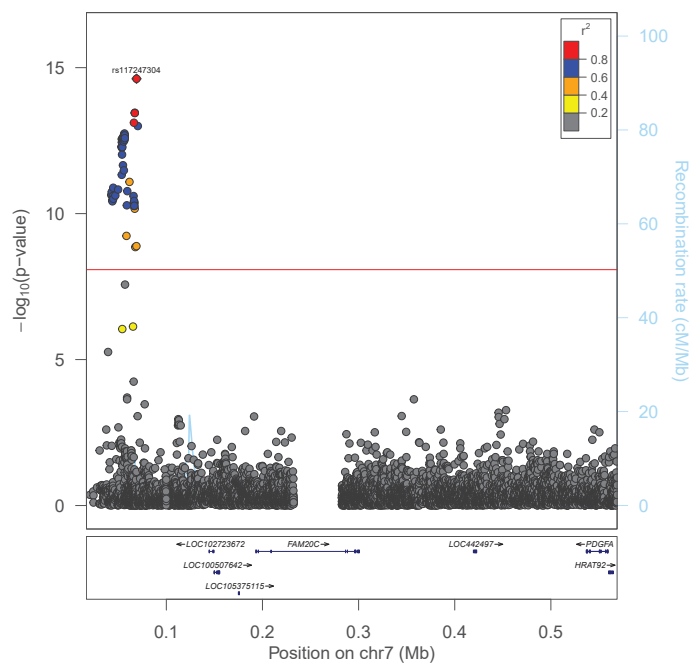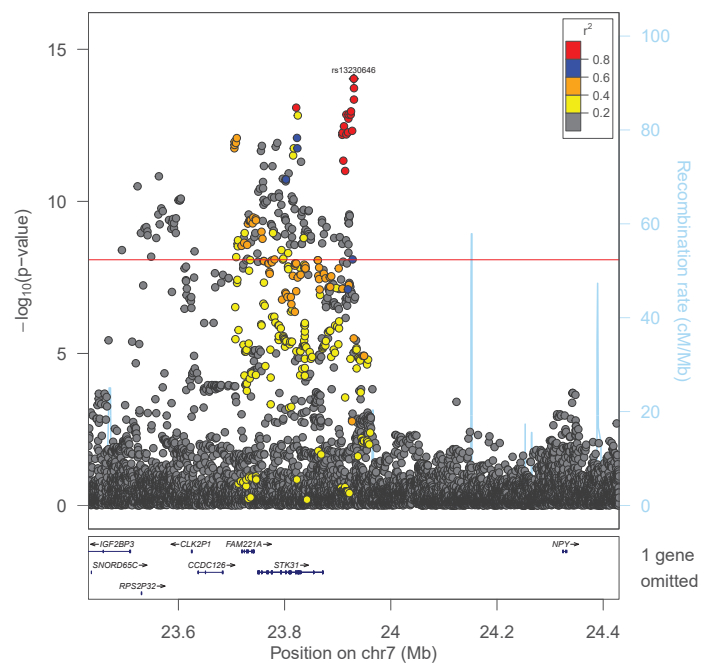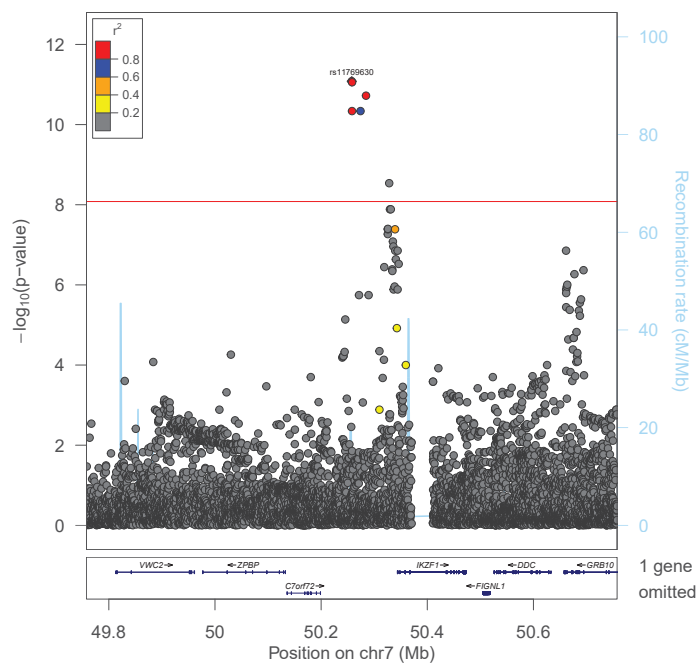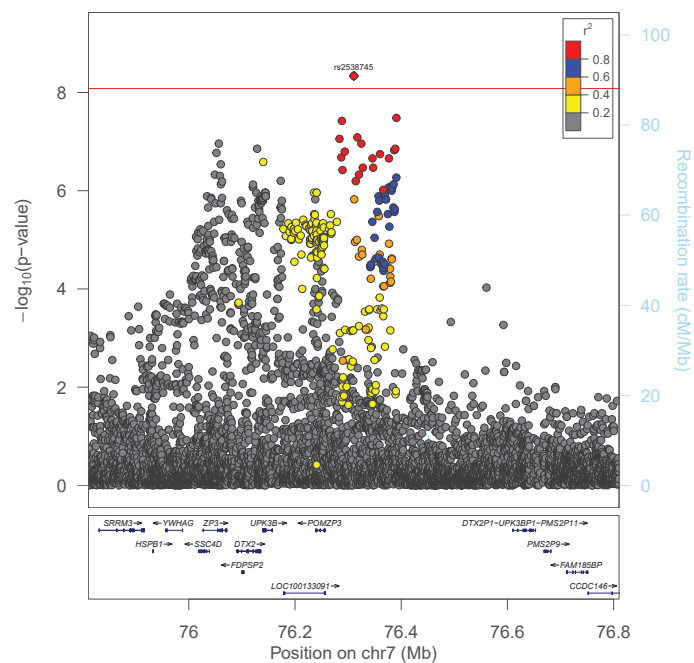

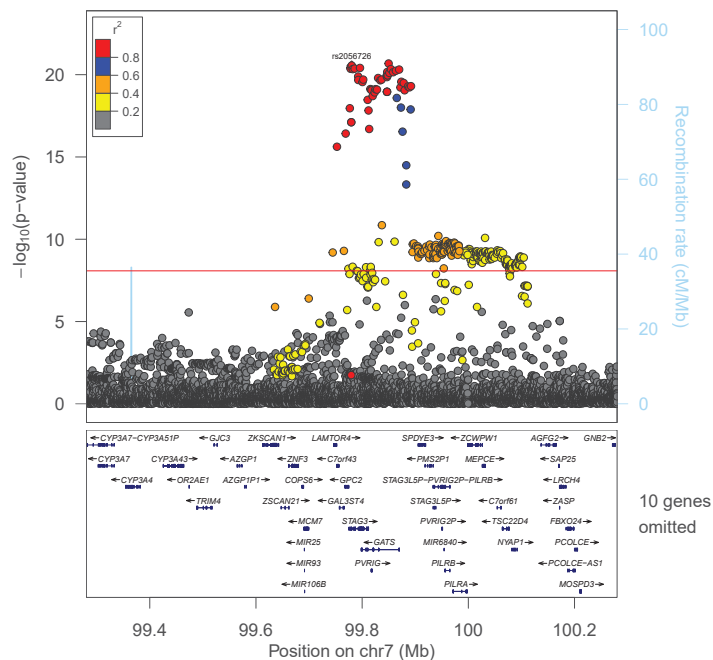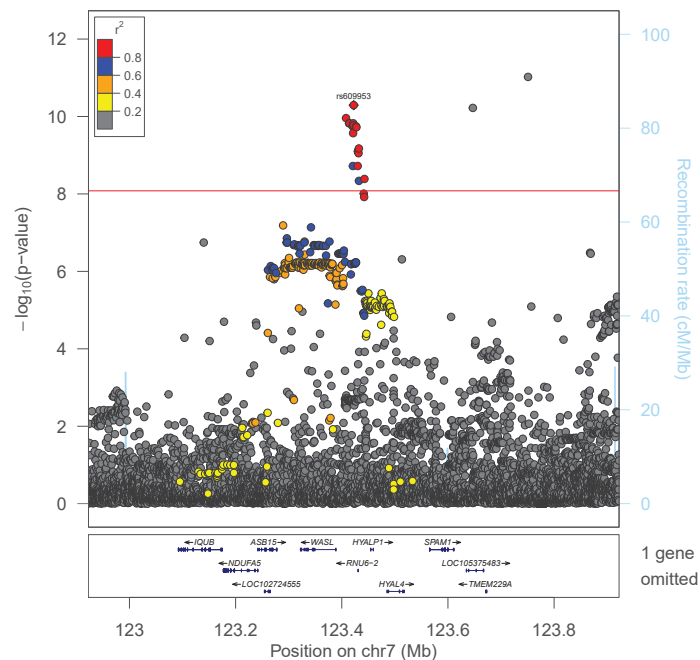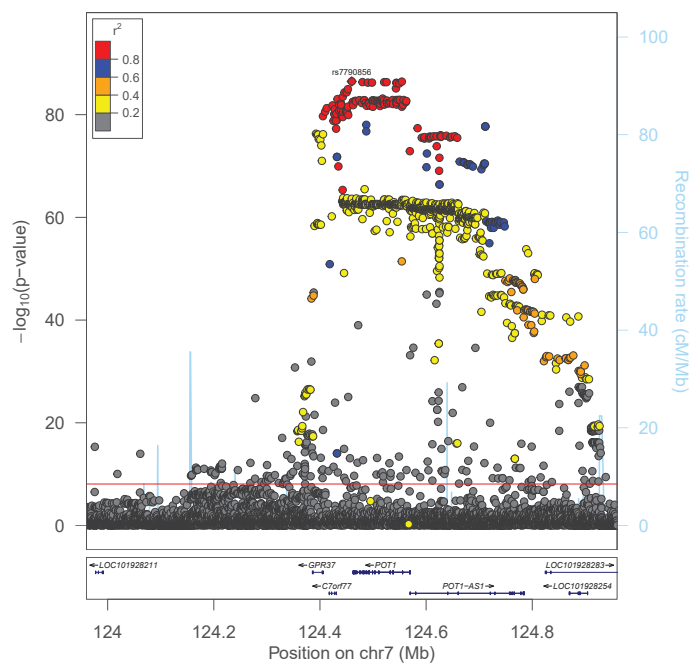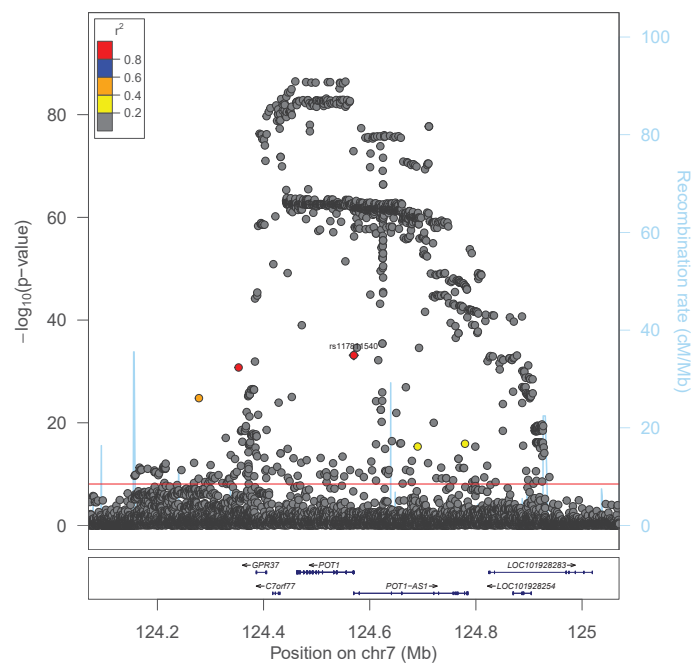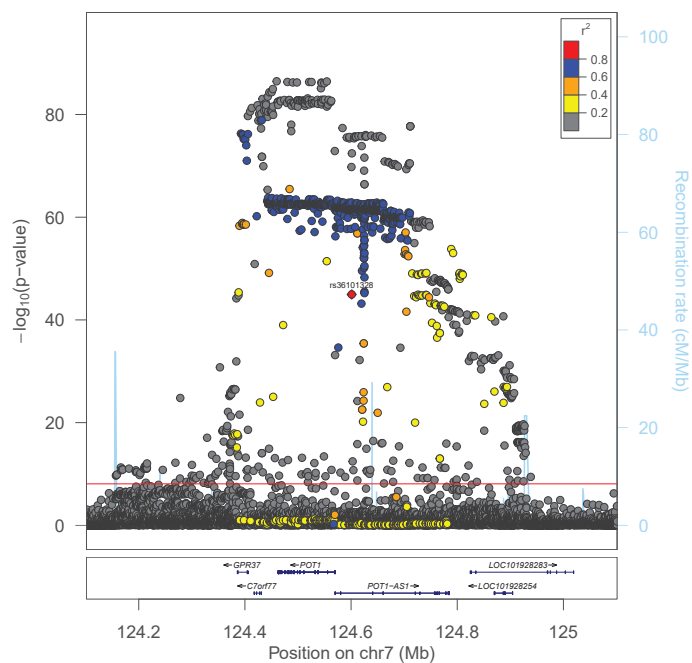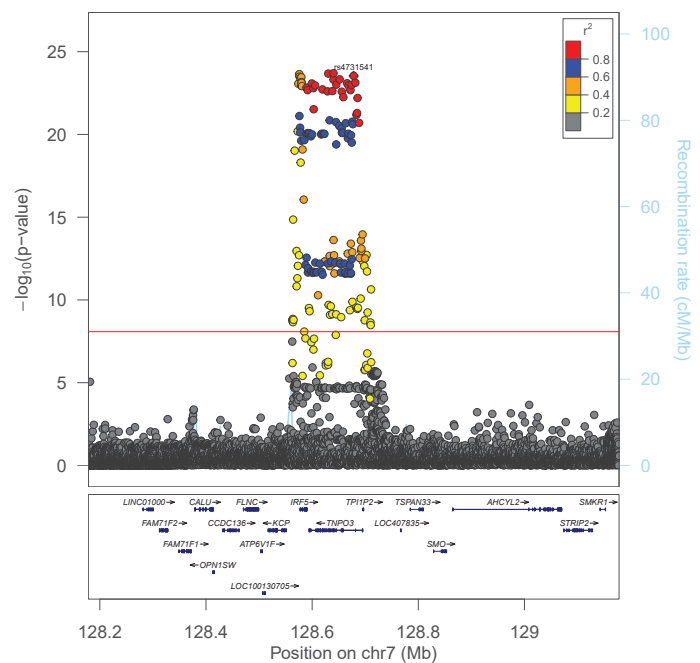

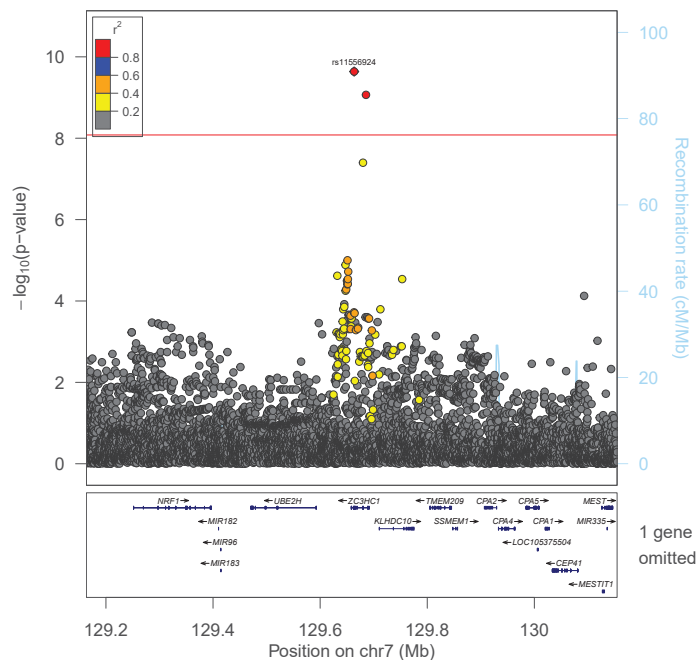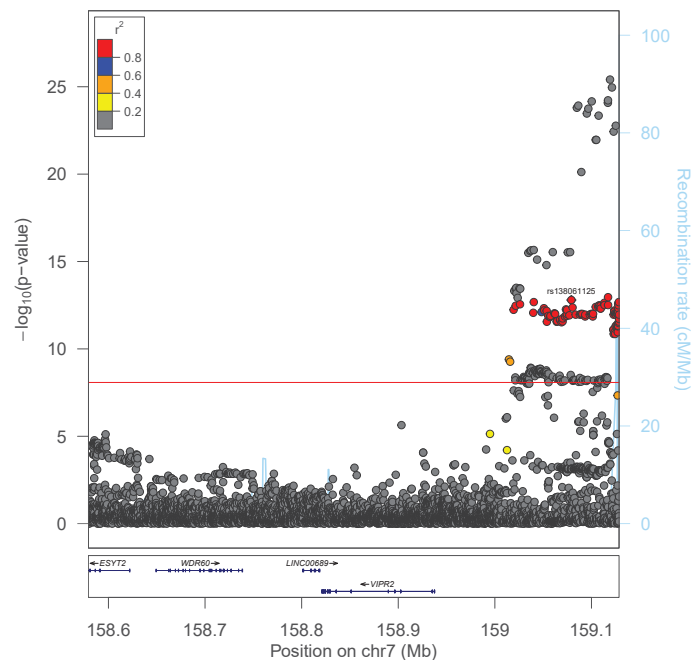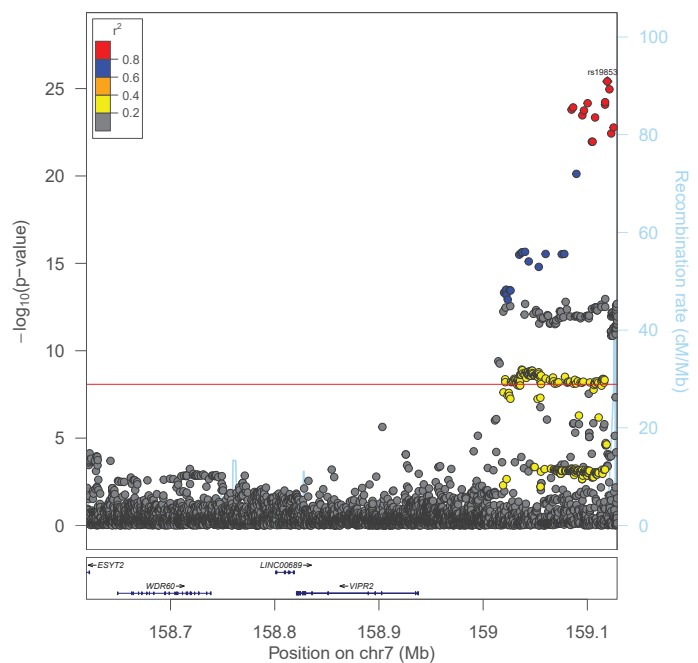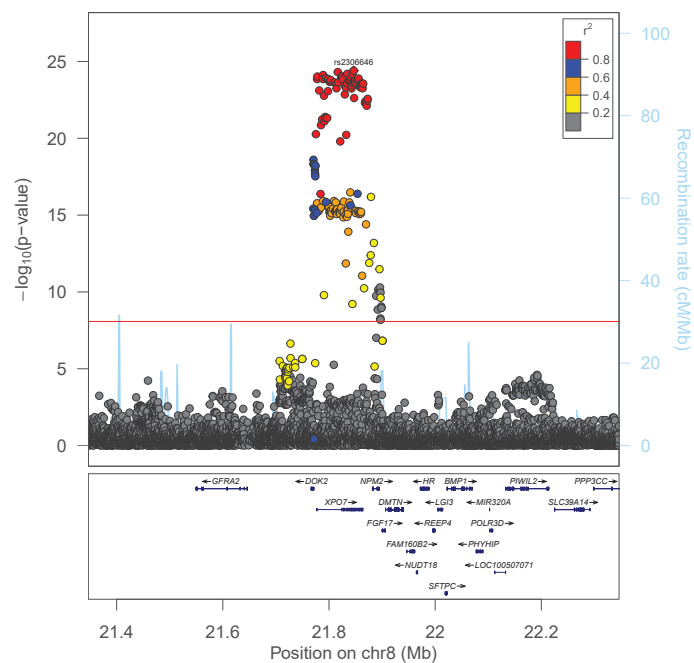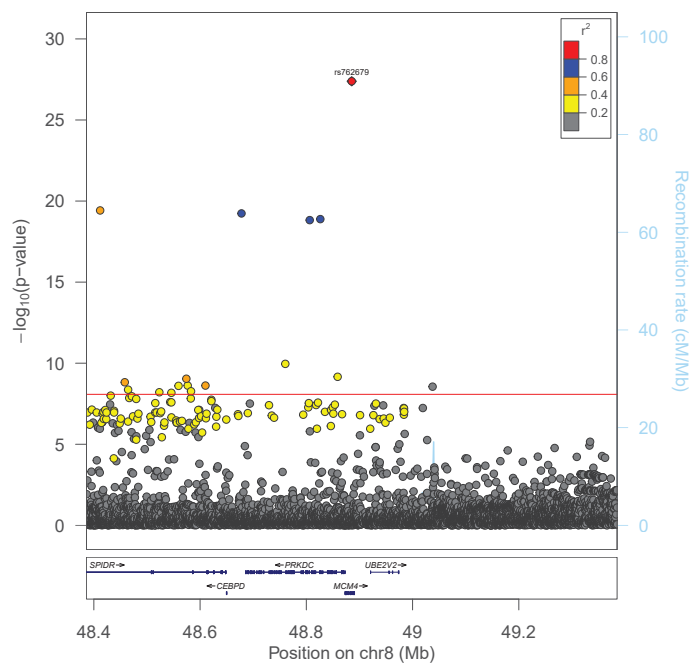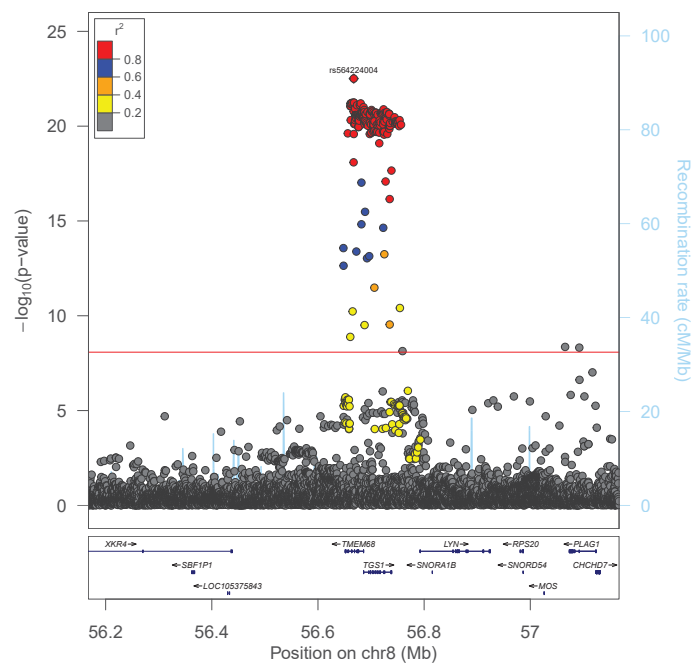

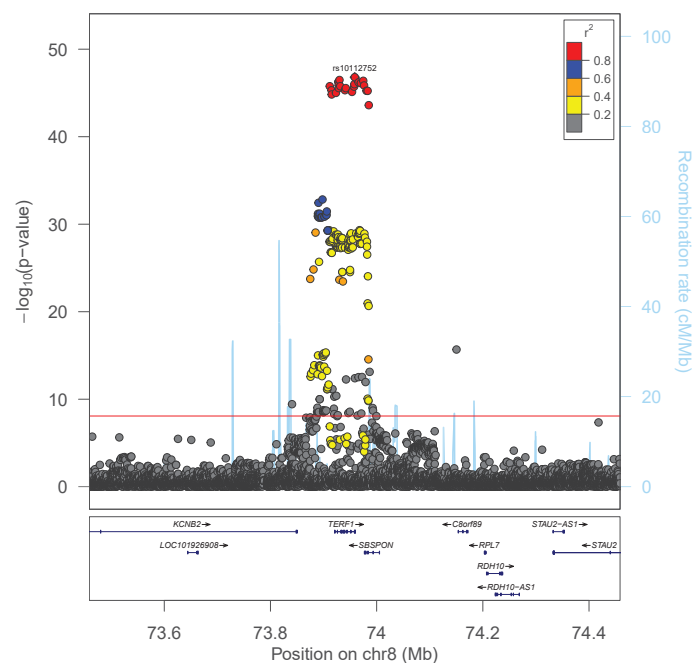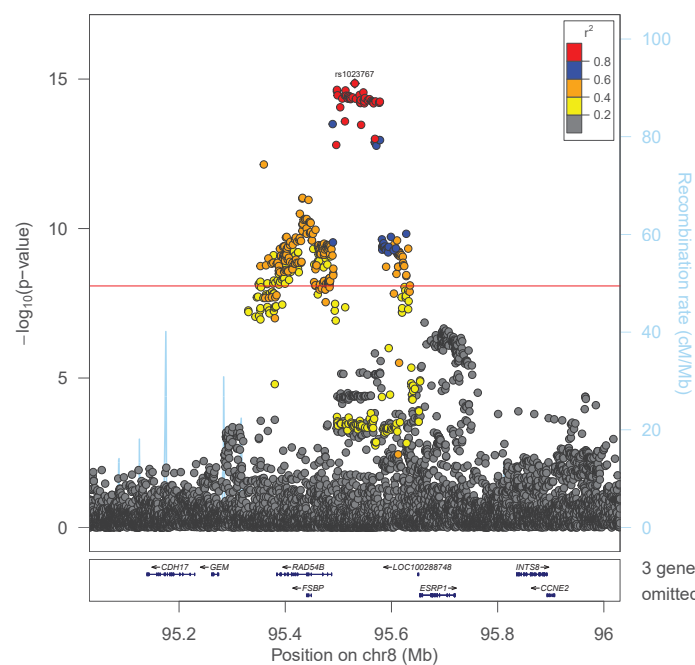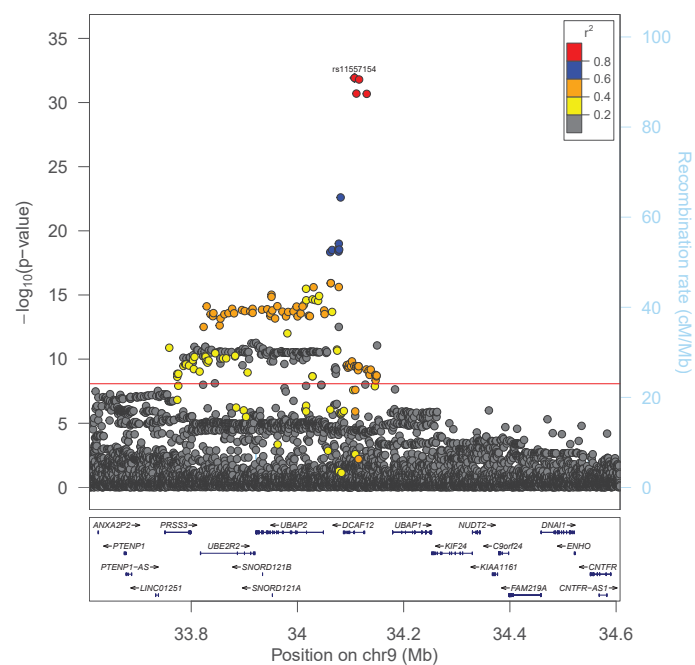

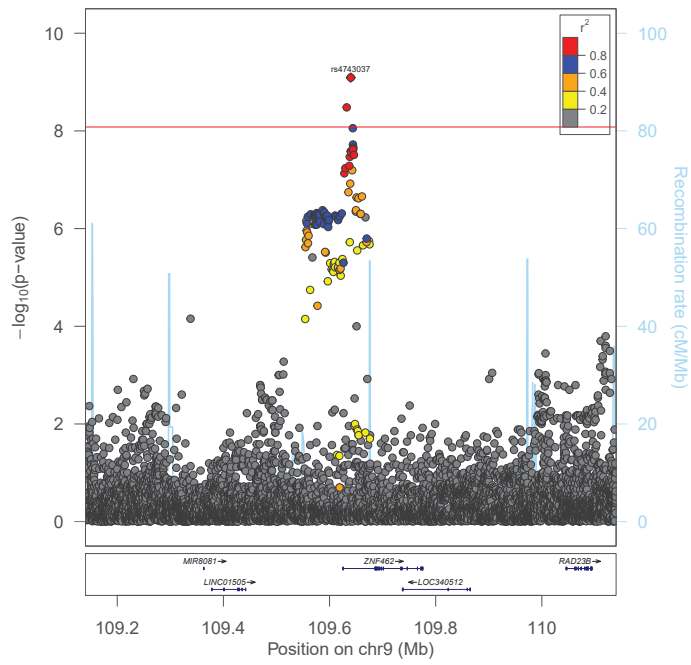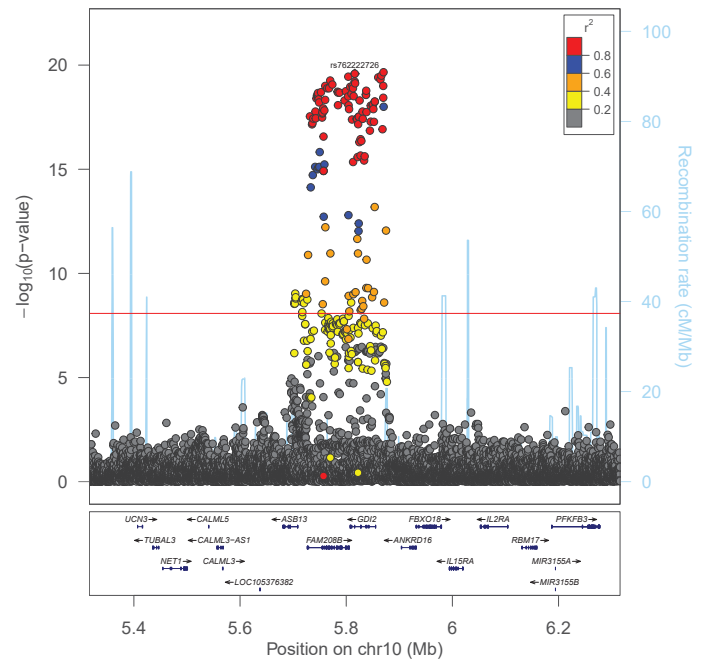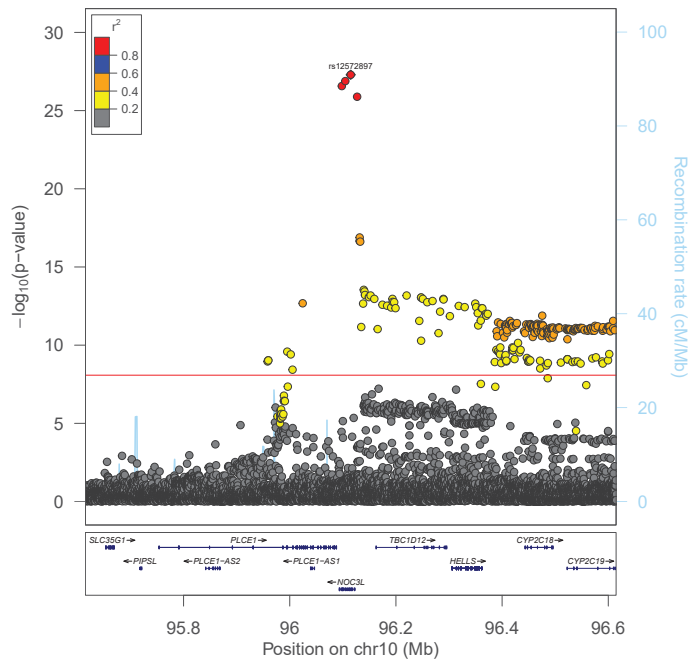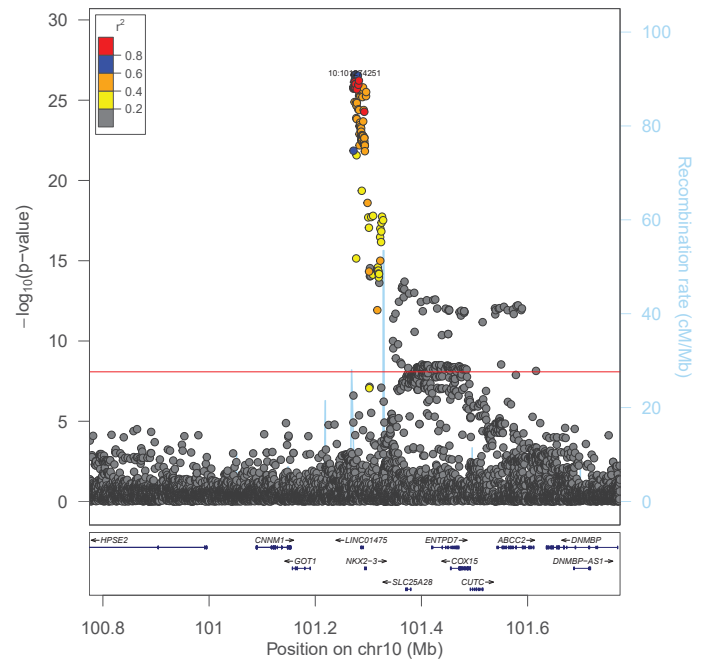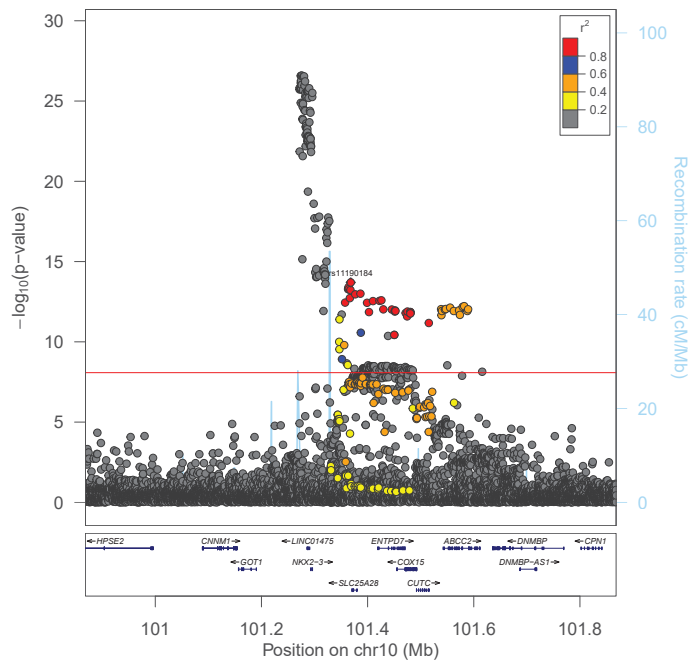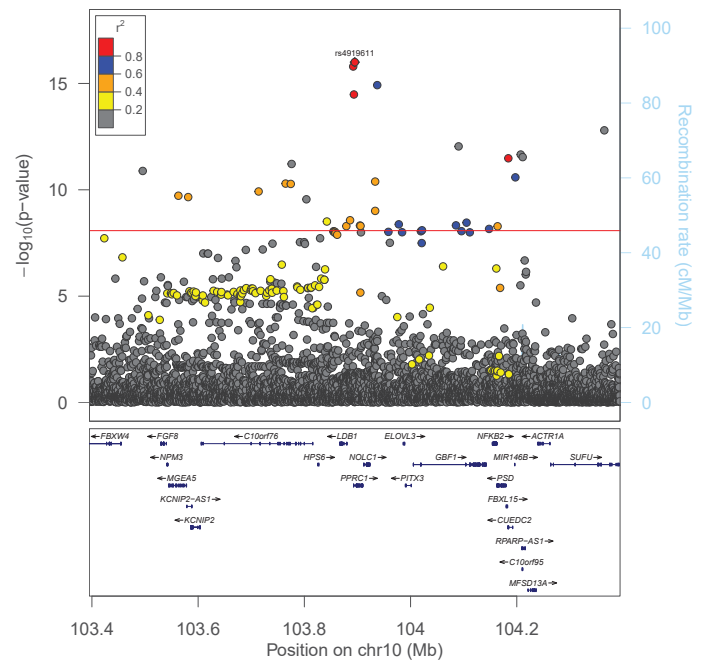

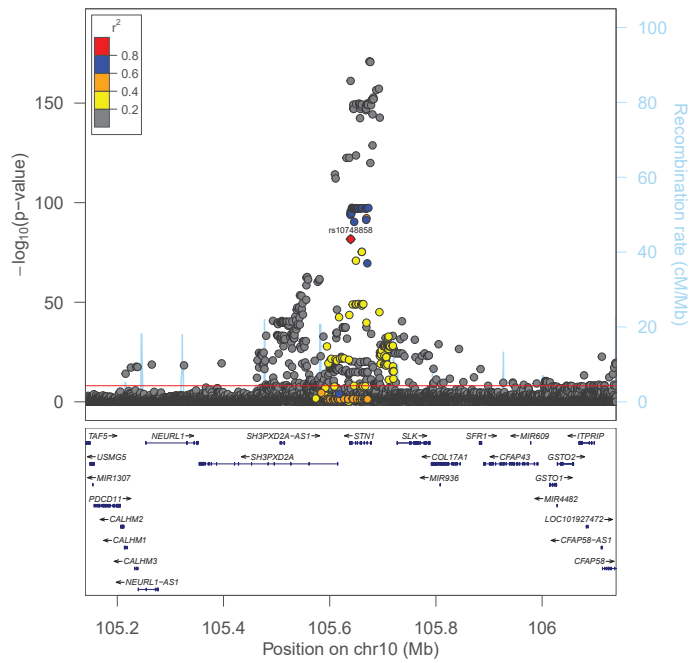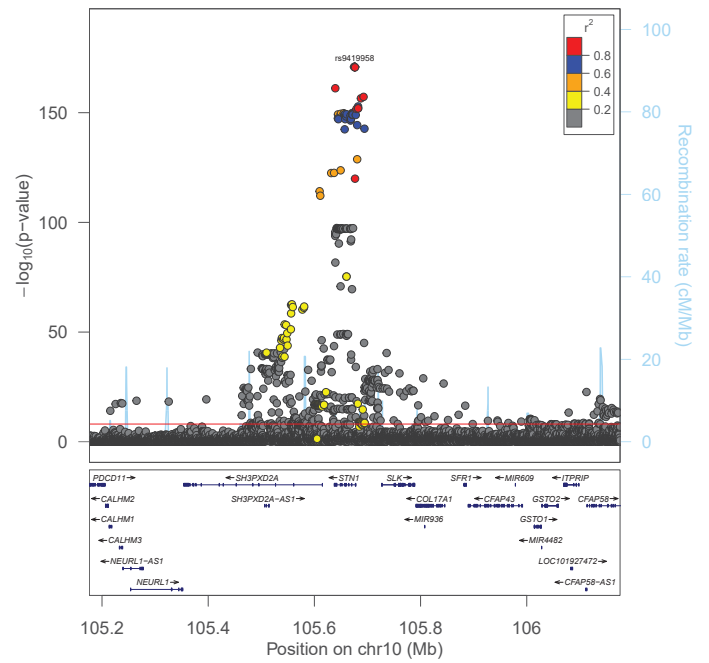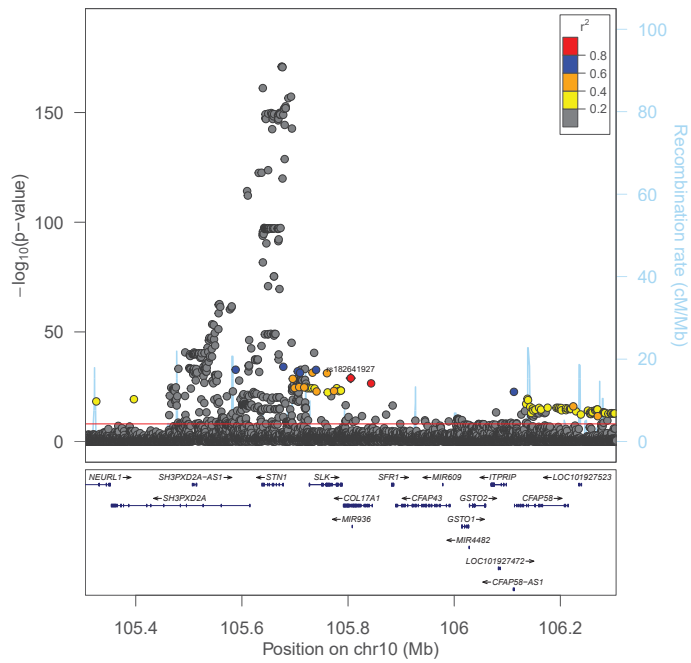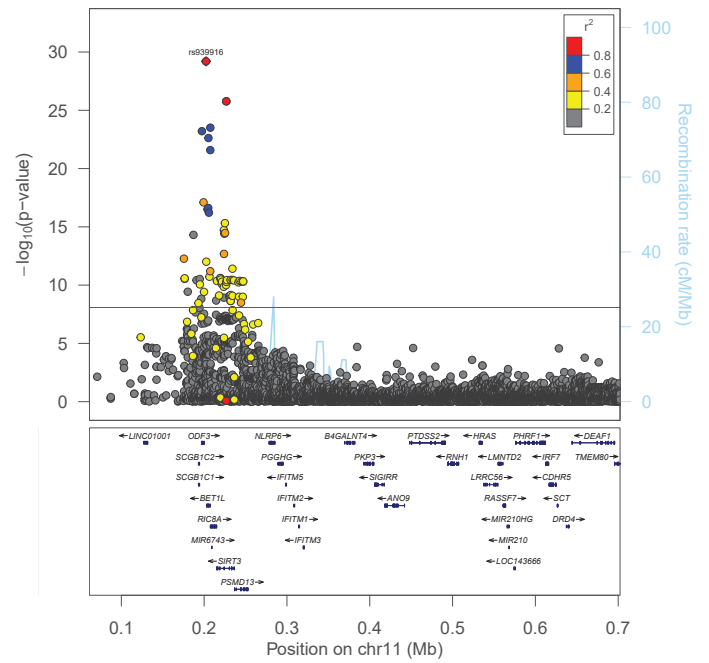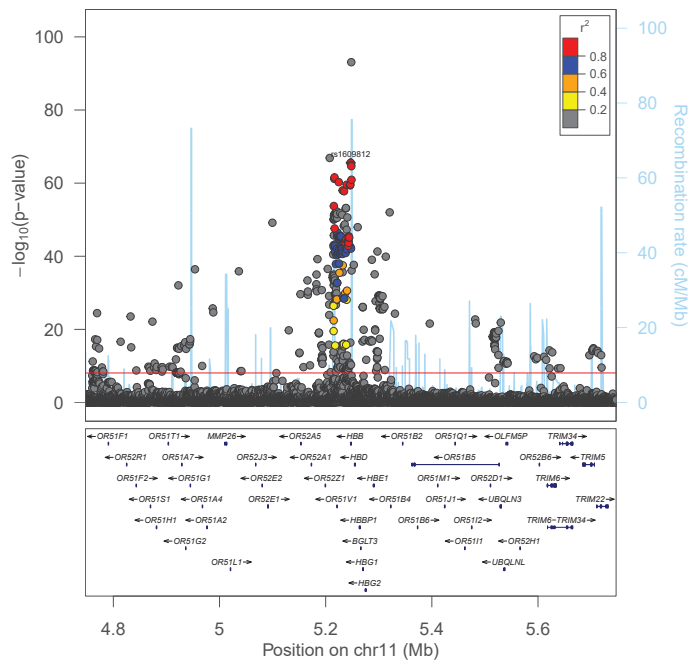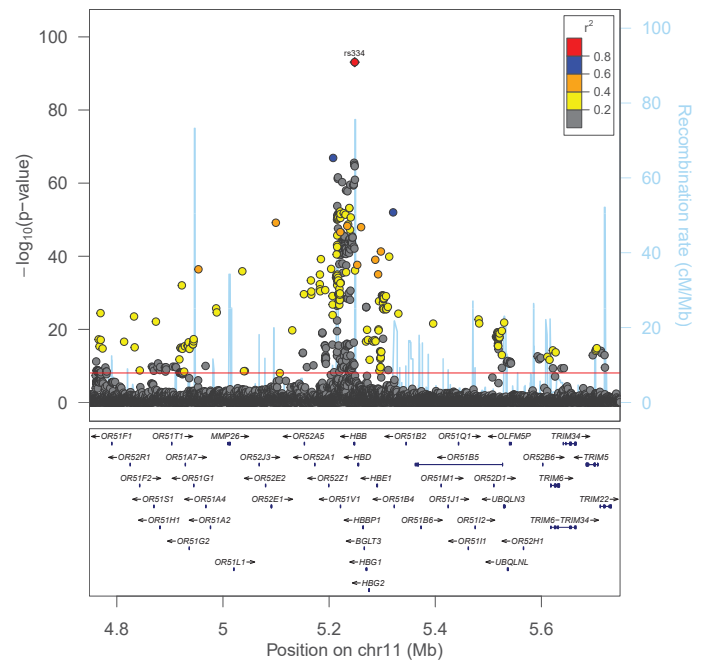

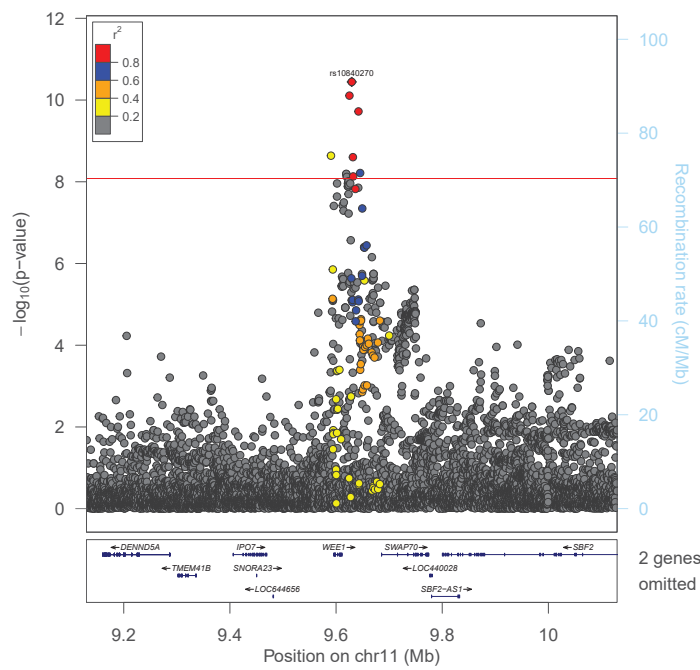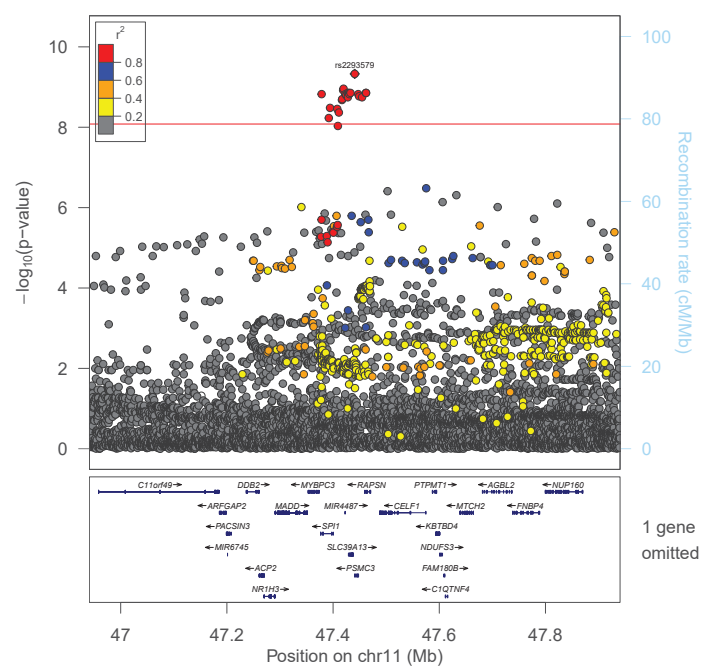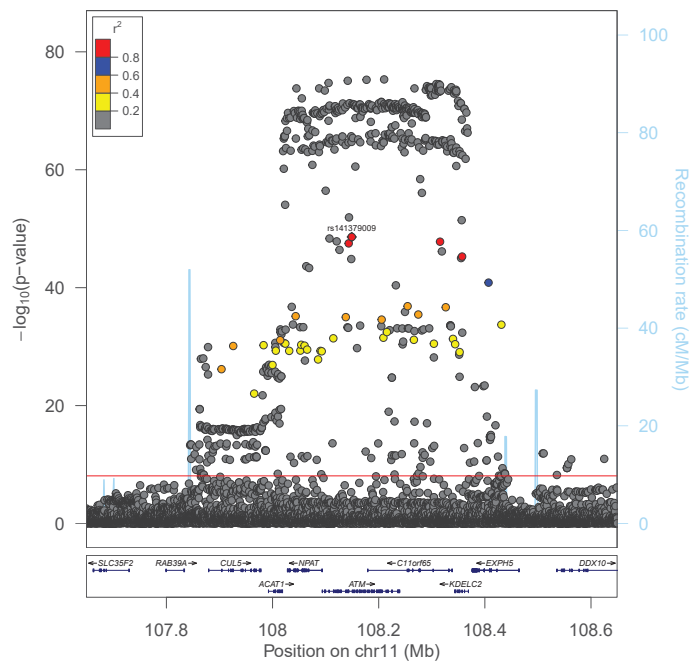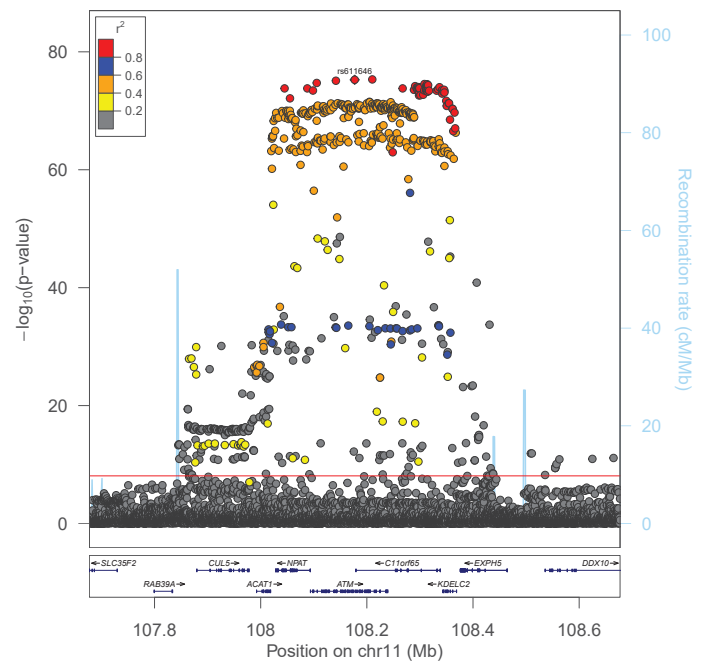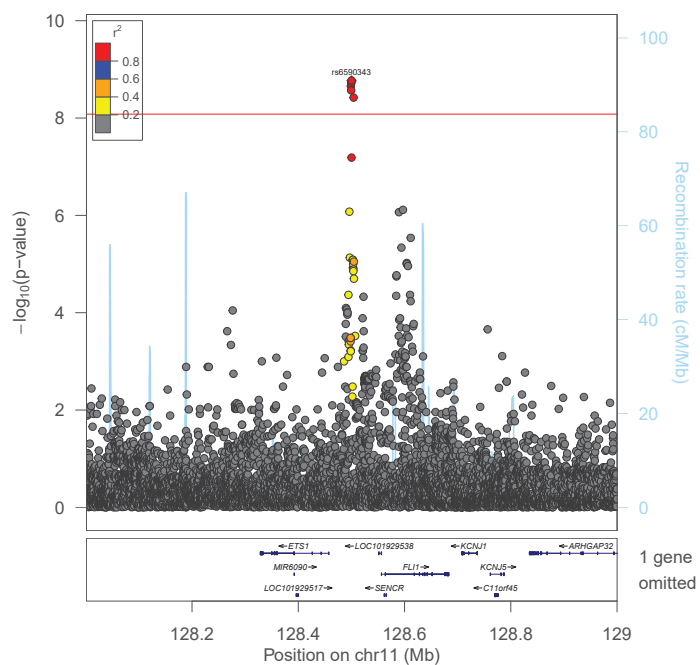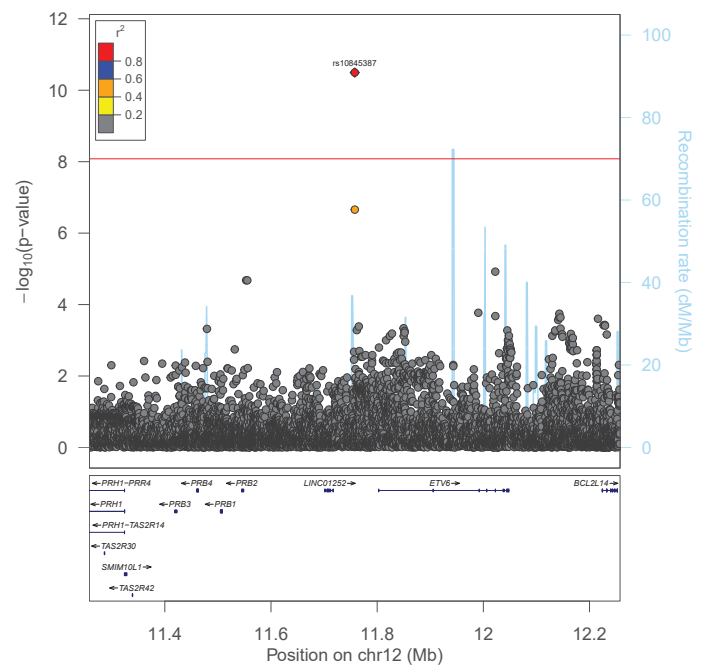

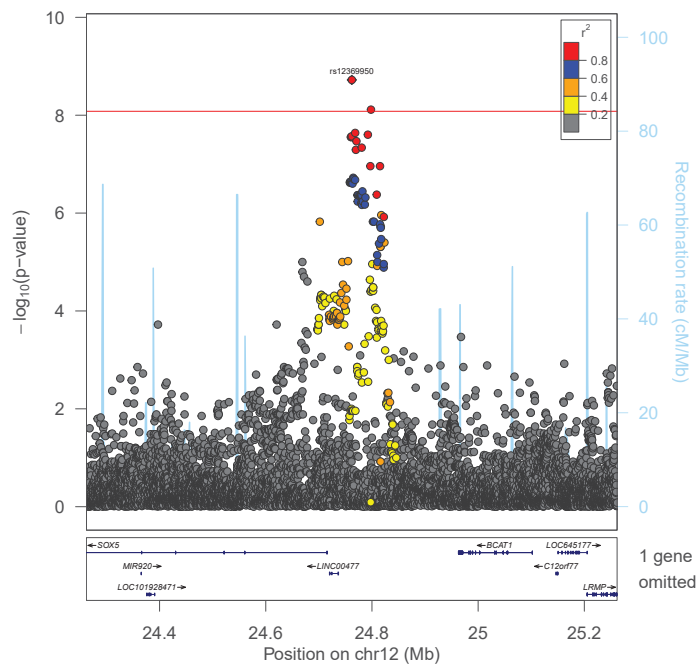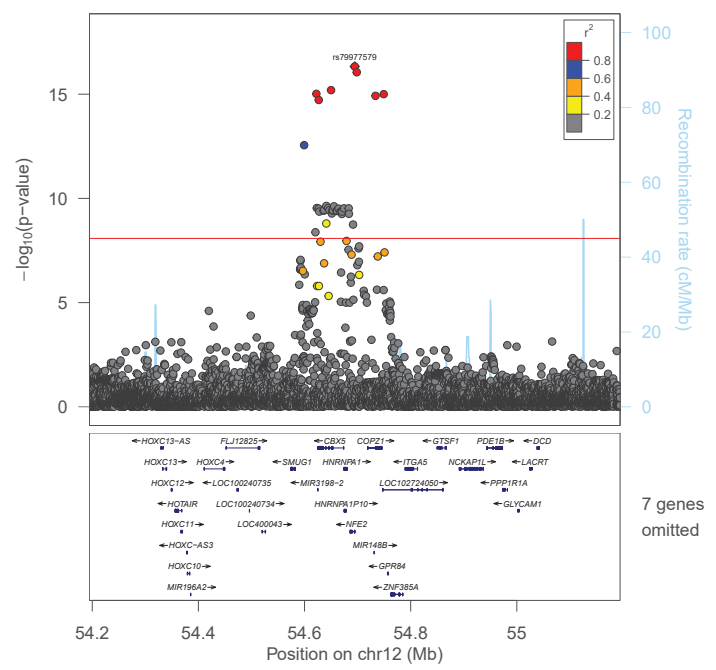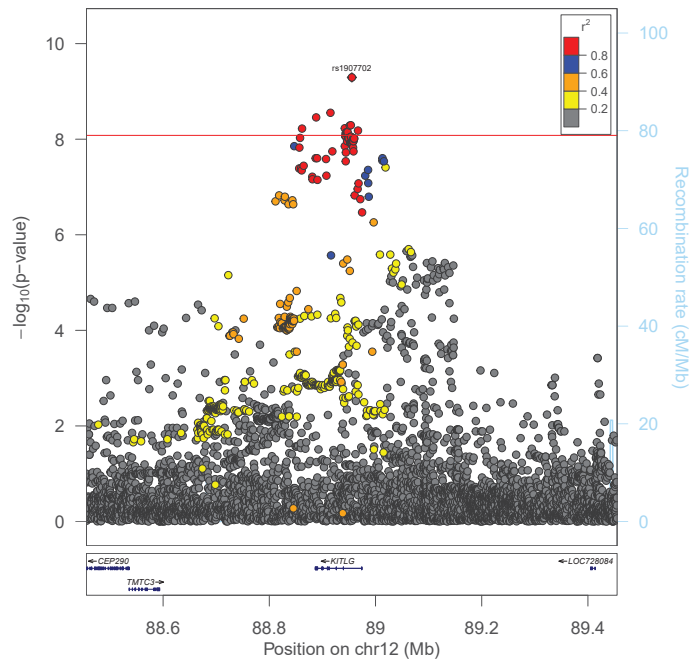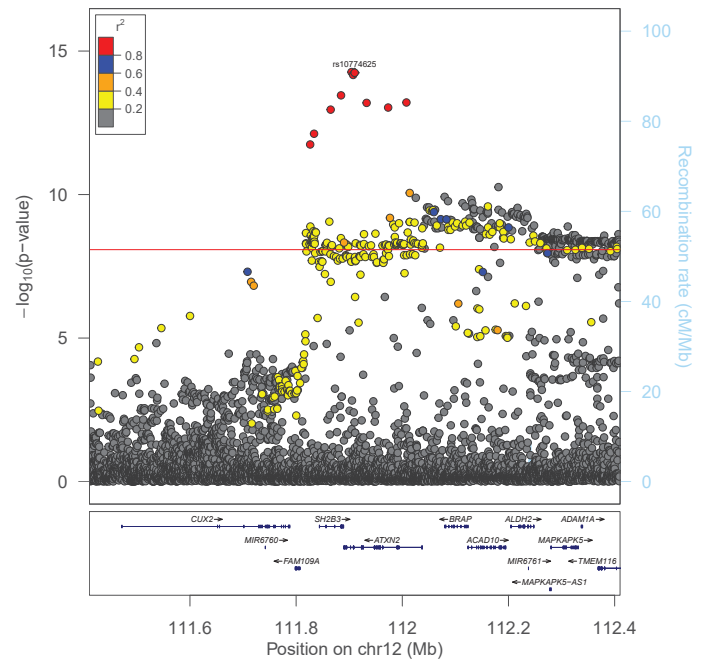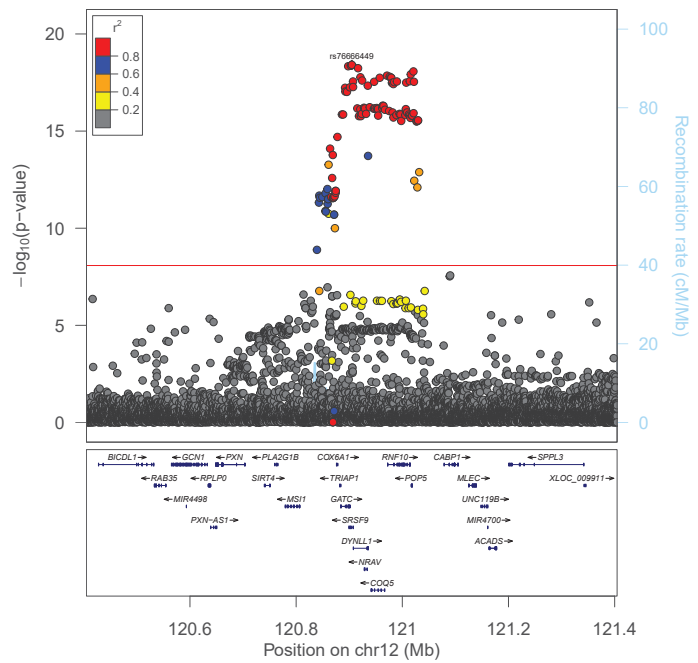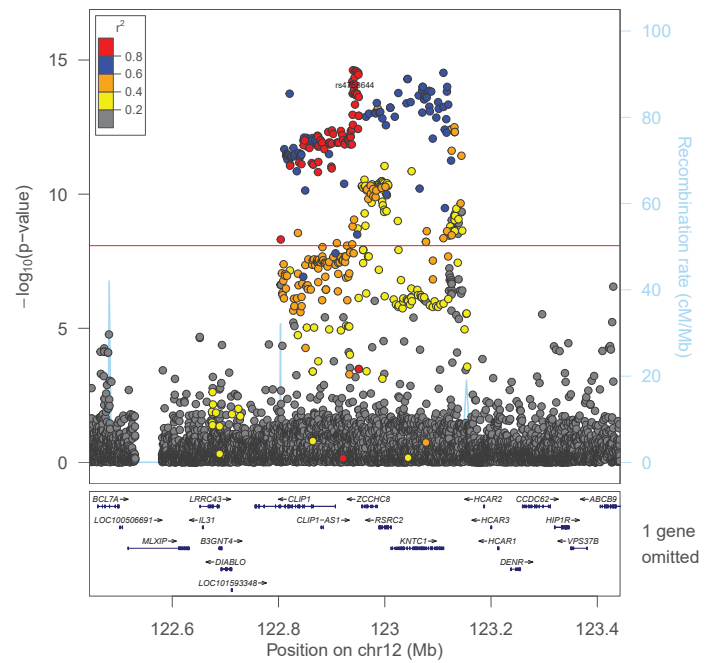

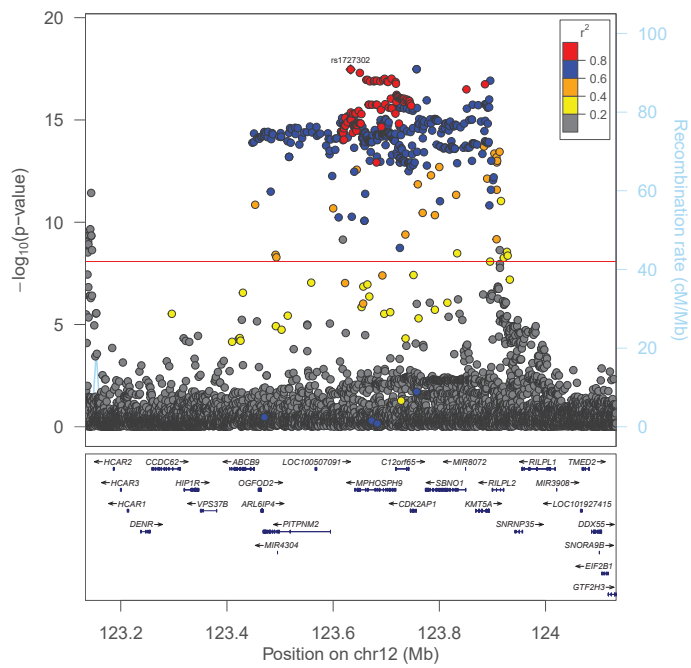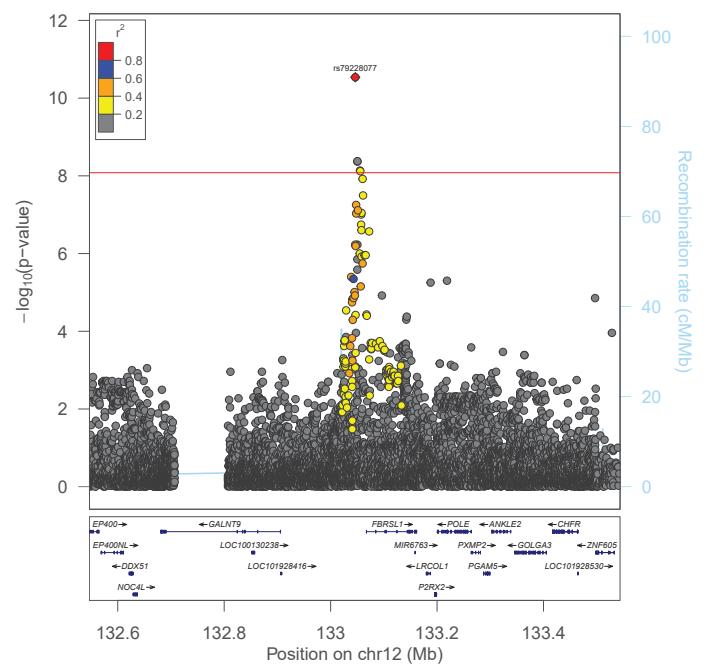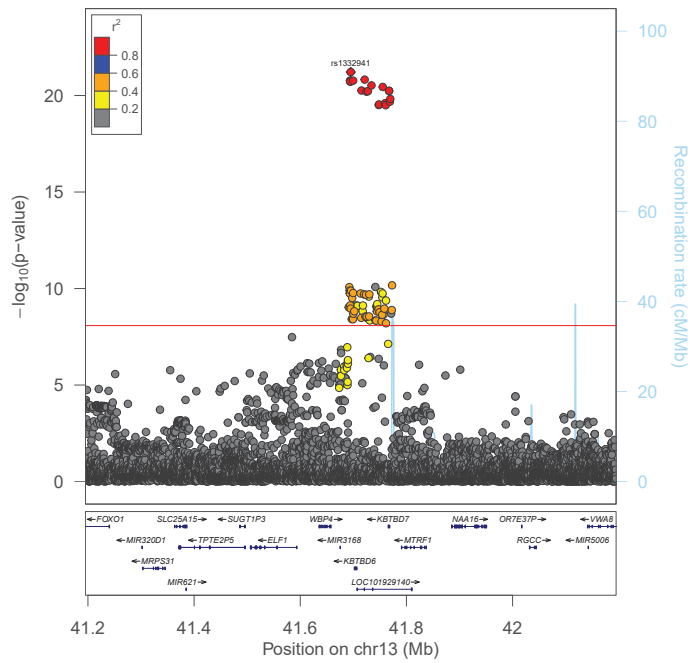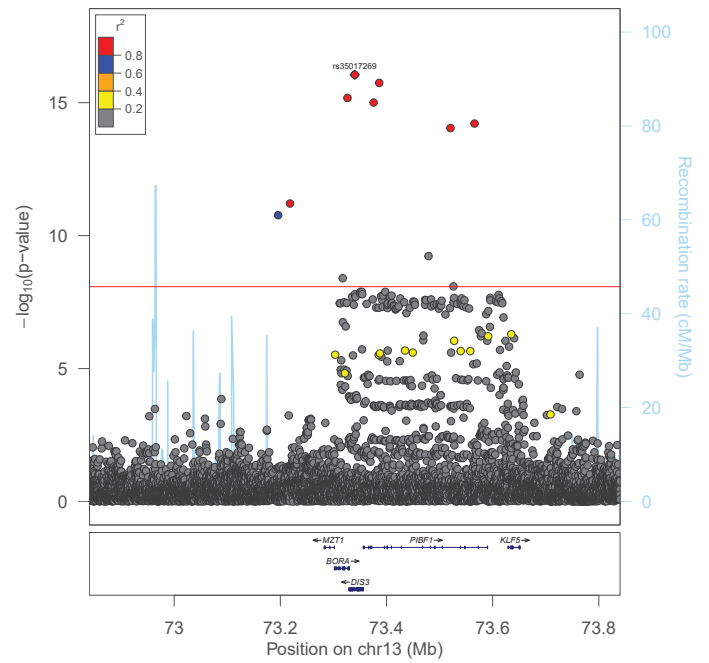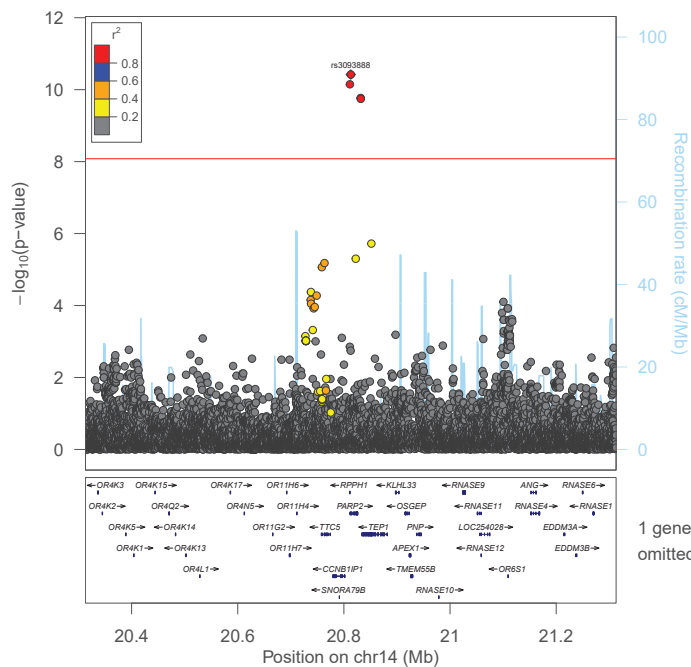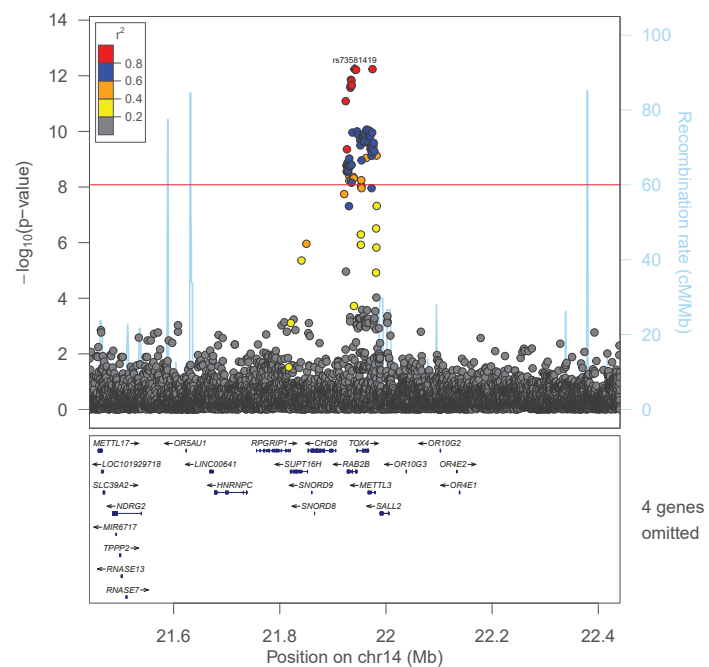

1 gene omitted

4 genes omitted

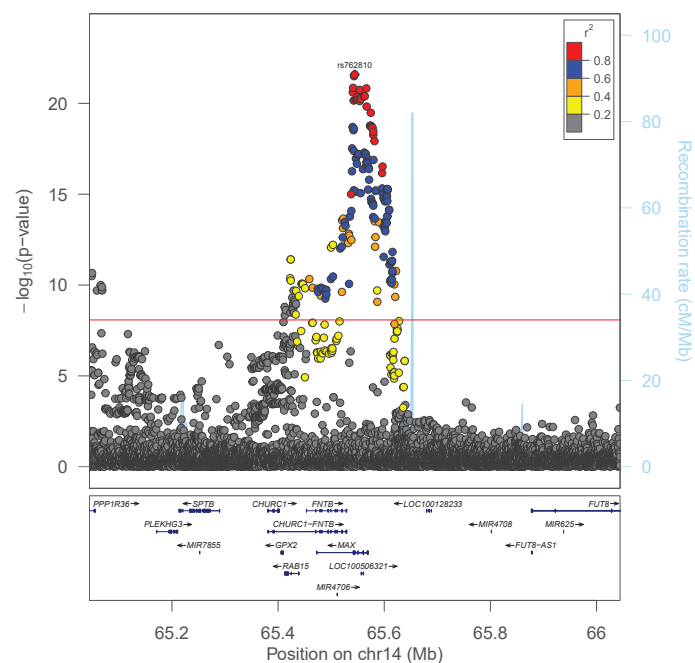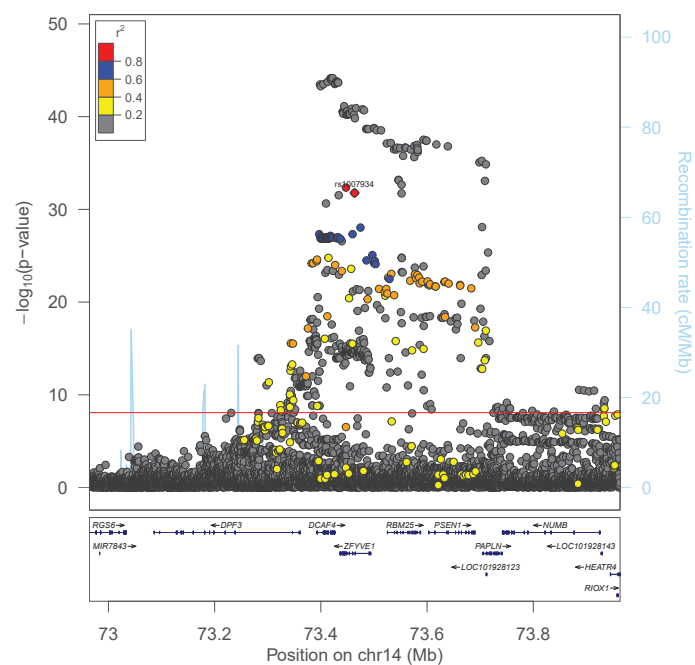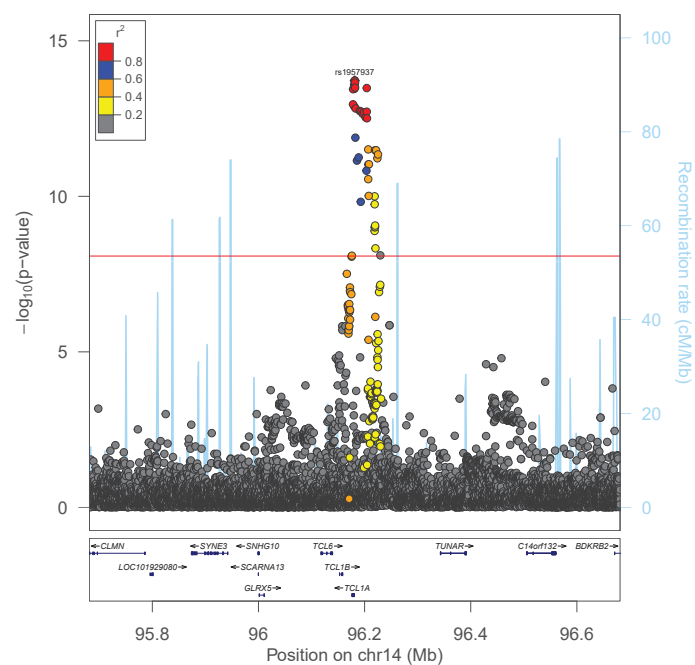

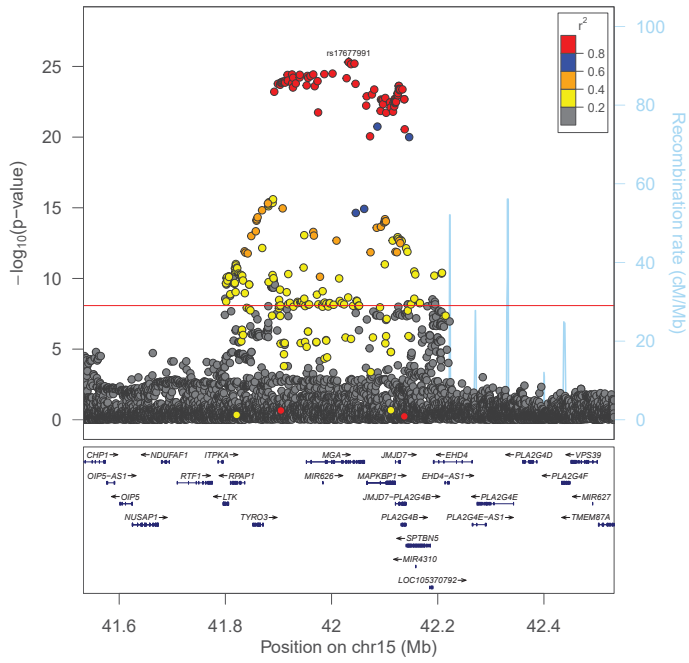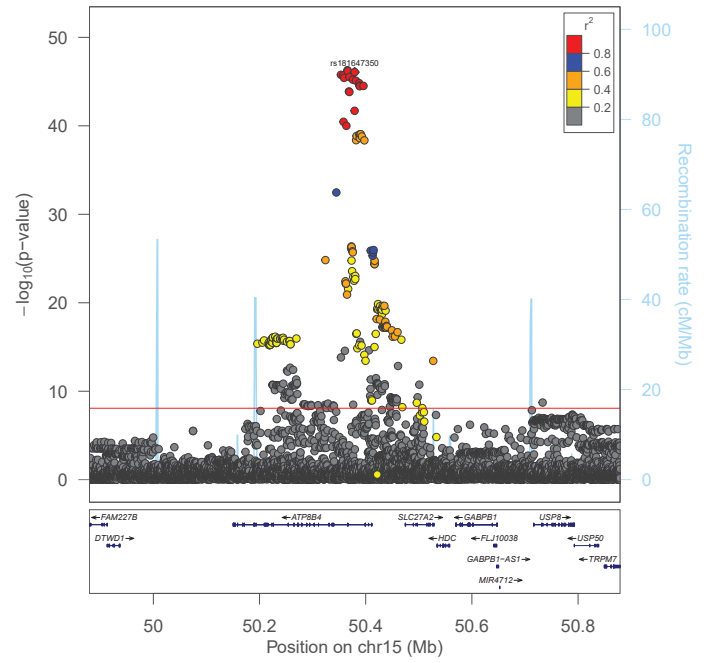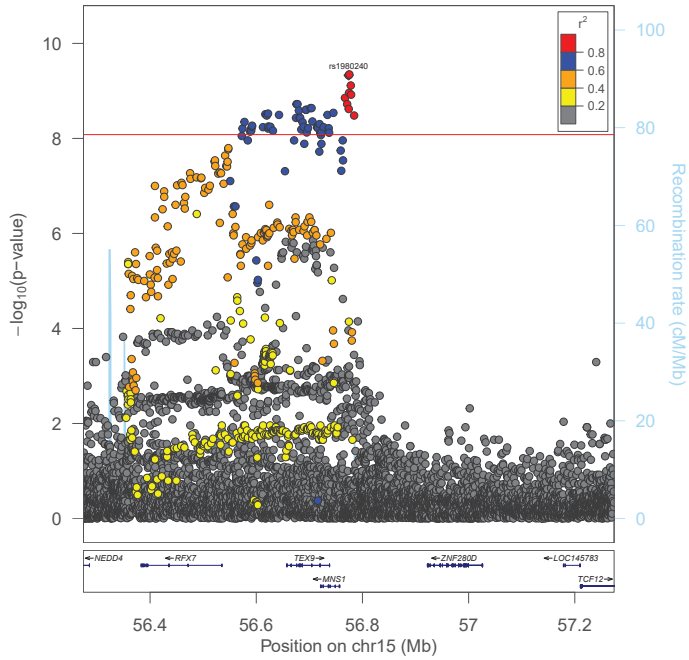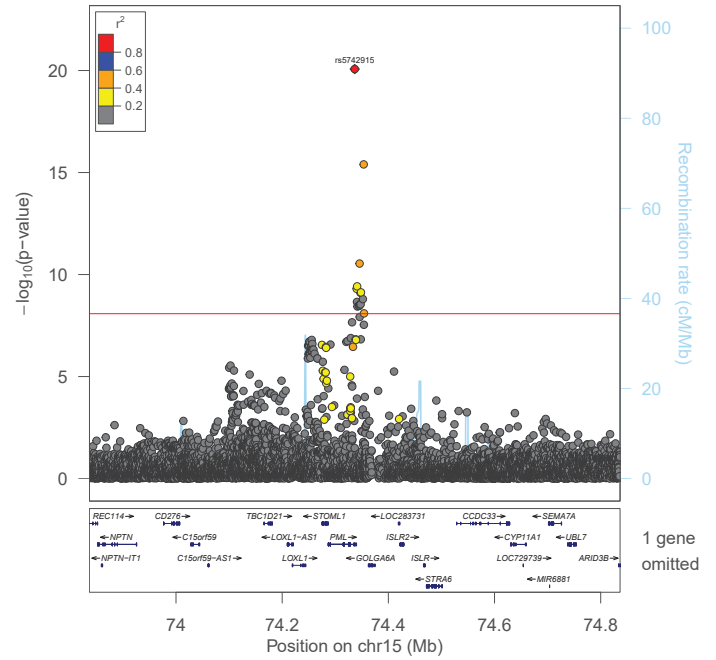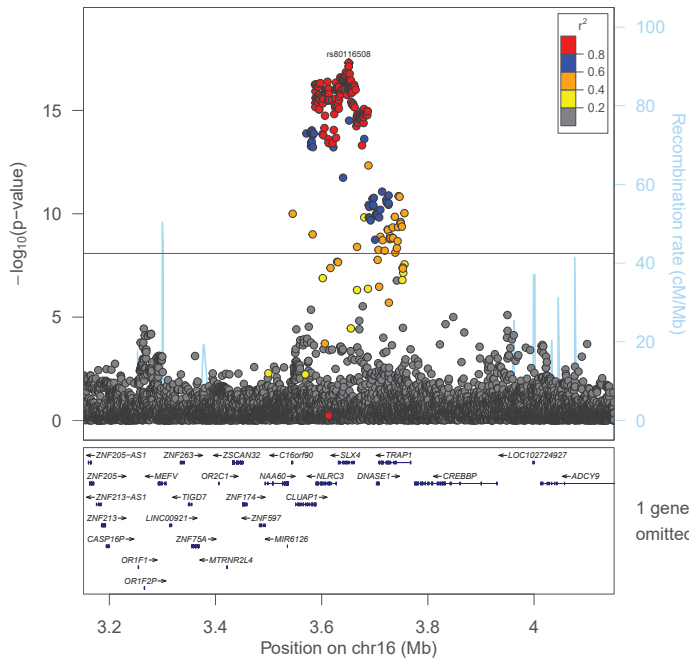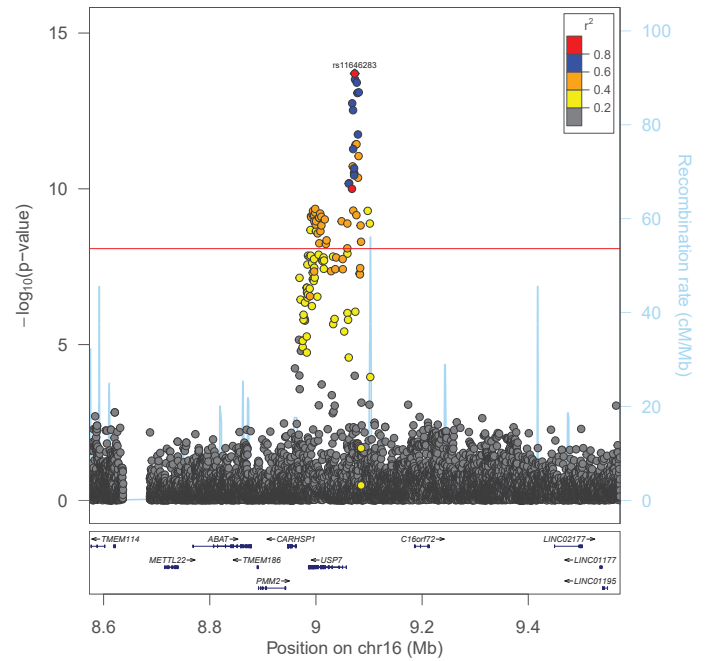

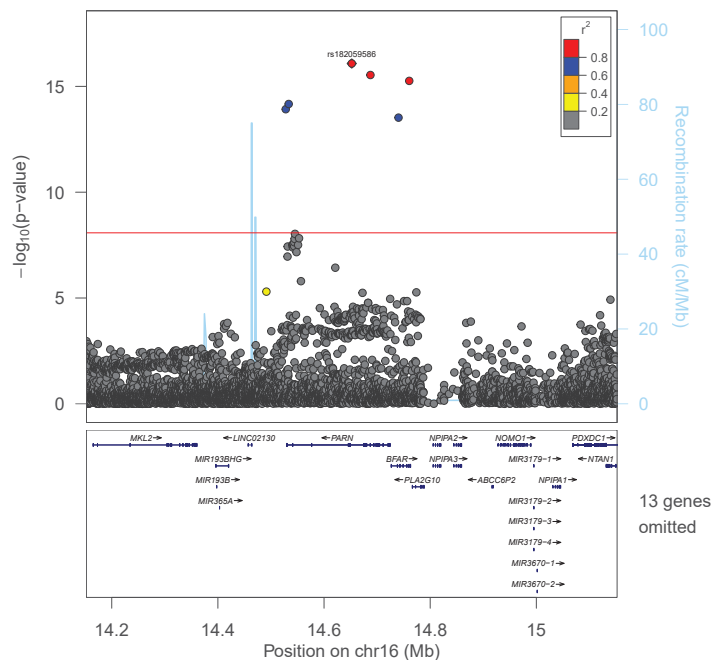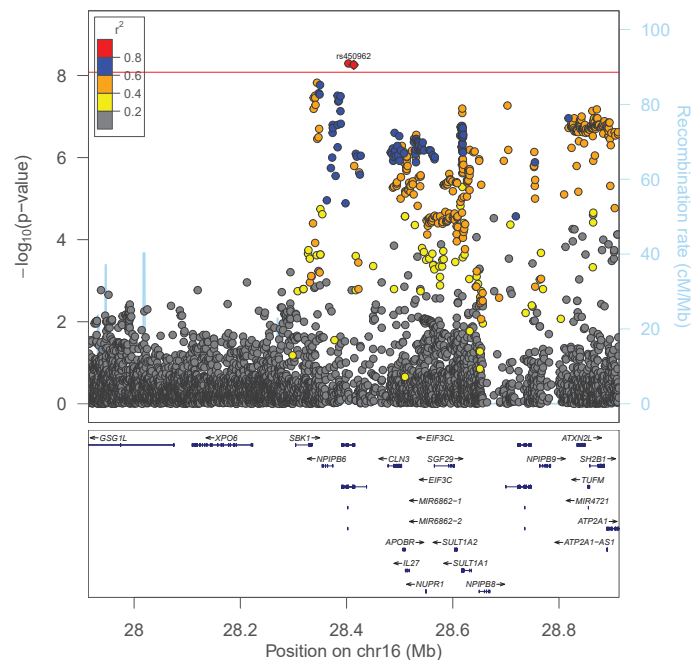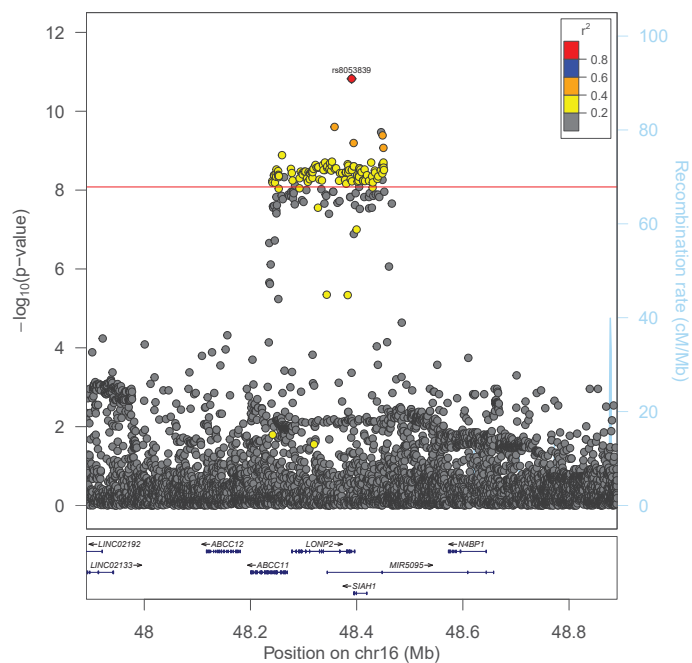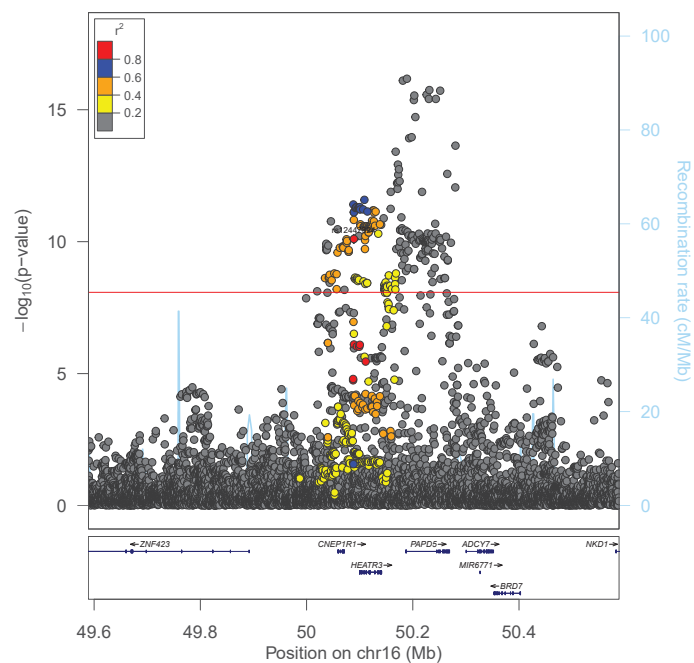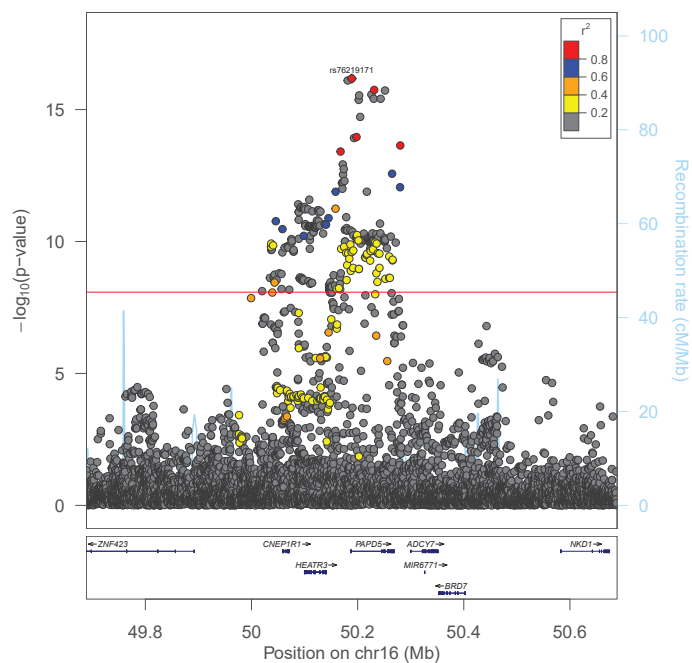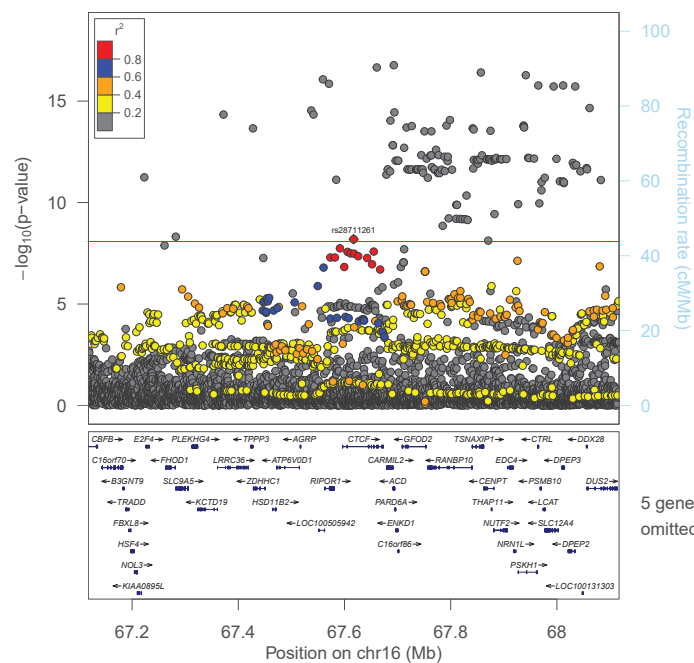

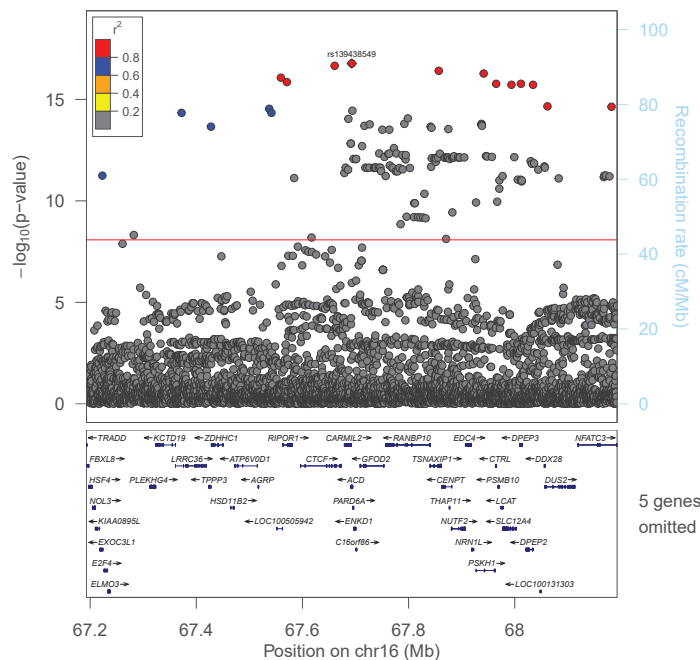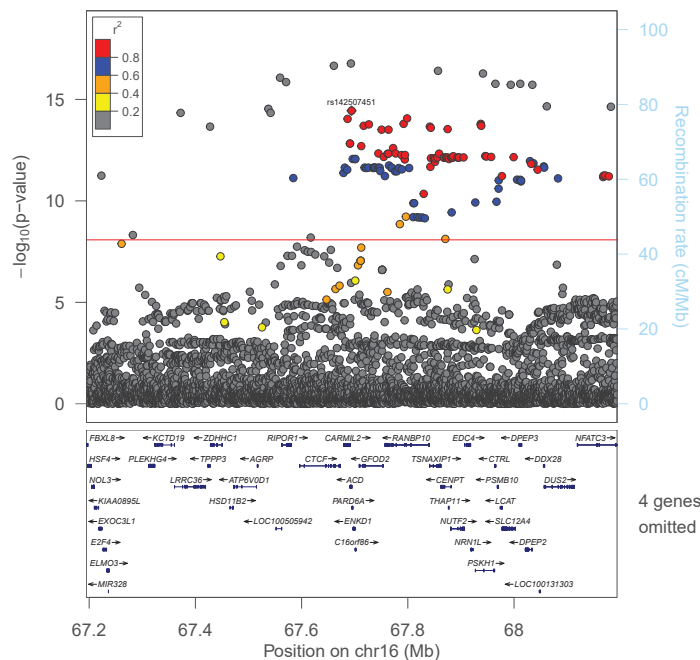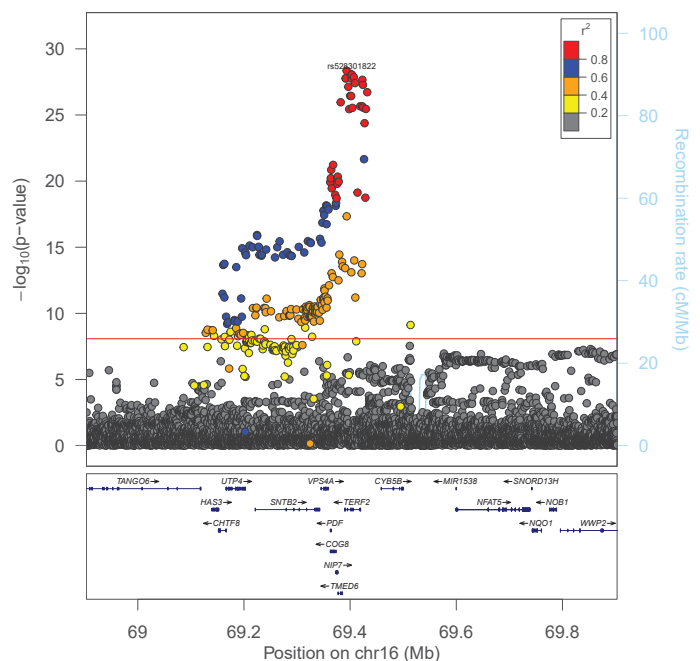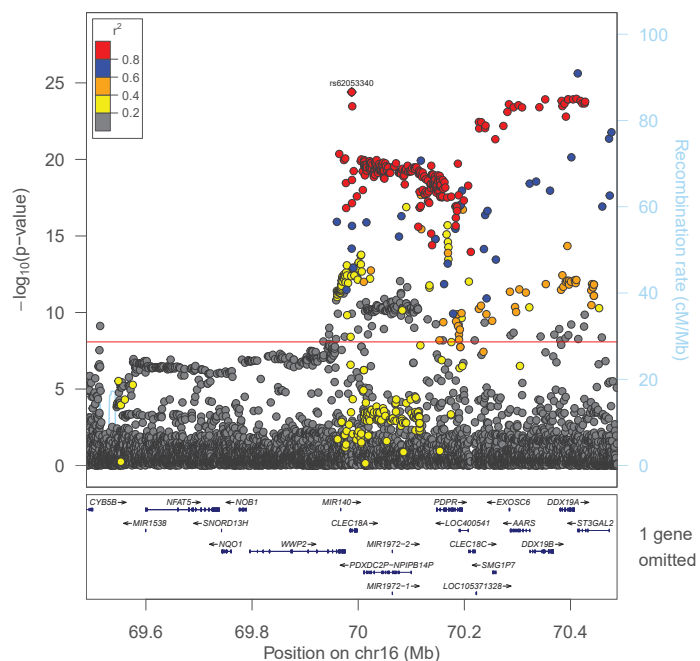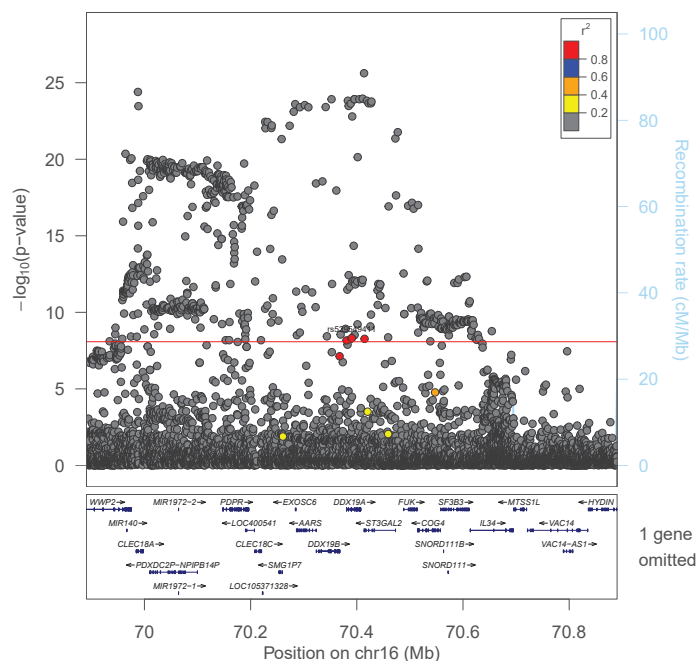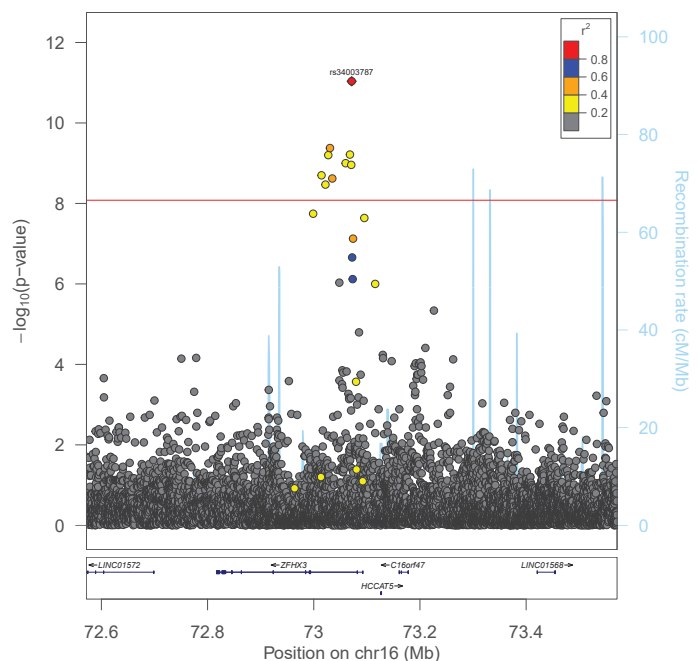

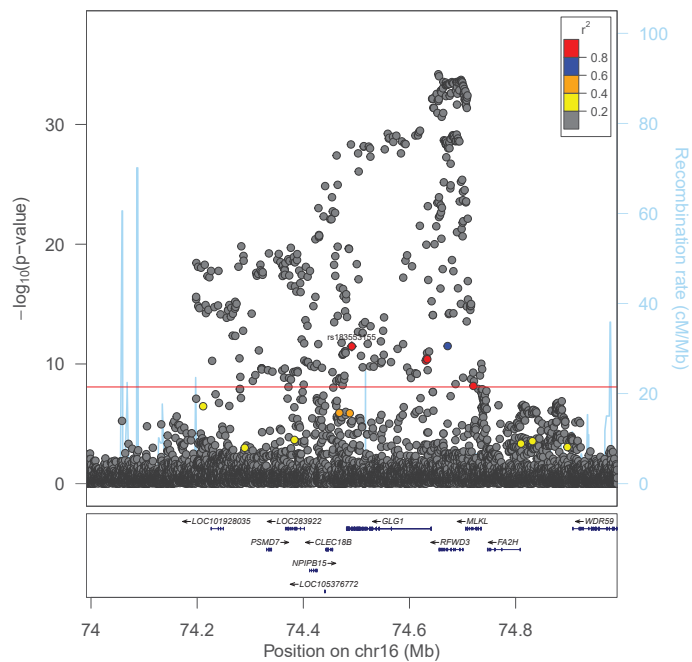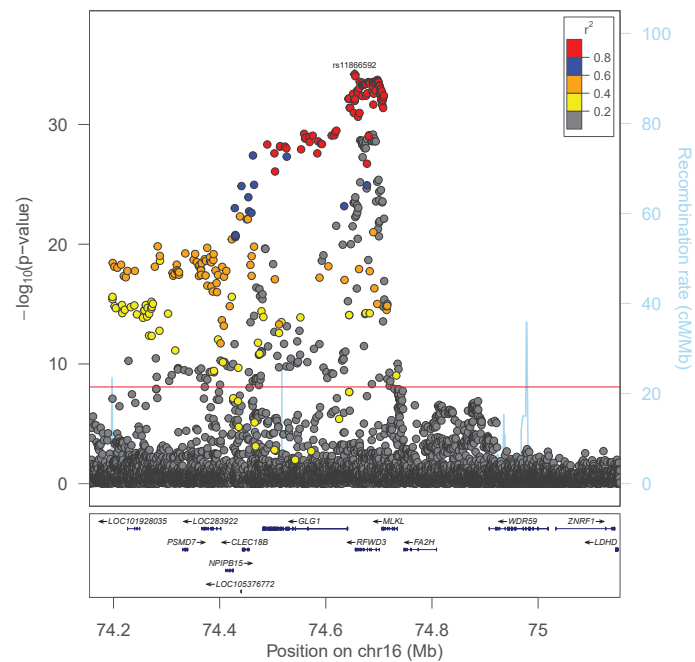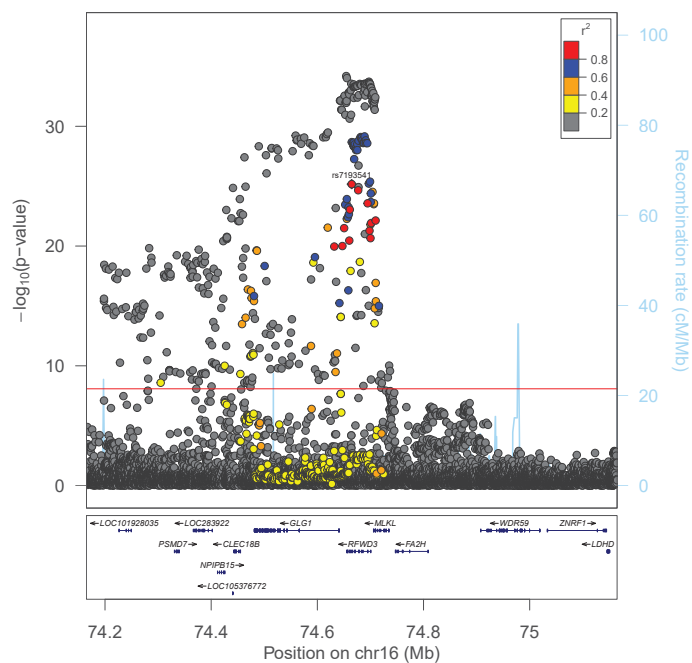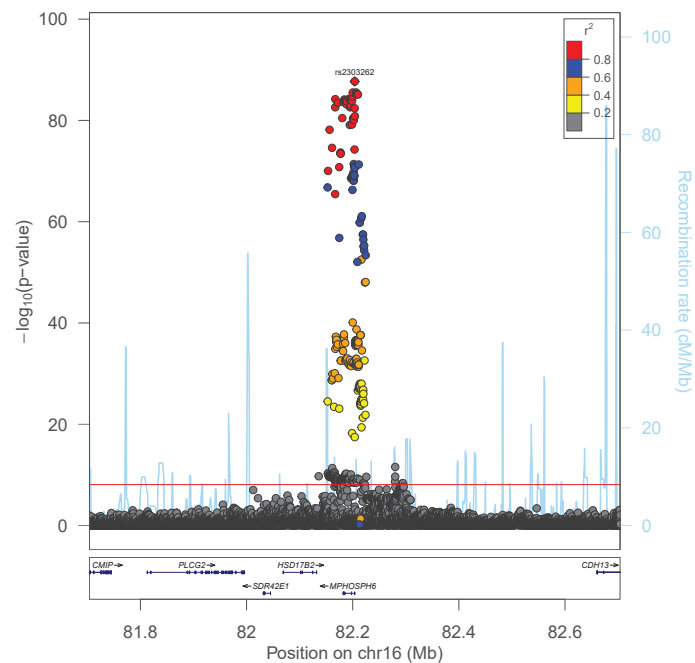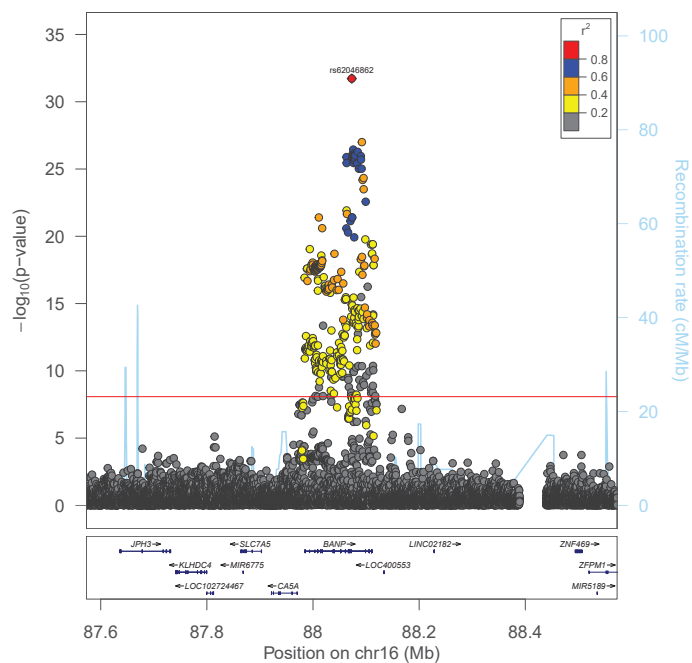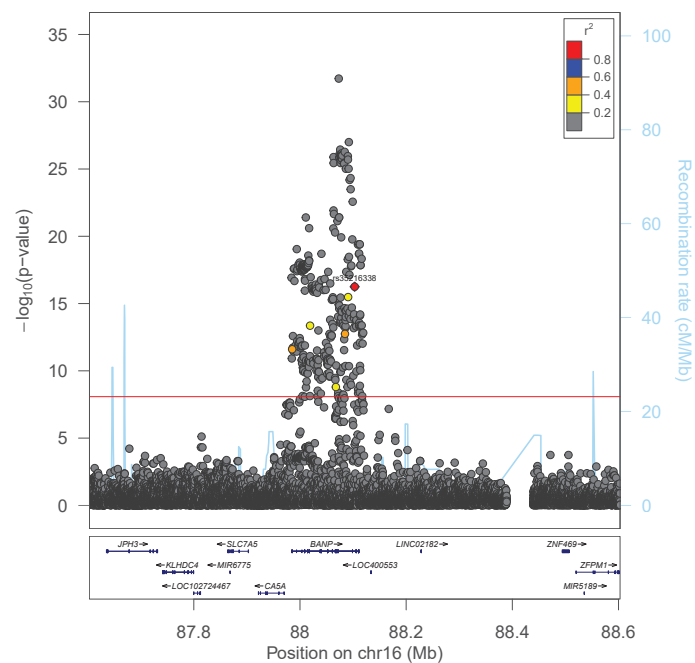

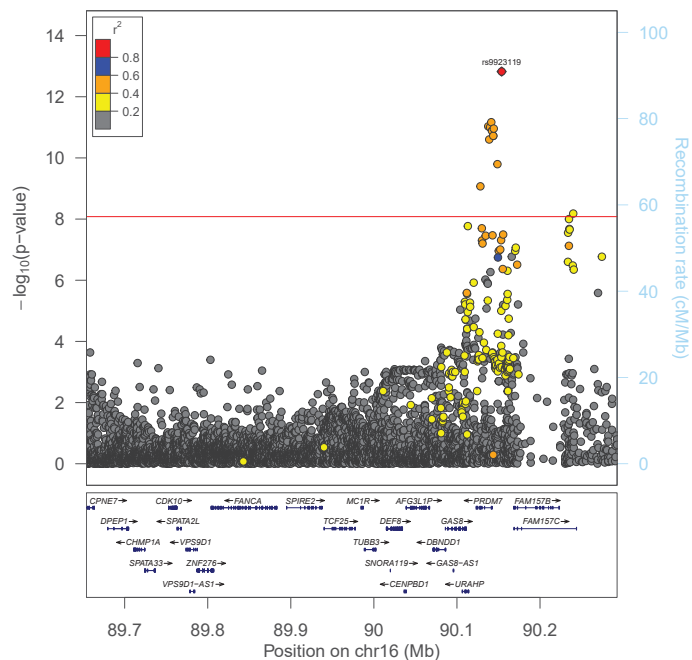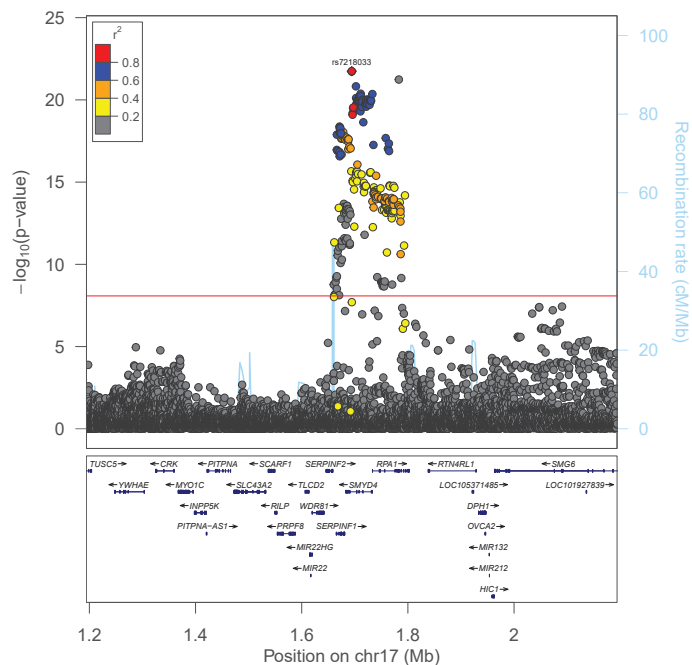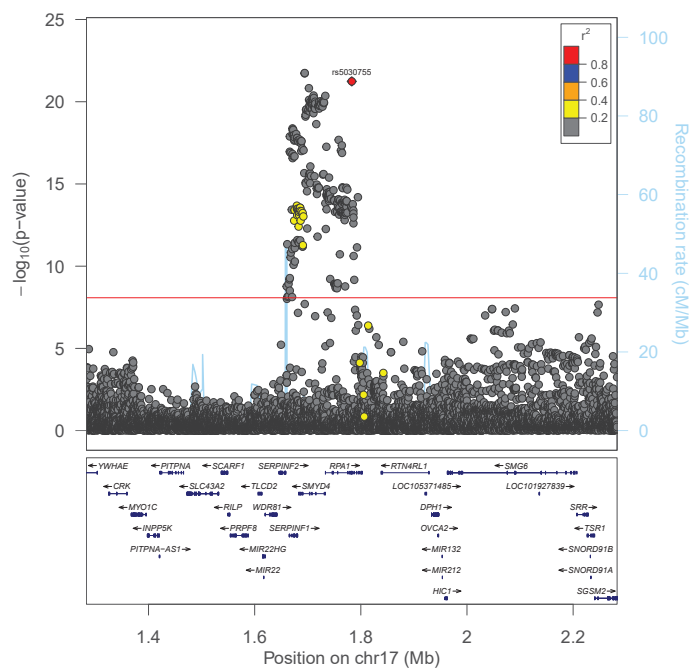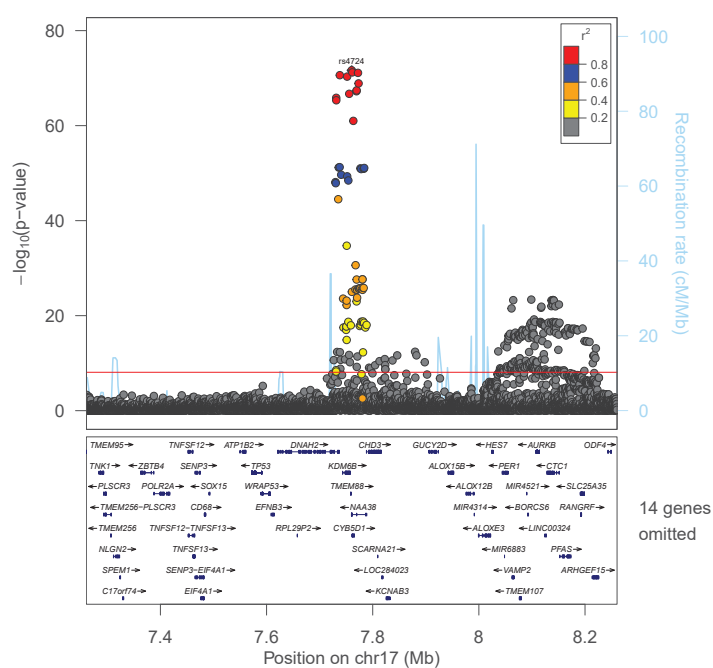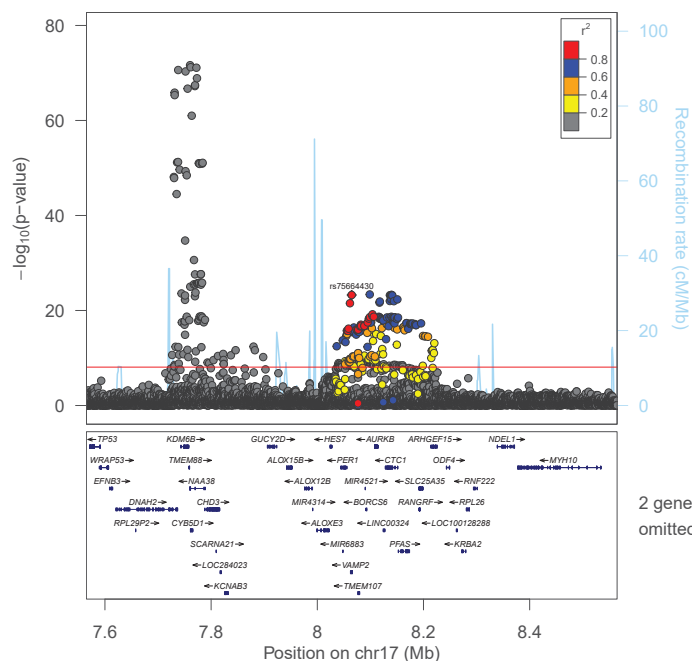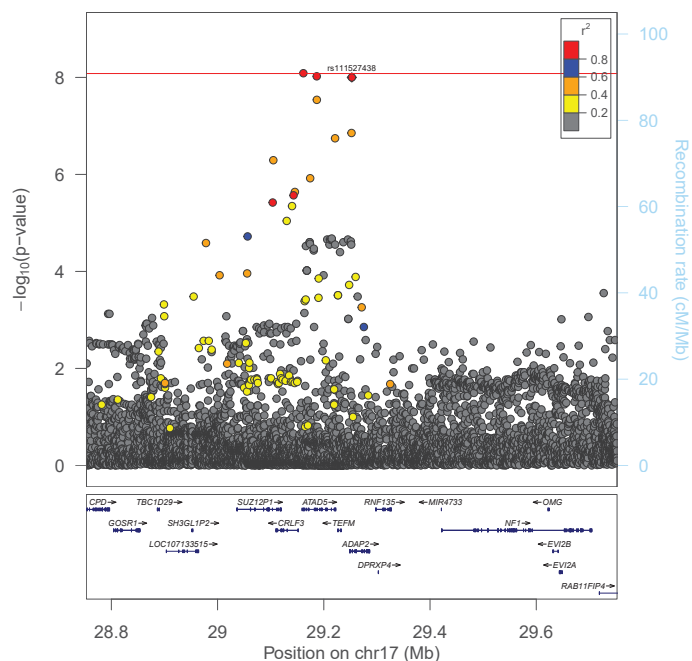

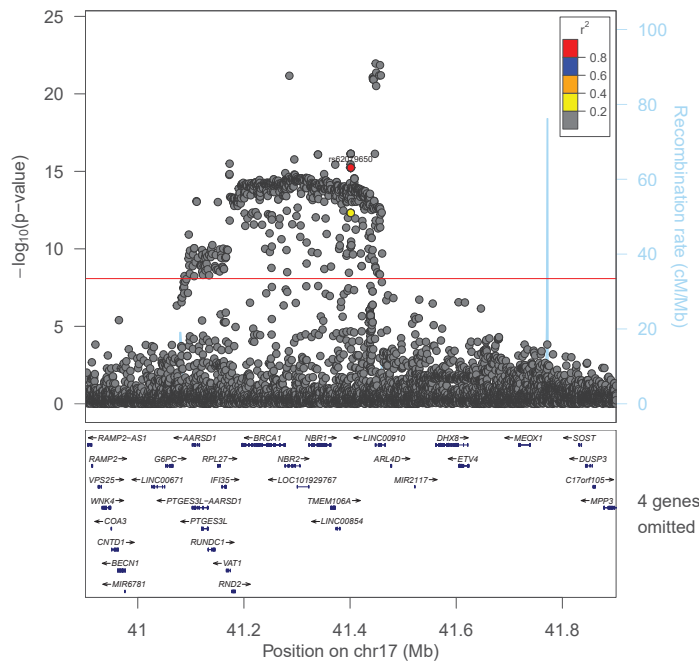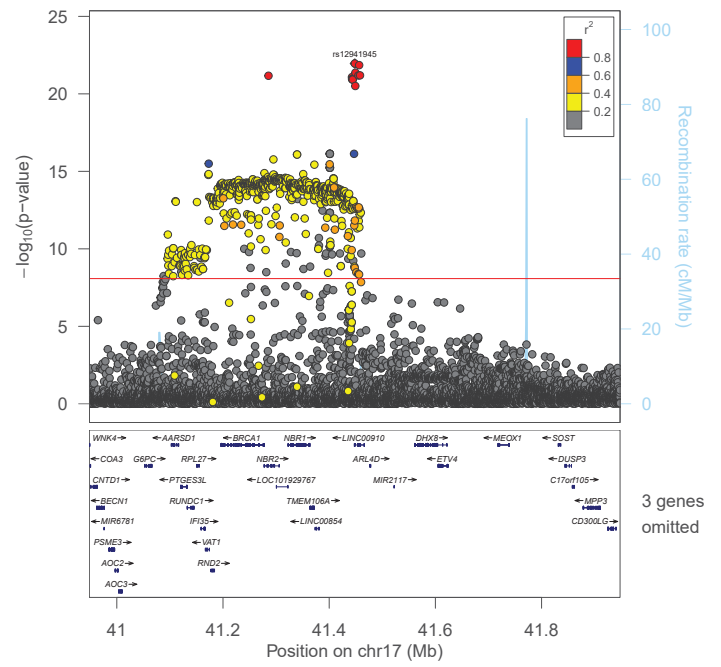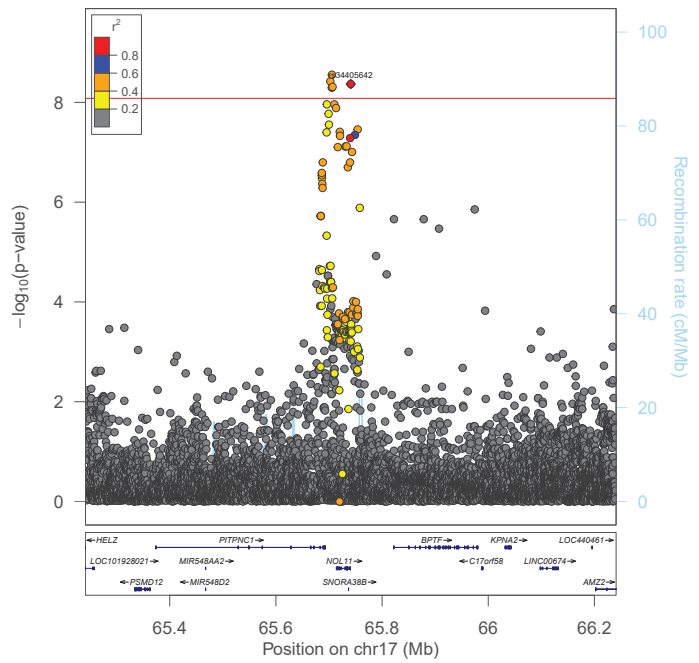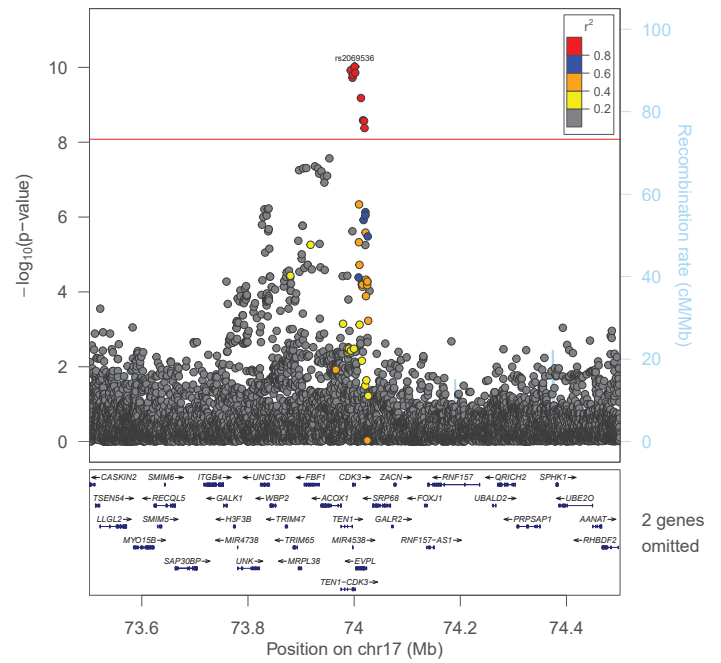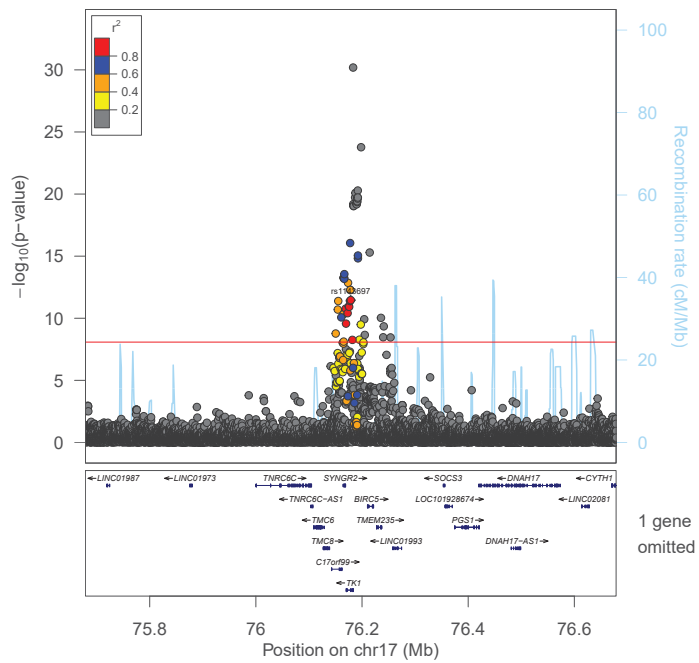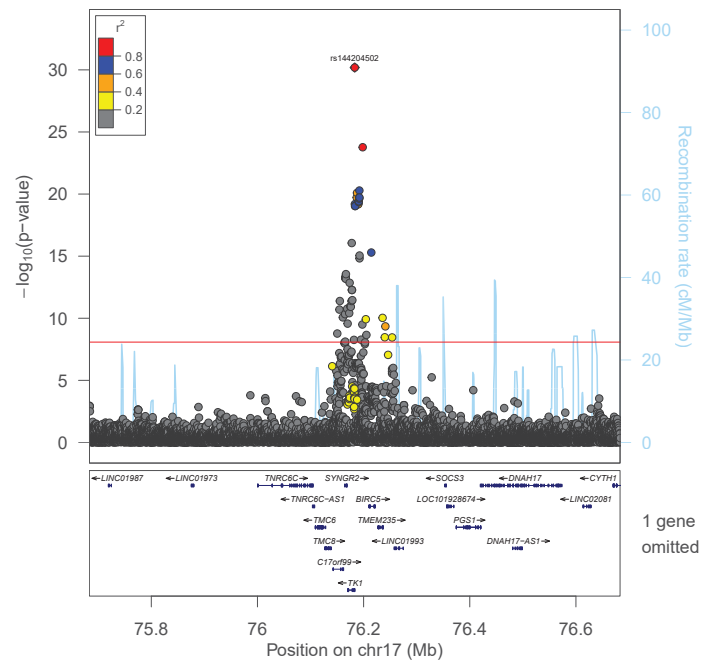

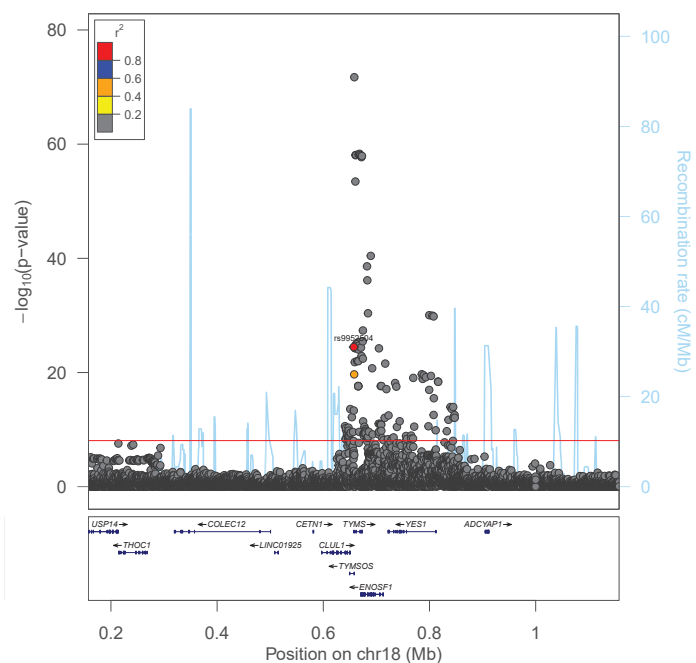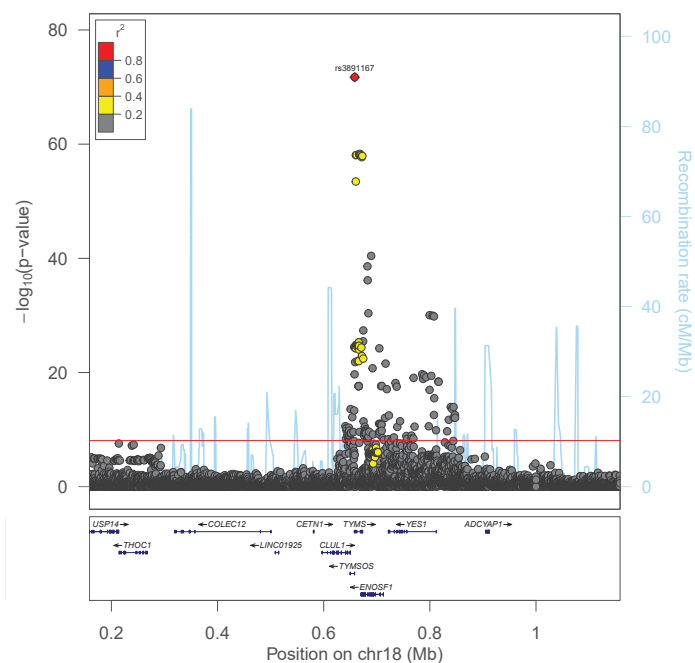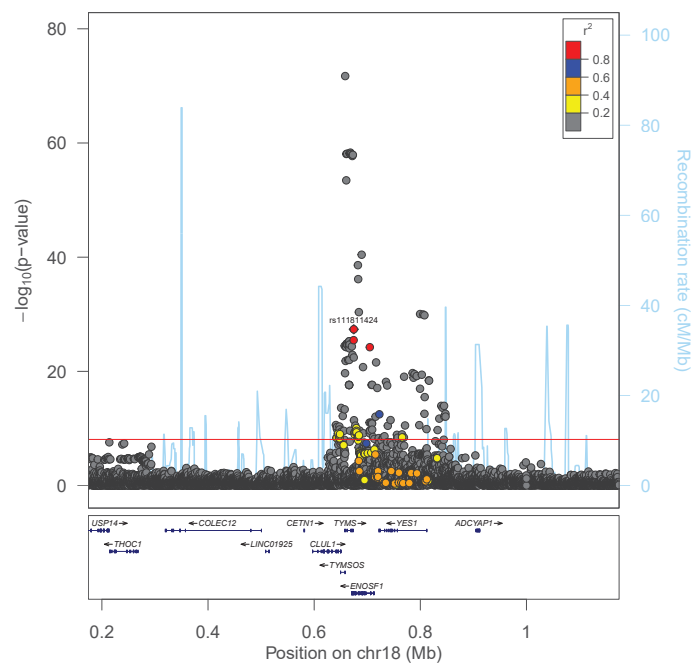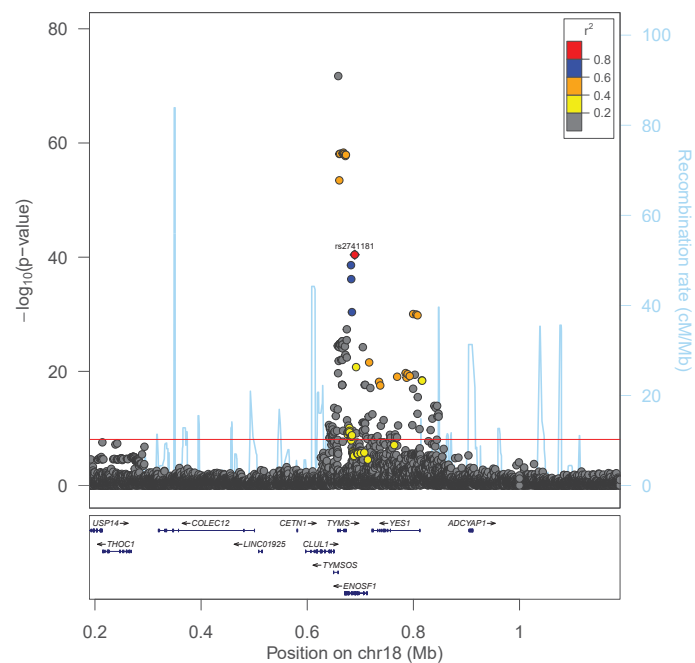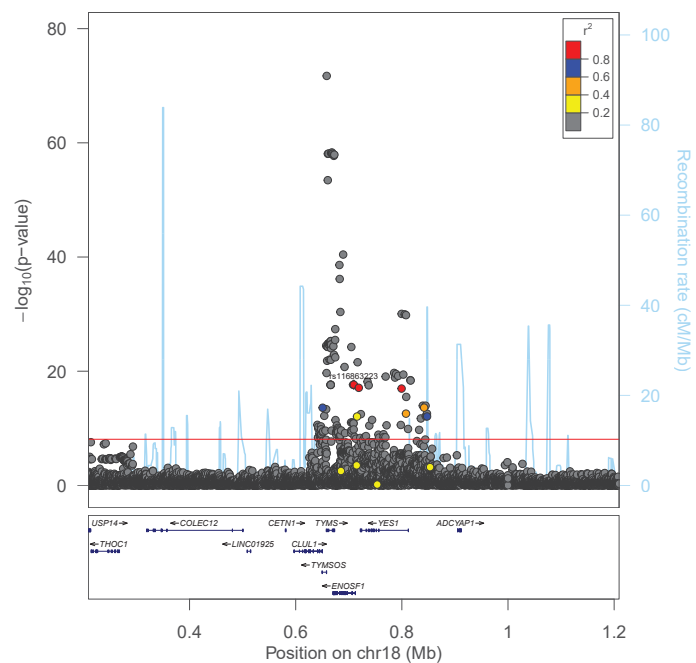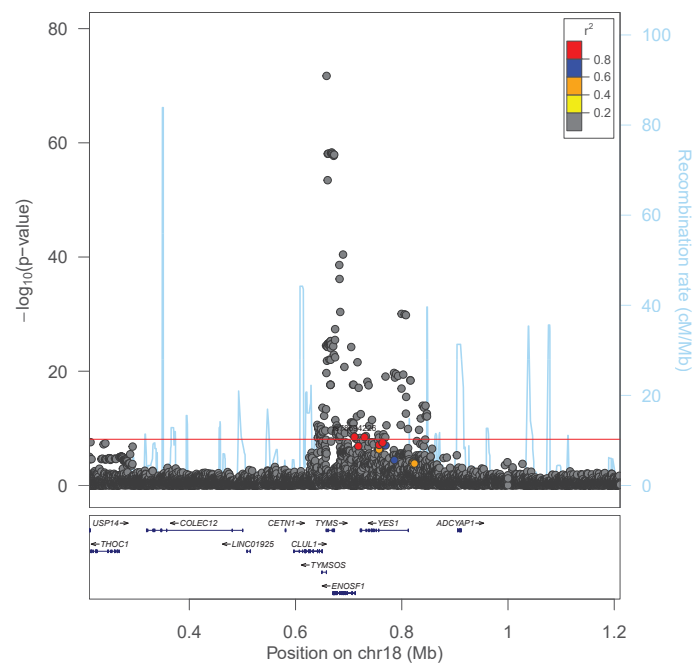

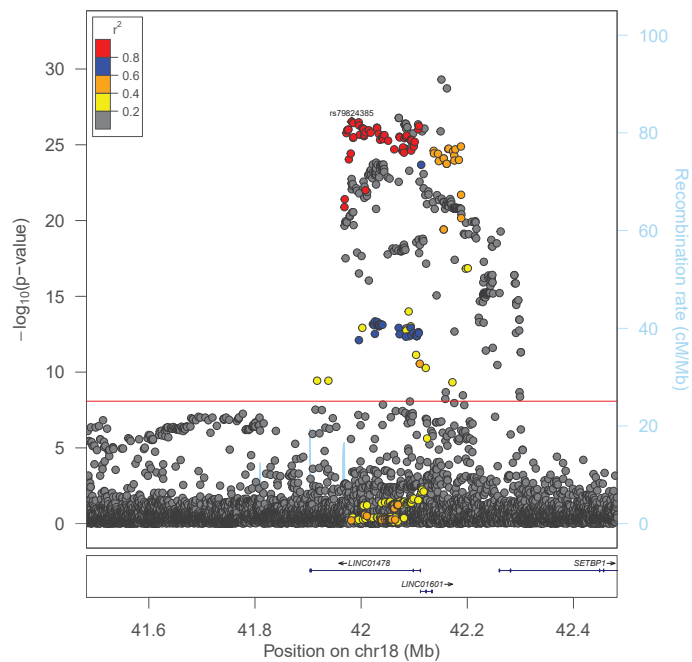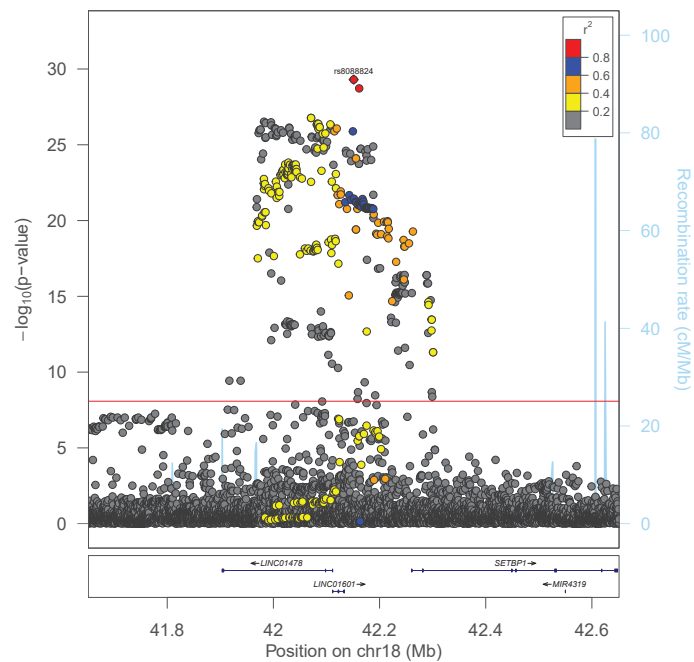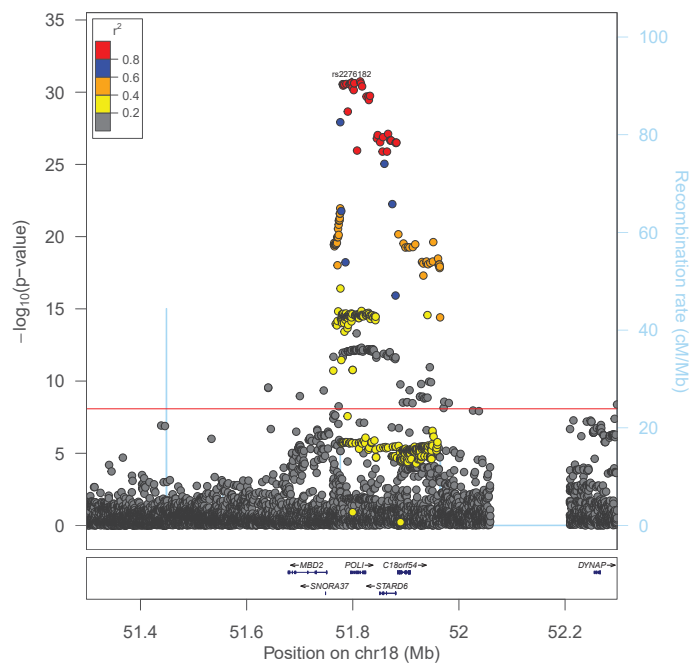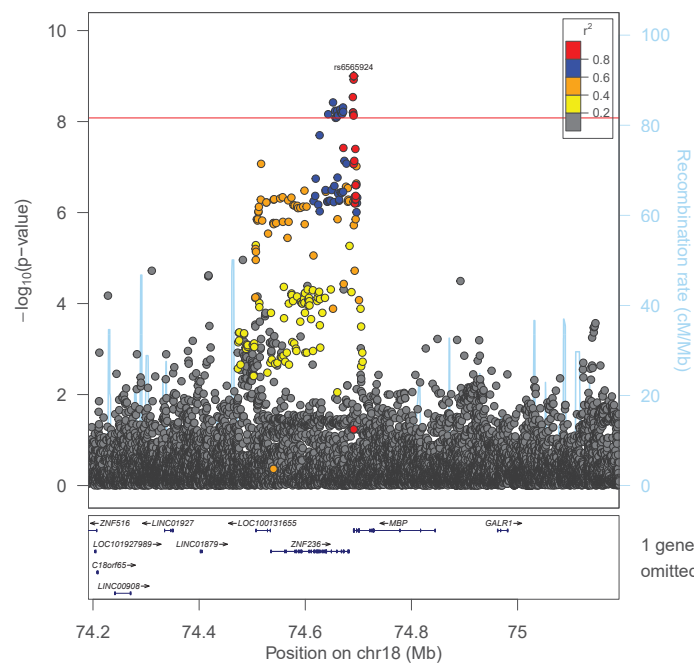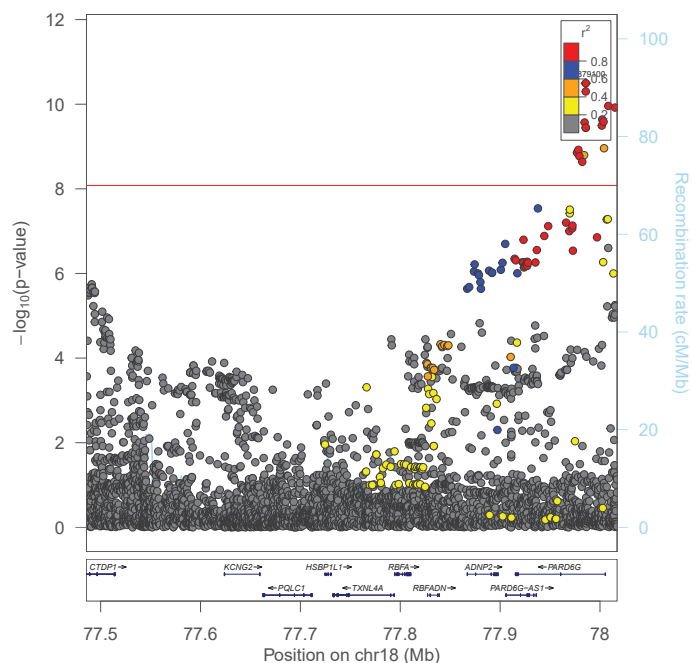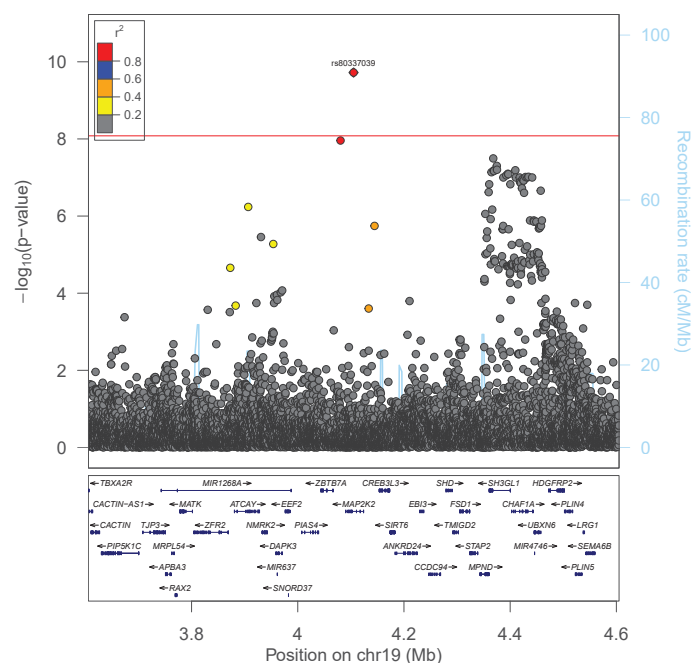

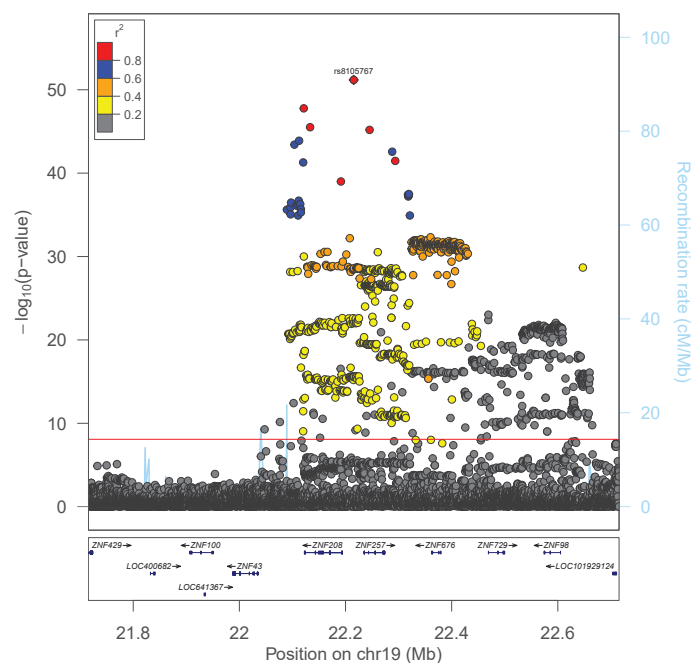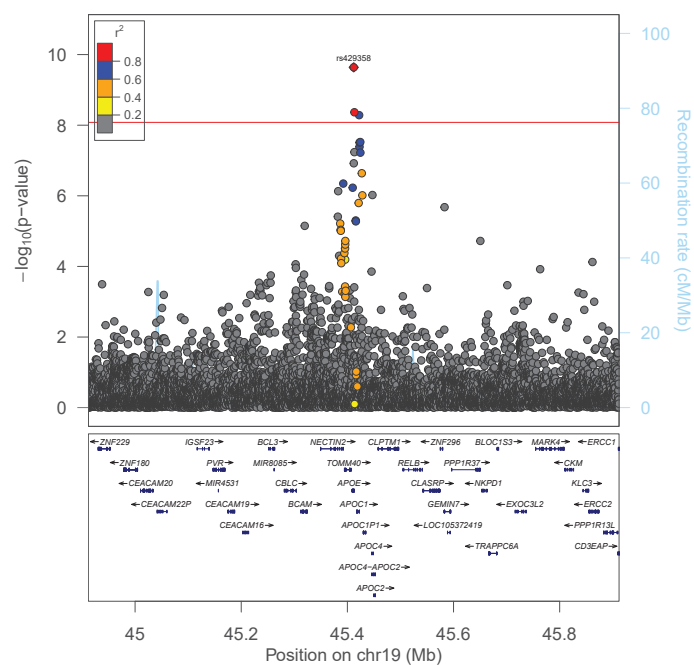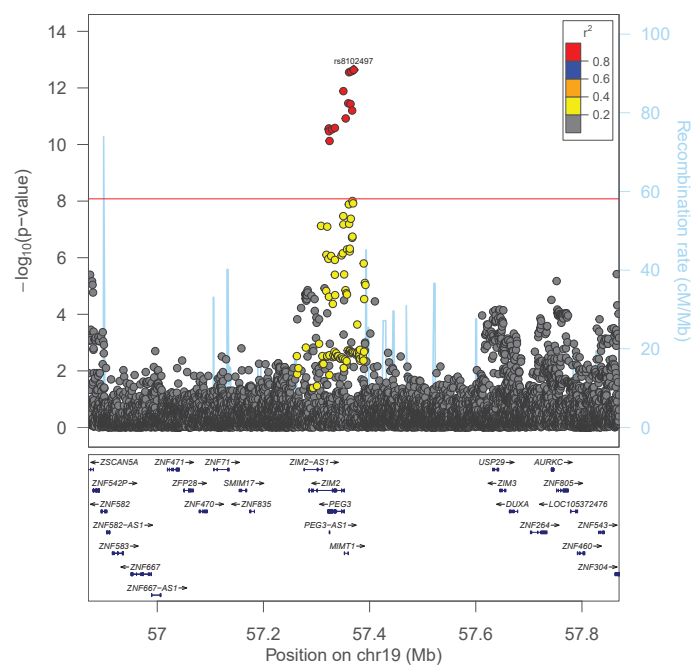

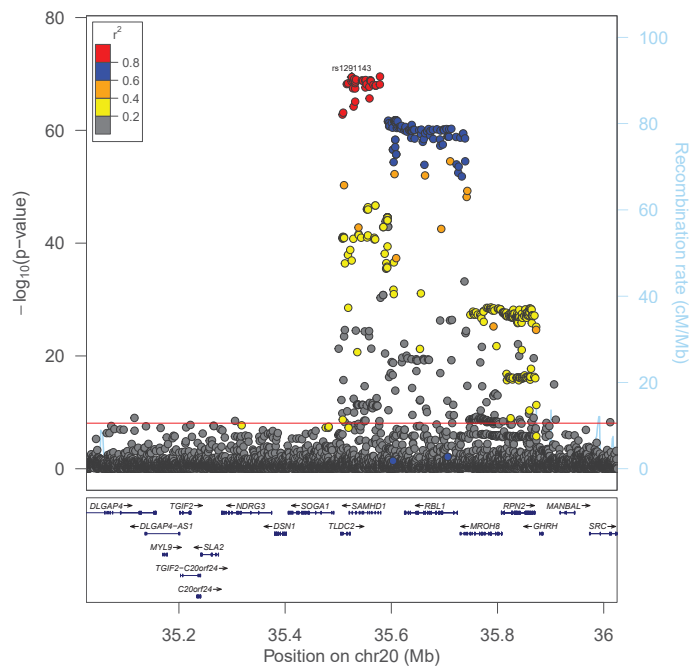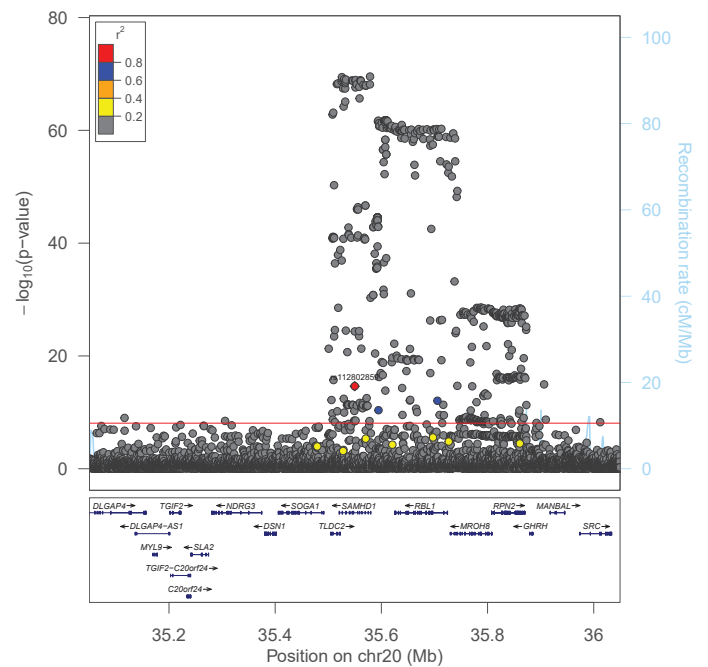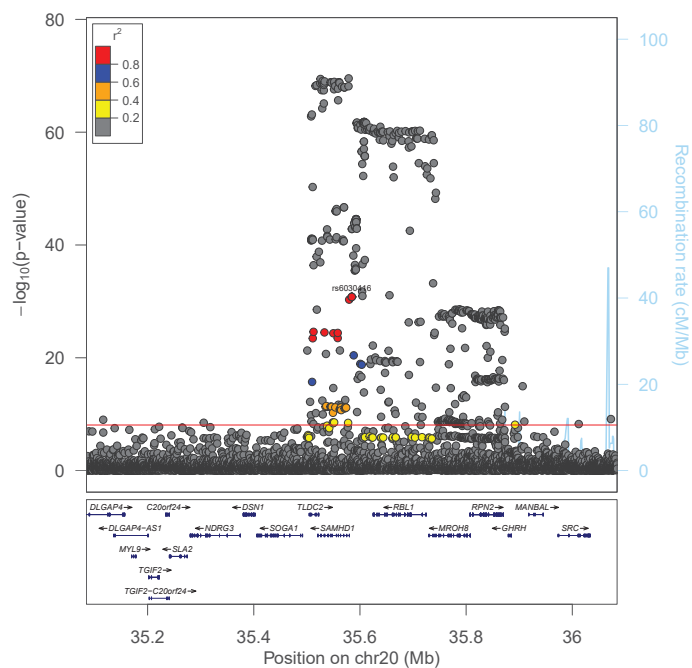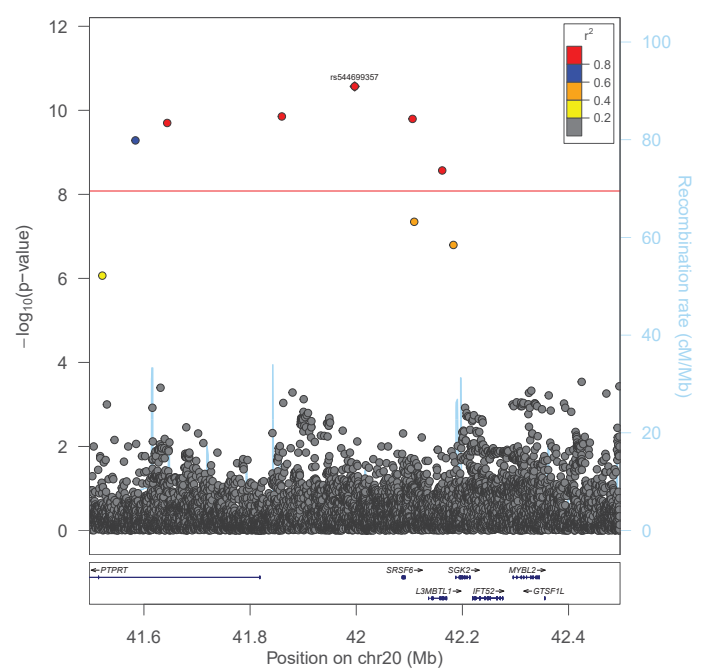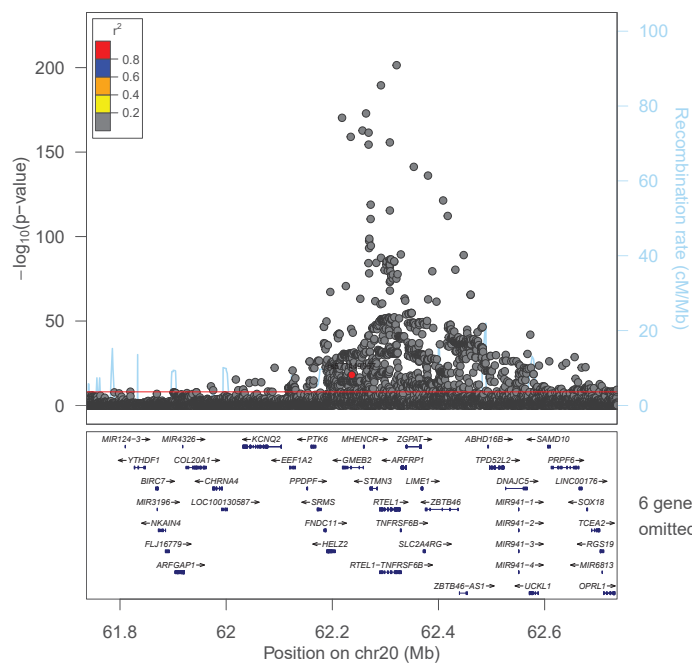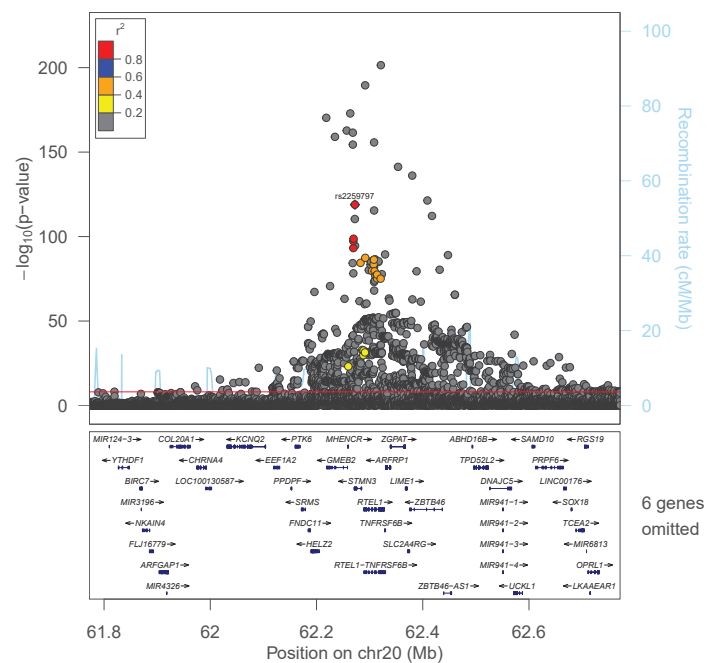

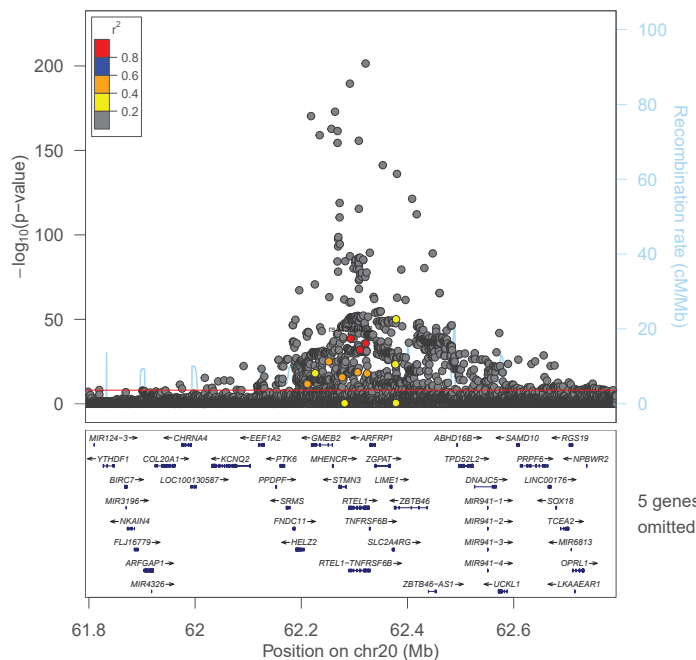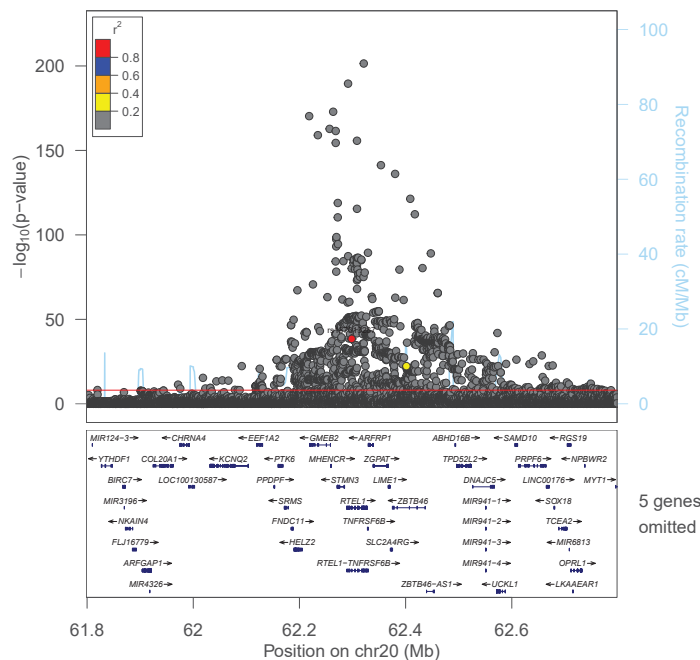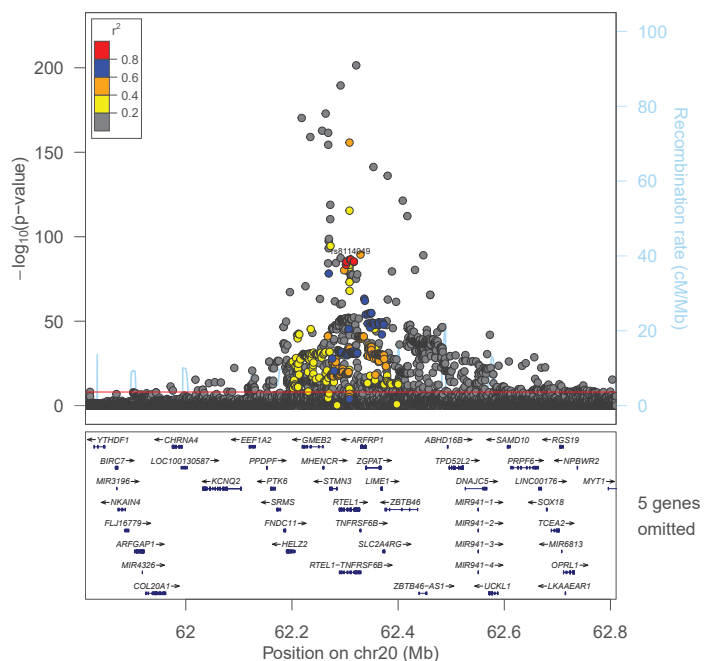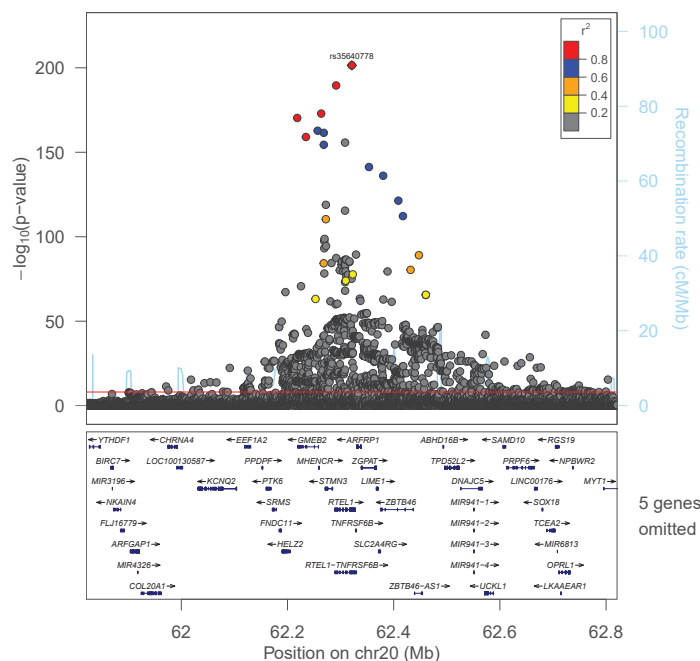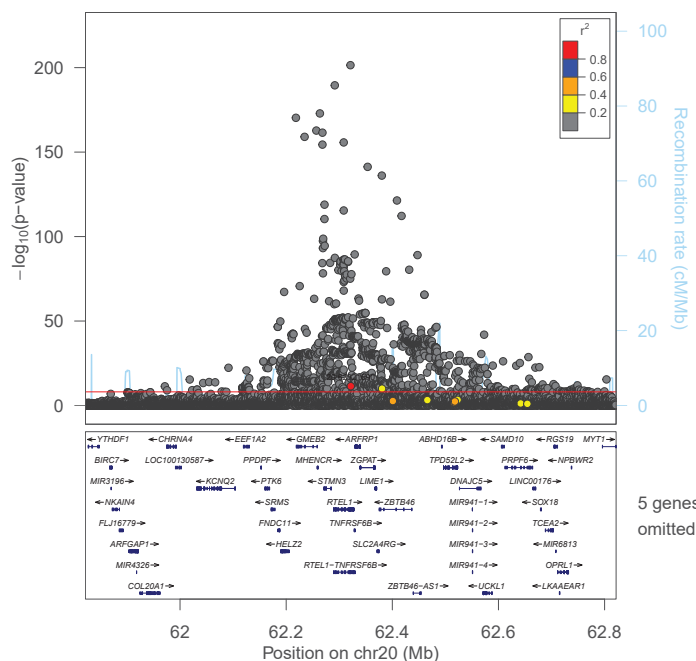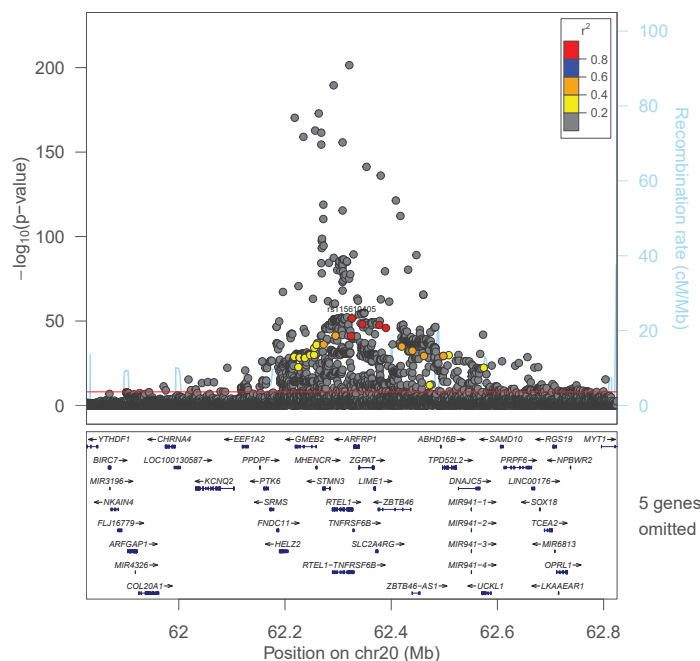

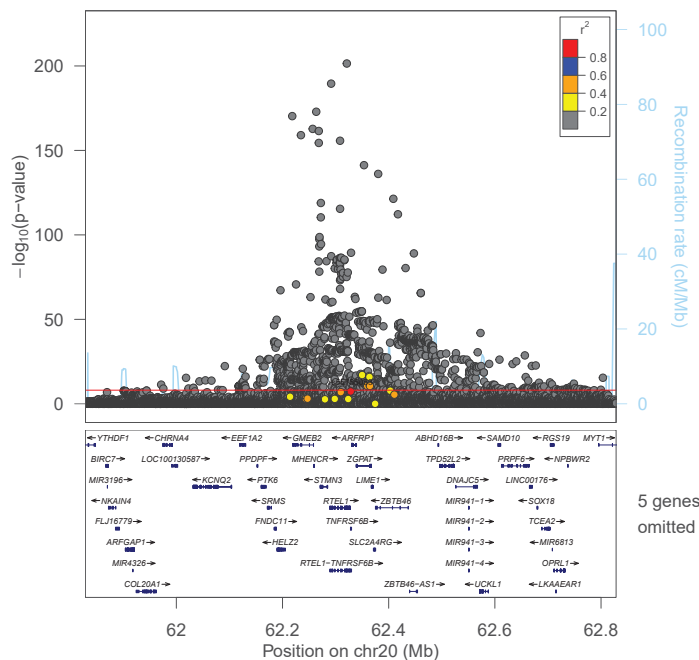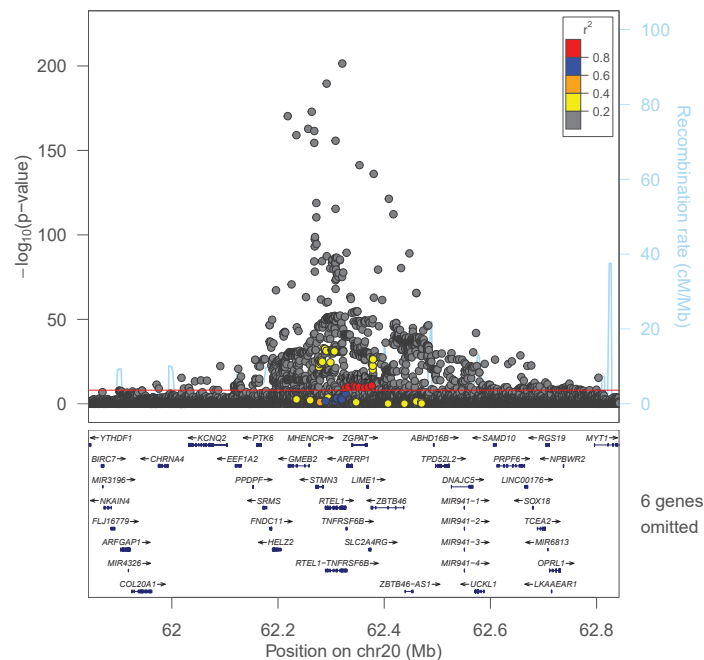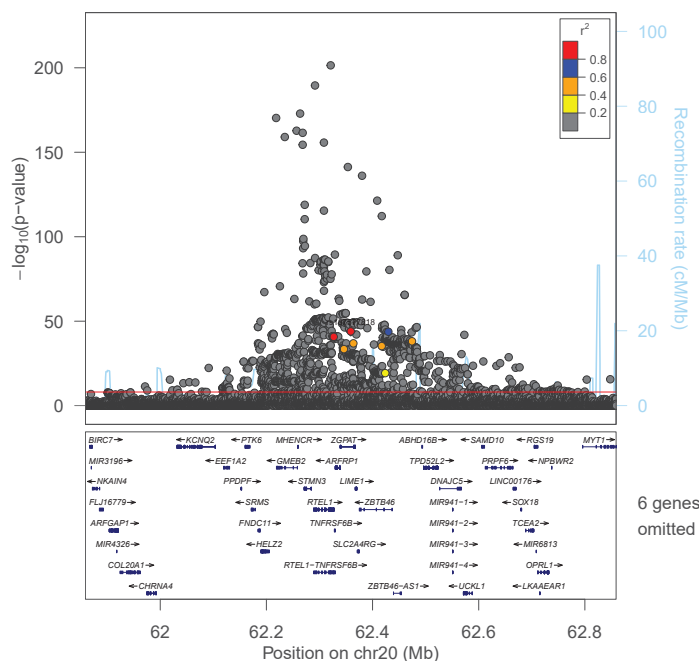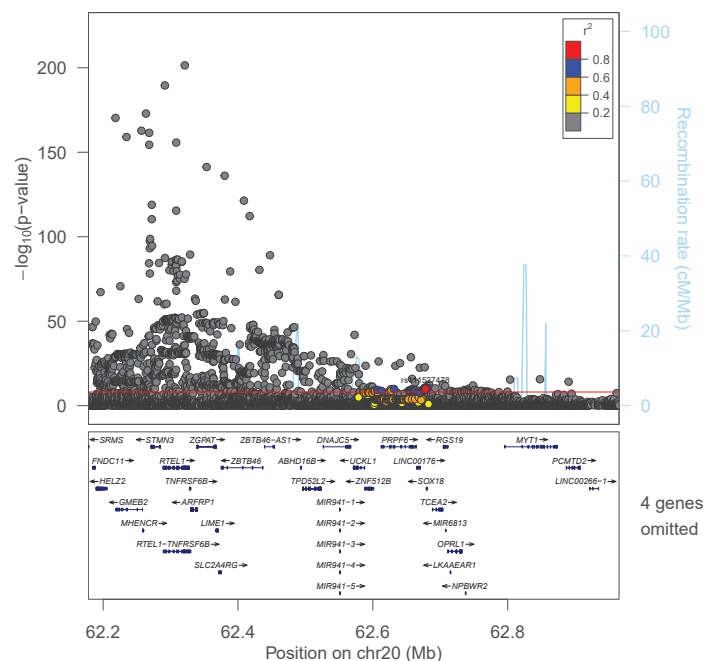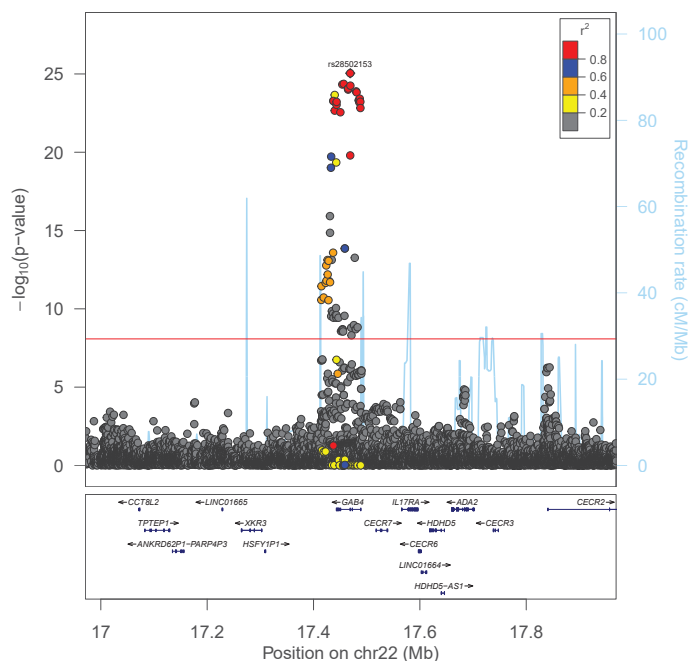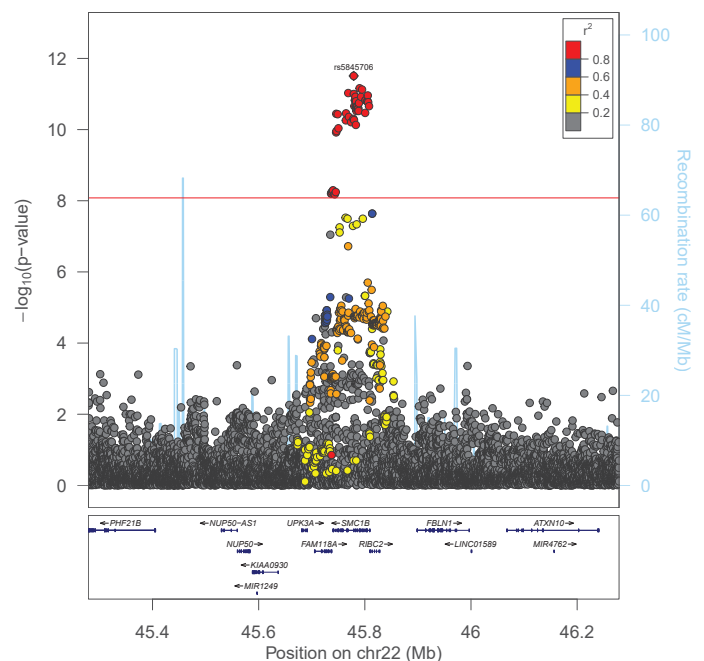

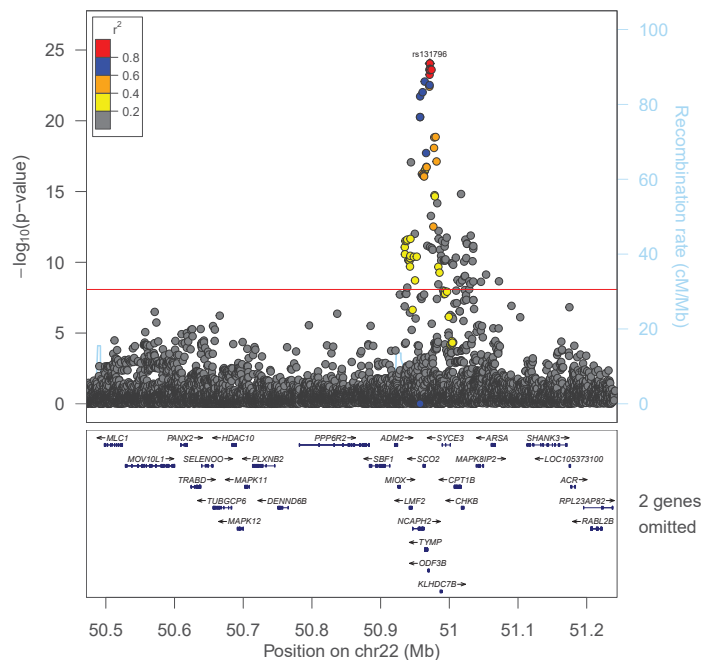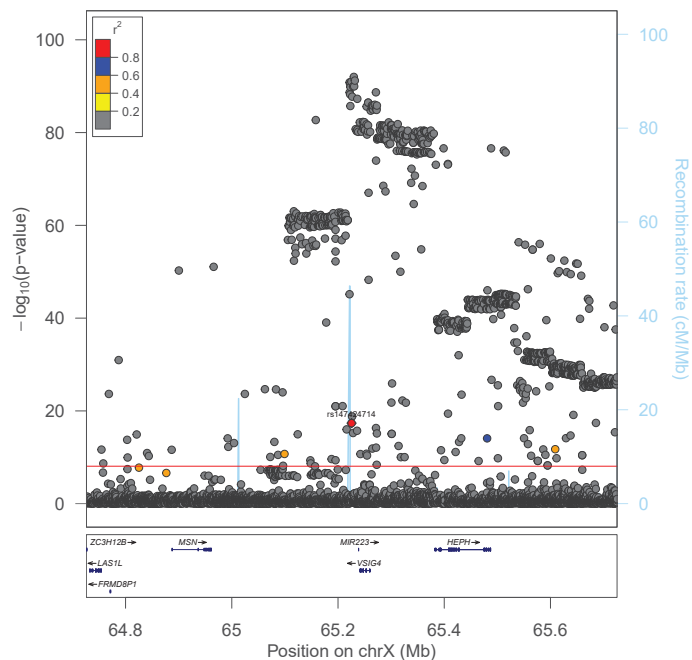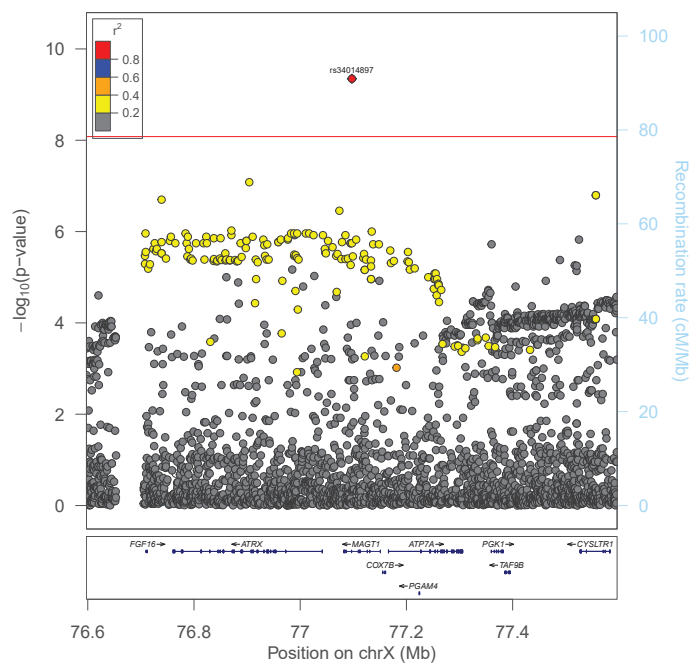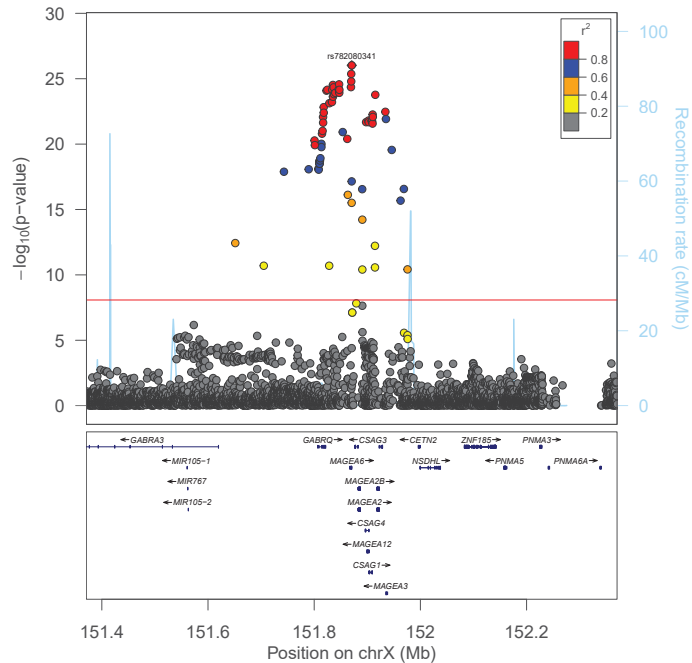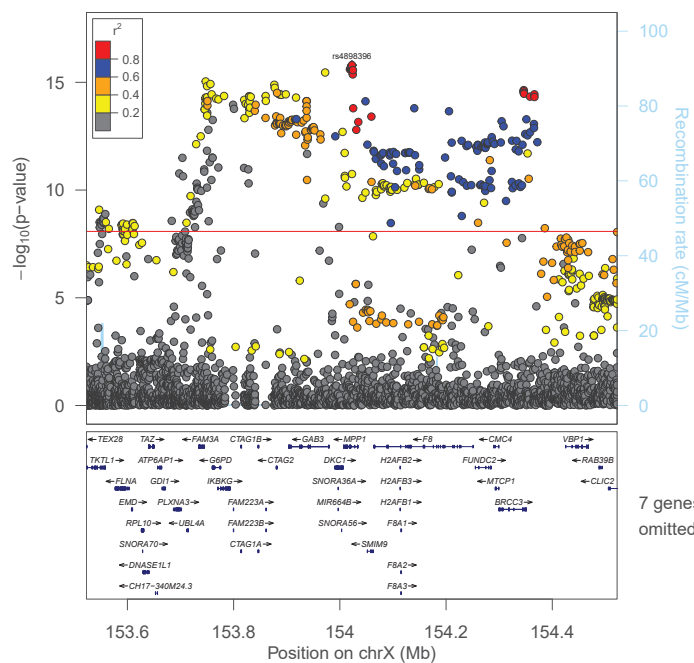

Supplement: Supplementary file 3 — Regional association plots for GWAS sentinels. Plots are shown for each independent GWAS sentinel with a 1 Mb window. [file 41588_2021_944_MOESM3_ESM.pdf]
