# Supplementary material for: Polygenic basis and biomedical consequences of telomere length variation
Source: Nat Genet. 2021 Oct 5;53(10):1425–33. doi: 10.1038/s41588-021-00944-6 (PMC8492471; doi:10.1038/s41588-021-00944-6)
Supplement: Supplementary file 5 — Peer Review Information [file 41588_2021_944_MOESM5_ESM.pdf]

Peer Review Information

Journal: Nature Genetics
Manuscript Title: Polygenic basis and biomedical consequences of telomere length variation
Corresponding author name(s): Dr Nilesh Samani

Editorial Notes:

**Transferred manuscripts**	This document only contains reviewer comments, rebuttal and decision letters for versions considered at Nature Genetics.

Reviewer Comments & Decisions:

Decision Letter, initial version:

5th April 2021

Dear Nilesh,

Your Article "Polygenic basis and biomedical consequences of telomere length variation" has been seen by three referees. You will see from their comments below that, while they find your work of considerable interest, they have raised some relevant points. We are very interested in the possibility of publishing your study in Nature Genetics, but we would like to consider your response to these points in the form of a revised manuscript before we make a final decision on publication.

To guide the scope of the revisions, the editors discuss the referee reports in detail within the team, including with the chief editor, with a view to identifying key priorities that should be addressed in revision, and sometimes overruling referee requests that are deemed beyond the scope of the current study. In this case, in addition to responding to all referee comments with appropriate clarifications and textual revisions, we particularly ask that you perform additional analyses to resolve the basis of the association signal at the HBB locus and revise the presentation and interpretation of this signal accordingly. We hope you will find this prioritized set of referee points to be useful when revising your study. Please do not hesitate to get in touch if you would like to discuss these issues further.

We therefore invite you to revise your manuscript taking into account all reviewer and editor

comments. Please highlight all changes in the manuscript text file. At this stage we will need you to upload a copy of the manuscript in MS Word .docx or similar editable format.

We are committed to providing a fair and constructive peer-review process. Do not hesitate to contact us if there are specific requests from the reviewers that you believe are technically impossible or unlikely to yield a meaningful outcome.

When revising your manuscript:

*1) Include a "Response to referees" document detailing, point-by-point, how you addressed each referee comment. If no action was taken to address a point, you must provide a compelling argument. This response will be sent back to the referees along with the revised manuscript.

*2) If you have not done so already please begin to revise your manuscript so that it conforms to our Article format instructions, available
here.
Refer also to any guidelines provided in this letter.

*3) Include a revised version of any required Reporting Summary:
https://www.nature.com/documents/nr-reporting-summary.pdf
It will be available to referees (and, potentially, statisticians) to aid in their evaluation if the manuscript goes back for peer review.
A revised checklist is essential for re-review of the paper.

Please be aware of our guidelines on digital image standards.

Please use the link below to submit your revised manuscript and related files:

[REDACTED]

Note: This URL links to your confidential home page and associated information about manuscripts you may have submitted, or that you are reviewing for us. If you wish to forward this email to co-authors, please delete the link to your homepage.

We hope to receive your revised manuscript within 4-8 weeks. If you cannot send it within this time, please let us know.

Please do not hesitate to contact me if you have any questions or would like to discuss these revisions further.

Nature Genetics is committed to improving transparency in authorship. As part of our efforts in this direction, we are now requesting that all authors identified as 'corresponding author' on published papers create and link their Open Researcher and Contributor Identifier (ORCID) with their account on the Manuscript Tracking System (MTS), prior to acceptance. ORCID helps the scientific community achieve unambiguous attribution of all scholarly contributions. You can create and link your ORCID from the home page of the MTS by clicking on 'Modify my Springer Nature account'. For more information please visit please visit <a

href="http://www.springernature.com/orcid">www.springernature.com/orcid.

We look forward to seeing the revised manuscript and thank you for the opportunity to review your work.

Sincerely,
Kyle

Kyle Vogan, PhD
Senior Editor
Nature Genetics
https://orcid.org/0000-0001-9565-9665

Referee expertise:

Referee #1: Genetics, cancer, telomeres

Referee #2: Genetics, aging, telomeres

Referee #3: Genetics, cancer, GWAS

Reviewers' Comments:

Reviewer #1:
Remarks to the Author:

A tour de force. The results are impactful and contribute greatly to the role of telomere on genetics, influence on chronic diseases, biological and physiological traits and longevity. Strengths include the sheer breadth, LTL measurements on >474k samples, the very large and well characterized participants of the UK biobank, discovery of novel, rare low-frequency variants, and the use of MR to assess causality.

Very comforting that the authors found associations with TERT1 and other known telomere-affecting genes. In addition, the results of TL and chronic disease supports the cancer–CVD trade-off, greater resistance to cancer comes at the cost of greater susceptibility to CVD. Longer telomeres increase the risk of several cancers, while short increase the risk of CAD. The fact that the overall genetic influence on TL was somewhat limited leaves plenty of room for environmental factors to affect it.

This paper contributes and extends our knowledge of the role of telomeres, a biological marker that has captured the imagination of the scientists and the public. Consistency as well as the novelty of the findings are reassuring to the telomere community.

Reviewer #2:

Remarks to the Author:

General Comment
The paper by Codd et al is a landmark in the telomere field; it considerably increases our understanding of the role of telomere length (TL) in human health and disease. Its findings shed a new light on the genetics of leukocyte TL (LTL) in humans. They are also potentially relevant for the role of evolution in fashioning human LTL. These extraordinary results speak for themselves. My first specific comment focuses on potentially important result among many other interesting findings of the paper. Other comments are suggestions for the authors to revise statements that do not accurately reflect, in my view, the current state of population-based telomere research.

Specific Comments

Comment 1. The work found an association of the minor allele of rs334 in the HBB gene with a longer LTL. The authors state that because they used HBB as control gene in their LTL assay, they are uncertain about the rs334-LTL association. I would strongly recommend that they pursue further this specific association. Recent work suggests that polygenic adaptation contributed to the shorter LTL in Whites of European ancestry compared to sub-Saharan Africans (SAAs) (PMID: 26936823). What then might be the reason that LTL is longer by ~ 500 bp in SSAs than in Whites and by ~ 300 base than in Blacks (PMID: 32821950)? Falciparum malaria might be one of several potential factors that contribute to this difference. In this light, the authors should consider further examining the association they observed between rs334 and LTL.

Comment 2. In the first paragraph of the Introduction the authors state: "Extreme shortening of telomeres – due to rare mutations in telomere regulatory genes – causes premature ageing syndromes." This statement is correct, although I suggest that the author cite a more up-to-date reference (PMID: 30760854). Moreover, rare monogenic diseases stemming from highly detrimental mutations in telomere maintenance genes do not impact aging as we know it in the general population. They are more likely to affect highly proliferative cells in the hematopoietic system, the gastrointestinal tract, skin, etc. They are often expressed in bone marrow failure and a cascade of manifestations, including pulmonary fibrosis, that are atypical of aging, particularly vascular aging in the general population. The next sentence referring to associations of LTL with risks for major cancers, CVD and other diseases of aging is also correct. However, the statement suggesting that LTL is a "biomarker of biological age" does not reflect the latest thinking in telomere epidemiology (PMID: 29335375). The refs the authors list in support of their statement underscore the problem of viewing LTL as a biomarker of biological age. The authors themselves have contributed to a body of research showing that when directly measured or genetically imputed, short LTL is associated with atherosclerotic CVD and long LTL is associated with some major cancers. Do they imply that the biological age of individuals at a risk of having these cancers, e.g., sporadic melanoma, lung adenoma, glioma, etc., is younger than their chronological age? I suggest that they modify the text accordingly.

Comment 3. In the same paragraph and later in the text the authors refer to their LTL measurements as "robust" and "validated". The qPCR-based method they use hardly fits these qualifications compared with the 'gold standard' of TL measurements by Southern blotting (PMID: 21824912) or by flow-FISH. Moreover, they generate findings as T/S ratio that might not be easily translatable to bedside medicine. A Telomere Research Network (TRN), sponsored by the NIH, is presently hard at work addressing shortcomings of the qPCR method. The power of the qPCR method lies in its high throughput. The authors leveraged this feature to generate an amazingly large database that offsets

the relatively high measurement error of the method. In fact, major controversies in population-based telomere research about whether, for instance, sex, ancestry and paternal age affect LTL, principally stem from the inappropriate use of the qPCR method in small-size populations. Using the vast LTL data of the UKB, the authors have already resolved these controversies once and for all (doi: https://doi.org/10.1101/2021.03.18.21253457). The present study should therefore serve as a template for the appropriate use of the qPCR method, taking advantage of its high throughput features. I strongly urge the authors to take the opportunity and use their paper and other ones that will certainly follow from the UKB as platforms that drive home this very point.

Comment 4. The authors state (page 7) that they confirmed associations of genetically determined longer LTL with higher blood pressure. Can they provide a reference, given that later in the text they state that they found "significant results in opposing directions for MR and observational analyses"? I am aware of positive association of LTL with left ventricular mass that is often related to systolic hypertension. Observational studies linking LTL with systolic and diastolic blood pressure are conflicting.

Conclusion
This seminal work will transform population-based telomere research.

Abraham Aviv

Reviewer #3:
Remarks to the Author:

Codd and colleagues present a large GWAS investigation in the UK Biobank of germline variants associated with leukocyte telomere length (LTL) as well as an expanded investigation of biologic traits and diseases associated with LTL. Many new loci are discovered, genes and pathways are further examined, and more clarity is provided on associated traits and diseases. The manuscript is well written, employs proper laboratory and statistical methods, and the conclusions are well supported by the results. I have the following comments:

- The article mentions telomere length is heritable, but provides no new estimates on SNP-based heritability in the manuscript. As this is the largest study to data, a heritability estimate would be of interest to report.

- I commend the authors for using the more stringent GWAS significant threshold, but think some text should be added to the main text explaining the reason (i.e., inclusion of low-frequency/rare variants for testing).

- The association with rs334 in HBB seems more like artifact than a true association, especially if other studies that use different single copy genes in the TL assay show little evidence for replication. Unless the authors can provide strong rationale for including I suggest removing this locus from the significant LTL loci to avoid confusion and communication of spurious findings.

- For leukemia there were opposing directions for the observational and MR relationships. This is explained as a "U" relationship with usual LTL, with little additional explanation. I suspect this may be

communicating something interesting about biology in which longer LTL predisposes to leukemia risk, but shorter observed LTL after leukemia suggests clonal expansion of leukemic clones (or other components of leukemia or treatment) may be reducing TL. Leukemia is the only cancer investigated where the relevant tissue is being sampled for TL measurement. Similar observations may also be present in other solid tumors if the tissue of origin was sampled for TL measurement.

- Pyrimidine metabolism is highlighted in the Discussion as a role in LTL, but little is presented on this in the main text. Can the authors expand on why this pathway was chosen over other pathways?

Minor points

- I could not find a Supplementary Figure 3.

- The sentence after POLI and POLN ends in a fragment. Please complete/clarify.

- Figure 1C, is the beta value reported for the minor allele? If so, please state.

Author Rebuttal to Initial comments

Reviewer comments and responses

Reviewer #1:
Remarks to the Author:

A tour de force. The results are impactful and contribute greatly to the role of telomere on genetics, influence on chronic diseases, biological and physiological traits and longevity. Strengths include the sheer breadth, LTL measurements on >474k samples, the very large and well characterized participants of the UK biobank, discovery of novel, rare low-frequency variants, and the use of MR to assess causality.

Very comforting that the authors found associations with TERT1 and other known telomere-affecting genes. In addition, the results of TL and chronic disease supports the cancer–CVD trade-off, greater resistance to cancer comes at the cost of greater susceptibility to CVD. Longer telomeres increase the risk of several cancers, while short increase the risk of CAD. The fact that the overall genetic influence on TL was somewhat limited leaves plenty of room for environmental factors to affect it.

This paper contributes and extends our knowledge of the role of telomeres, a biological marker that has captured the imagination of the scientists and the public. Consistency as well as the novelty of the findings are reassuring to the telomere community.

We thank the reviewer for their appreciative comments.

Reviewer #2:
Remarks to the Author:

General Comment
The paper by Codd et al is a landmark in the telomere field; it considerably increases our understanding of the role of telomere length (TL) in human health and disease. Its findings shed a new light on the genetics of leukocyte TL (LTL) in humans. They are also potentially relevant for the role of evolution in fashioning human LTL. These extraordinary results speak for themselves. My first specific comment focuses on potentially important result among many other interesting findings of the paper. Other comments are suggestions for the authors to revise statements that do not accurately reflect, in my view, the current state of population-based telomere research.

Specific Comments

Comment 1. The work found an association of the minor allele of rs334 in the HBB gene with a longer LTL. The authors state that because they used HBB as control gene in their LTL assay, they are uncertain about the rs334-LTL association. I would strongly recommend that they pursue further this specific association. Recent work suggests that polygenic adaptation contributed to the shorter LTL in Whites of European ancestry compared to sub-Saharan Africans (SAAs) (PMID: 26936823). What then might be the reason that LTL is longer by ~ 500 bp in SSAs than in Whites and by ~ 300 base than in Blacks (PMID: 32821950)? Falciparum malaria might be one of several potential factors that contribute to this difference. In this light, the authors should consider further examining the association they observed between rs334 and LTL.
We thank Professor Aviv for this very insightful suggestion and have therefore further investigated the association between rs334 and LTL. To further explore whether this association may be true or the result of a technical artefact due to the SNP influencing primer binding for the single copy gene measurement, we explored the impact of this SNP on both telomere and single gene copy number by rs334 genotype. As the S measurement essentially controls for input DNA variation in the assay, we expect minimal inter-individual variation in this measurement. If the association between rs334 and LTL is true we would expect no significant difference in S values between rs334 genotypes, but significant difference in T values. If the association is artefactual, due to the S values being influenced, we would expect the opposite. Our analyses revealed an additive effect of the rs334 minor allele on reduced S measurements, whilst showing no effect on T measurements. This strongly supports the hypothesis that the rs334 mutation reduces S primer binding efficiency, leading to underestimation of the S copy number and inflation of the T/S ratio. We therefore feel that this association is artefactual and have modified the following statement in the manuscript:

“As HBB was used as a control gene in our LTL assay, the fidelity of its apparent association with LTL is uncertain.”

to now read:

“As HBB was used as a control gene in our LTL assay, the fidelity of its apparent association with LTL was investigated in further analyses strongly suggesting that this is an artefactual association.”

We have given full details of the additional analyses on pages 2 and 3 of the Supplementary Information.

We extended this analysis to the second SNP in this locus (rs1609812), which also appears to be a false association and have therefore partitioned the results for this locus in Supplementary Table 1 (see also response to reviewer 3) and have removed rs1609812 from our MR analysis and updated the results appropriately.

Furthermore, we assessed the contribution of the technical effect of rs334 on the observed differences in LTL between individuals of black and which ethnicities, which we have reported in a sister paper (Ref 15). As expected, removal of rs334 minor allele carriers, saw an attenuation of the difference in LTL between black and white ethnicities. However, individuals of black ethnicity still had significantly longer LTL than white individuals. Details of this analysis are also given in the Supplementary Information (page 3) and we have added the following paragraph to the main text (Page 5):

“We also investigated the extent to which the technical effect of rs334 explained the observed difference in LTL between participants of Black and White ethnicities in UKB, which we have reported elsewhere[15]. Although, as expected, removal of rs334 minor allele carriers, attenuated the difference in LTL between Black and White participants, Black participants still had significantly longer LTL than White participants (Supplementary Information).”

Comment 2. In the first paragraph of the Introduction the authors state: "Extreme shortening of telomeres − due to rare mutations in telomere regulatory genes – causes premature ageing syndromes." This statement is correct, although I suggest that the author cite a more up-to-date reference (PMID: 30760854).

We have updated the reference for this statement as suggested to:
Shay, J.W., Wright, W.E. Telomeres and telomerase: three decades of progress. Nat Rev Genet 20, 299–309 (2019).

Moreover, rare monogenic diseases stemming from highly detrimental mutations in telomere maintenance genes do not impact aging as we know it in the general population. They are more likely to affect highly proliferative cells in the hematopoietic system, the gastrointestinal tract, skin, etc. They are often expressed in bone marrow failure and a cascade of manifestations, including pulmonary fibrosis, that are atypical of aging, particularly vascular aging in the general population. The next sentence referring to associations of LTL with risks for major cancers, CVD and other diseases of aging is also correct. However, the statement suggesting that LTL is a "biomarker of biological age" does not reflect the latest thinking in telomere epidemiology (PMID: 29335375). The refs the authors list in support of their statement underscore the problem of viewing LTL as a biomarker of biological age. The authors themselves have contributed to a body of research showing that when directly measured or genetically imputed, short LTL is associated with atherosclerotic CVD and long LTL is associated with some major cancers. Do they imply that the biological age of individuals at a risk of having these cancers, e.g., sporadic melanoma, lung adenoma, glioma, etc., is younger than their chronological age? I suggest that they modify the text accordingly.

Although LTL is still widely viewed as a marker of "biological age" we agree that this concept is outdated and that the relationship between LTL and disease is more complex in nature. We have therefore added the above reference (PMID: 29335375) and rephrased our statement within the introduction to read:-
"Although there has been much interest in shorter TL as a biomarker of older biological age[9], it is now apparent that the relationship between TL and disease risk is complex, as both shorter TL and longer TL have been associated with higher risks of different age-associated diseases[4,10-12]"

Comment 3. In the same paragraph and later in the text the authors refer to their LTL measurements as "robust" and "validated". The qPCR-based method they use hardly fits these qualifications compared with the 'gold standard' of TL measurements by Southern blotting (PMID: 21824912) or by flow-FISH. Moreover, they generate findings as T/S ratio that might not be easily translatable to bedside medicine. A Telomere Research Network (TRN), sponsored by the NIH, is presently hard at work addressing shortcomings of the qPCR method. The power of the qPCR method lies in its high throughput. The authors leveraged this feature to generate an amazingly large database that offsets the relatively high measurement error of the method. In fact, major controversies in population-based telomere research about whether, for instance, sex, ancestry and paternal age affect LTL, principally stem from the inappropriate use of the qPCR method in small-size populations. Using the vast LTL data of the UKB, the authors have already resolved these controversies once and for all (doi: https://doi.org/10.1101/2021.03.18.21253457). The present study should therefore serve as a template for the appropriate use of the qPCR method, taking advantage of its high throughput features. I strongly urge the authors to take the opportunity and use their

paper and other ones that will certainly follow from the UKB as platforms that drive home this very point.

We have used the terms “robust” and “validated” to refer to our own use of the qPCR assay at large scale to reflect both the high level of QC undertaken and the confirmatory analyses on age, sex and ethnicity reported in our methodology paper (as highlighted by Professor Aviv above). However, we acknowledge that our statements may be misinterpreted by readers to reflect the qPCR assay in general, which, like other methods of measuring LTL, is influenced by measurement error. We have therefore replaced “robust” with “large-scale” to describe LTL measurements in the context of Biobanks (page 3) and “validated” with “established” which we believe correctly describes this widely used assay (page 4). We have further added that we have performed confirmatory analyses with established phenotypes, referencing the methodology paper, to provide confidence in the measurements, rather than using the descriptor “validated” (page 4).

Comment 4. The authors state (page 7) that they confirmed associations of genetically determined longer LTL with higher blood pressure. Can they provide a reference, given that later in the text they state that they found "significant results in opposing directions for MR and observational analyses"? I am aware of positive association of LTL with left ventricular mass that is often related to systolic hypertension. Observational studies linking LTL with systolic and diastolic blood pressure are conflicting.

We now provide the reference for the study associating longer genetically determined LTL with increased blood pressure (ref 36, Demanelis, Tong and Pierce, 2021) and apologise for its previous omission.

Conclusion
This seminal work will transform population-based telomere research.

Reviewer #3:
Remarks to the Author:

Codd and colleagues present a large GWAS investigation in the UK Biobank of germline variants associated with leukocyte telomere length (LTL) as well as an expanded investigation of biologic traits and diseases associated with LTL. Many new loci are discovered, genes and pathways are further examined, and more clarity is provided on associated traits and diseases. The manuscript is well written, employs proper laboratory and statistical methods, and the conclusions are well supported by the results. I have the

following comments:

- The article mentions telomere length is heritable, but provides no new estimates on SNP-based heritability in the manuscript. As this is the largest study to data, a heritability estimate would be of interest to report.

We agree with the reviewer that this would be of interest for many researchers and have now included this alongside the variance explained:
"The estimated heritability for LTL explained by all variants genome-wide was 8.1% (SD = 0.26)."

We also provide details of the method for calculating this in the Methods section (Page 12)

- I commend the authors for using the more stringent GWAS significant threshold, but think some text should be added to the main text explaining the reason (i.e., inclusion of low-frequency/rare variants for testing).

We agree that the choice of the more stringent threshold should be explained within the main text and have added a sentence to do so, which reads:
"This threshold was set to account for the inclusion of low frequency variants in the analysis[16]."

- The association with rs334 in HBB seems more like artifact than a true association, especially if other studies that use different single copy genes in the TL assay show little evidence for replication. Unless the authors can provide strong rationale for including I suggest removing this locus from the significant LTL loci to avoid confusion and communication of spurious findings.

We agree with the reviewer and further work (see also reply to reviewer 2) also supports this association being the result of a technical artefact. Whilst we also agree that its inclusion could potentially be confusing, as all of the data (genotypes and LTL) are available to other researchers, we feel that the data should be presented and explained to prevent further confusion should others run the GWAS independently and find this association. Furthermore, we feel that it is important to highlight this technical artefact to other researchers who are interested in ethnic differences in LTL and those potentially measuring LTL in multi-ethnic cohorts using the qPCR method. We have therefore added the following statement:

"This locus was, therefore, removed from further analyses; we advise caution in the use of this control gene in future studies of LTL, especially involving participants of black ethnicity."

For additional clarity we have moved this locus to the bottom of Supplementary Table 1 and separated it from the main results. As our further analyses on this locus supports both SNPs

being technical artefacts, we have now also removed rs1609812 from the MR instrument and made appropriate adjustments throughout the manuscript to reflect the updated results. Changes to the results are minimal although we do have one additional nominally significant association in the disease MR (colorectal cancer).

- For leukemia there were opposing directions for the observational and MR relationships. This is explained as a "U" relationship with usual LTL, with little additional explanation. I suspect this may be communicating something interesting about biology in which longer LTL predisposes to leukemia risk, but shorter observed LTL after leukemia suggests clonal expansion of leukemic clones (or other components of leukemia or treatment) may be reducing TL. Leukemia is the only cancer investigated where the relevant tissue is being sampled for TL measurement. Similar observations may also be present in other solid tumors if the tissue of origin was sampled for TL measurement.

The reviewer makes an excellent observation. We agree that the U-shaped relationship of LTL with leukaemia may provide biological insight into what is happening in tumours generally. We have now expanded the relevant results section to include:
"For leukaemia, we observed a U-shaped association with usual LTL (Supplementary Figure 7), which may represent different stages of the disease process within individuals prior to diagnosis. Haematopoietic stem cells with longer TL are more likely to accrue somatic mutations that potentially lead to leukemic transformation[40], whilst subsequent high proliferation rates during clonal expansion and the resulting telomere attrition is consistent with shorter TL in tumours noted in other observational studies[41]."
and also added the following in the Discussion:
In addition, our finding of a U-shaped relationship between usual LTL and leukaemia potentially explains the dual character of the association observed between TL and cancers. The same mechanism may also exist for solid tumours, namely longer TL predisposes to increased risk of cancer (as also supported by the MR analysis) but as tumour cells proliferate, cells within the tumour demonstrate shorter TL[41].

- Pyrimidine metabolism is highlighted in the Discussion as a role in LTL, but little is presented on this in the main text. Can the authors expand on why this pathway was chosen over other pathways?

We acknowledge that our rationale was not clear. The majority of the pathways were directly related to telomere maintenance or DNA replication, repair and recombination. We specifically wanted to highlight novel pathways that were represented by multiple related GO biological processes – TERC regulation and pyrimidine metabolism. Whilst we have shown nucleotide metabolism to be an enriched pathway in our previous study, our current analysis specifically highlights pyrimidine metabolism, with no specific pathways for purine metabolism.

To clarify this we have added a statement to an earlier sentence highlighting that components of the DNA replication, recombination and repair pathways have known roles in telomere maintenance, indicating that these pathways are not novel:

"Other genes of interest in novel loci are involved in DNA replication, recombination and repair, components of which have established roles in telomere maintenance[29]."

We have also given further explanation regarding highlighting those pathways with multiple associated processes:

"Other enriched pathways, represented by multiple gene ontology (GO) biological processes, included box H/ACA snoRNP assembly and snoRNA 3'-end processing, highlighting key components of TERC regulation within the associated loci. Extending our previous identification of the relevance of nucleotide metabolism to LTL,[3] the current analysis more specifically prioritised pyrimidine metabolism through multiple associated GO processes (Supplementary Table 9)."

In addition we have now highlighted both TERC processing and pyrimidine metabolism in the discussion as there was no intention to prioritise pyrimidine metabolism specifically here.

Minor points

- I could not find a Supplementary Figure 3.
We apologise for this. Supplementary Figure 3 was supplied as a separate file due to the size of the file (33 pages containing the regional association plots for the 197 sentinel variants). We have added a statement between Supplementary Figures 2 and 4 to highlight that it is a separate file.

- The sentence after POLI and POLN ends in a fragment. Please complete/clarify.

We thank the reviewer for pointing this out. We have amended the sentence to now read:

"Neither is known to have a direct role in telomere maintenance; however, other DNA polymerases that are involved in translesion repair function in the ALT pathway,[34] suggesting plausible roles for these polymerases in controlling telomere length."

- Figure 1C, is the beta value reported for the minor allele? If so, please state.

We thank the reviewer for highlighting that this information was missing. We can confirm that the beta is for the minor allele. We have now modified the legend for the figure to read:

"Estimated effect sizes for the minor allele (beta) against the minor allele frequency (MAF)."

Decision Letter, first revision:

Our ref: NG-A57184R

12th June 2021

Dear Nilesh,

Thank you for submitting your revised manuscript entitled "Polygenic basis and biomedical consequences of telomere length variation" (NG-A57184R). In light of your responses to referees' comments, we will be happy in principle to publish your study in Nature Genetics as an Article pending final revisions to comply with our editorial and formatting guidelines.

We are now performing detailed checks on your paper and will send you a checklist detailing our editorial and formatting requirements in about a week. Please do not upload the final materials and make any revisions until you receive this additional information from us.

Thank you again for your interest in Nature Genetics Please do not hesitate to contact me if you have any questions.

Sincerely,
Kyle

Kyle Vogan, PhD
Senior Editor
Nature Genetics
https://orcid.org/0000-0001-9565-9665

Final Decision Letter:

In reply please quote: NG-A57184R1 Samani

18th August 2021

Dear Nilesh,

I am delighted to say that your manuscript "Polygenic basis and biomedical consequences of telomere length variation" has been accepted for publication in an upcoming issue of Nature Genetics.

Prior to setting your manuscript, we may make minor changes to enhance the lucidity of the text and with reference to our house style. We therefore ask that you examine the proofs most carefully to

ensure that we have not inadvertently altered the sense of your text in any way.

Once your manuscript is typeset and you have completed the appropriate grant of rights, you will receive a link to your electronic proof via email with a request to make any corrections within 48 hours. If, when you receive your proof, you cannot meet this deadline, please inform us at rjsproduction@springernature.com immediately.

Your paper will be published online after we receive your corrections and will appear in print in the next available issue. You can find out your date of online publication by contacting the Nature Press Office (press@nature.com) after sending your e-proof corrections. Now is the time to inform your Public Relations or Press Office about your paper, as they might be interested in promoting its publication. This will allow them time to prepare an accurate and satisfactory press release. Include your manuscript tracking number (NG-A57184R1) and the name of the journal, which they will need when they contact our Press Office.

Before your paper is published online, we will be distributing a press release to news organizations worldwide, which may very well include details of your work. We are happy for your institution or funding agency to prepare its own press release, but it must mention the embargo date and Nature Genetics. Our Press Office may contact you closer to the time of publication, but if you or your Press Office have any enquiries in the meantime, please contact press@nature.com.

Acceptance is conditional on the data in the manuscript not being published elsewhere, or announced in the print or electronic media, until the embargo/publication date. These restrictions are not intended to deter you from presenting your data at academic meetings and conferences, but any enquiries from the media about papers not yet scheduled for publication should be referred to us.

Please note that Nature Genetics is a Transformative Journal (TJ). Authors may publish their research with us through the traditional subscription access route or make their paper immediately open access through payment of an article-processing charge (APC). Authors will not be required to make a final decision about access to their article until it has been accepted. Find out more about Transformative Journals

<B>Authors may need to take specific actions to achieve compliance with funder and institutional open access mandates. For submissions from January 2021, if your research is supported by a funder that requires immediate open access (e.g. according to Plan S principles) then you should select the gold OA route, and we will direct you to the compliant route where possible. For authors selecting the subscription publication route our standard licensing terms will need to be accepted, including our self-archiving policies. Those standard licensing terms will supersede any other terms that the author or any third party may assert apply to any version of the manuscript.

Please note that Nature Research offers an immediate open access option only for papers that were first submitted after 1 January 2021.

You will not receive your proofs until the publishing agreement has been received through our system.

If you have any questions about our publishing options, costs, Open Access requirements, or our legal forms, please contact ASJournals@springernature.com

If you have posted a preprint on any preprint server, please ensure that the preprint details are updated with a publication reference, including the DOI and a URL to the published version of the article on the journal website.

To assist our authors in disseminating their research to the broader community, our SharedIt initiative provides you with a unique shareable link that will allow anyone (with or without a subscription) to read the published article. Recipients of the link with a subscription will also be able to download and print the PDF.

As soon as your article is published, you will receive an automated email with your shareable link.

You can now use a single sign-on for all your accounts, view the status of all your manuscript submissions and reviews, access usage statistics for your published articles and download a record of your refereeing activity for the Nature journals.

An online order form for reprints of your paper is available at https://www.nature.com/reprints/author-reprints.html. Please let your coauthors and your institutions' public affairs office know that they are also welcome to order reprints by this method.

If you have not already done so, we invite you to upload the step-by-step protocols used in this manuscript to the Protocols Exchange, part of our on-line web resource, natureprotocols.com. If you complete the upload by the time you receive your manuscript proofs, we can insert links in your article that lead directly to the protocol details. Your protocol will be made freely available upon publication of your paper. By participating in natureprotocols.com, you are enabling researchers to more readily reproduce or adapt the methodology you use. Natureprotocols.com is fully searchable, providing your protocols and paper with increased utility and visibility. Please submit your protocol to https://protocolexchange.researchsquare.com/. After entering your nature.com username and password you will need to enter your manuscript number (NG-A57184R1). Further information can be found at https://www.nature.com/nprot/.

Sincerely,
Kyle

Kyle Vogan, PhD
Senior Editor
Nature Genetics
https://orcid.org/0000-0001-9565-9665
